# High-Frequency Occurrence of Surfactin Monomethyl Isoforms in the Ferment Broth of a *Bacillus subtilis* Strain Revealed by Ion Trap Mass Spectrometry

**DOI:** 10.3390/molecules23092224

**Published:** 2018-09-01

**Authors:** Anita Kecskeméti, Attila Bartal, Bettina Bóka, László Kredics, László Manczinger, Kadaikunnan Shine, Naiyf S. Alharby, Jamal M. Khaled, Mónika Varga, Csaba Vágvölgyi, András Szekeres

**Affiliations:** 1 Department of Microbiology, Faculty of Science and Informatics, University of Szeged, Közép fasor 52, H-6726 Szeged, Hungary; kecskemeti.anita@gmail.com (A.K.); bartaloszi@gmail.com (A.B.); boka.tina@gmail.com (B.B.); kredics@bio.u-szeged.hu (L.K.); manczing@bio.u-szeged.hu (L.M.); varga.j.monika@gmail.com (M.V.); mucor1959@gmail.com (C.V.); 2 Department of Botany and Microbiology, College of Science, King Saud University, Riyadh 11451, Saudi Arabia; sshine@ksu.edu.sa (K.S.); nalharbi1@ksu.edu.sa (N.S.A.); gkhaled@ksu.edu.sa (J.M.K.)

**Keywords:** *Bacillus*, cyclic lipopeptides, HPLC-ESI-IT-MS, surfactin

## Abstract

Surfactins are cyclic lipopeptides consisting of a β-hydroxy fatty acid of various chain length and a peptide ring of seven amino acids linked together by a lactone bridge, forming the cyclic structure of the peptide chain. These compounds are produced mainly by *Bacillus* species and possess numerous biological effects such as antimicrobial (antiviral, antibacterial, and antifungal) activities. A mixture of surfactins extracted from *Bacillus subtilis* strain SZMC 6179J was examined by HPLC-ESI-IT-MS technique, enhancing their separation to reveal novel lipopeptide varieties with higher masses and to characterize their structures. During the MS^2^ spectra analyses of their sodiated molecular ions [M + Na]^+^, a previously rarely encountered group of surfactins was detected and two novel types of the group were discovered containing methyl esterified aspartic acid residue in their fifth amino acid position. The relative amounts of these monomethyl isoforms exceeded 35% of the produced surfactin in total. In the *m*/*z* value of 1114, all the detected isoforms possessed aspartic acid 4-methyl ester residue in their fifth amino acid position (C17-[Lxx4, AME5], C18-[AME5]), offering an opportunity to separate a pure fraction of the compound and to study its biological activities in the future.

## 1. Introduction

**Figure 1:**
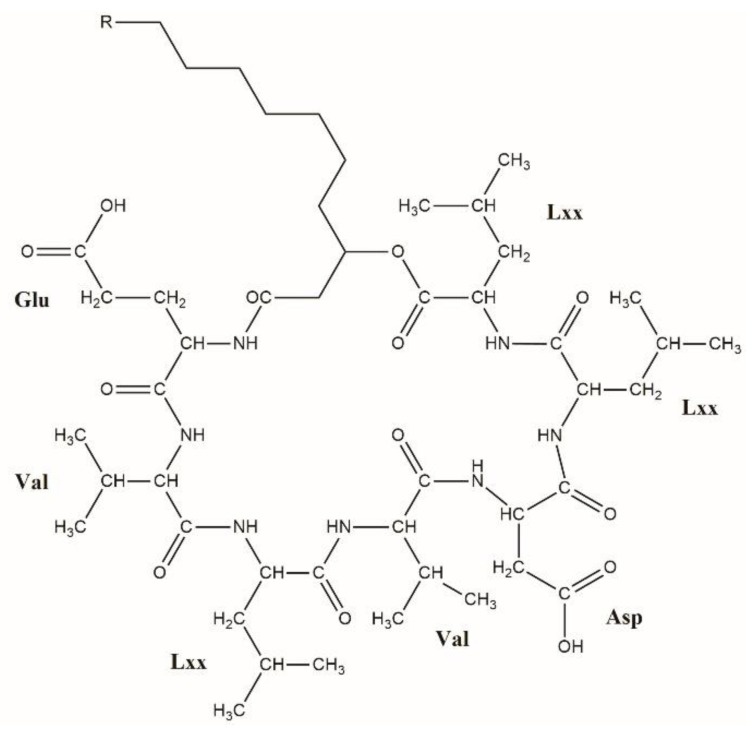
The structure of the surfactin variant [Val2] described by Bóka *et al.* [6]. R = C_3_H_7_–C_5_H_11_.

Surfactins are cyclic lipopeptide-type biosurfactants produced mainly by *Bacillus subtilis,* a Gram-positive bacterium [1]. It consists of a hydrophobic β-hydroxy fatty acid chain of various length (C12–C15) linked to a ring of seven amino acids (Figure 1). The cyclic structure is formed by a lactone bridge between the β-hydroxyl functional group of the fatty acid and the C-terminal of the heptapeptide [2]. Because of its potent surface activity properties [2], surfactin exhibits various biological activities such as antiviral, antitumor, and antimicrobial effects [3], which makes this heptapeptide the subject of various environmental, industrial, and therapeutical investigations [4,5]. The length of the fatty acid chain and the amino acid sequence varies, resulting in numerous isoforms of the compound listed and characterized in previous works [2] (Table 1). Although most of these variants were reported in the 1990s, a recent study from our group introducing a novel surfactin molecule containing Val in the second amino acid position [6] (Figure 1) proved that there may still be surfactin isoforms that are yet to be discovered. Moreover, the incorporations of esterified form of Asp (Asp-*O*-Me, aspartic acid 4-methyl ester—AME) and Glu (Glu-*O*-Me, glutamic acid 5-methyl ester—GME) were also reported occasionally [7,8,9]. In the present study, an HPLC gradient elution method was applied that was capable of separating all lipopeptide components in our sample and to examine the separated, previously hidden, more non-polar fractions by MS^2^ measurements in order to identify the various surfactin molecules, with different amino acid sequences and aliphatic acid chain lengths, produced by *B. subtilis* SZMC 6179J to the full extent.

## 2. Results and Discussions

### 2.1. Separation of Lipopeptides

**Figure 2:**
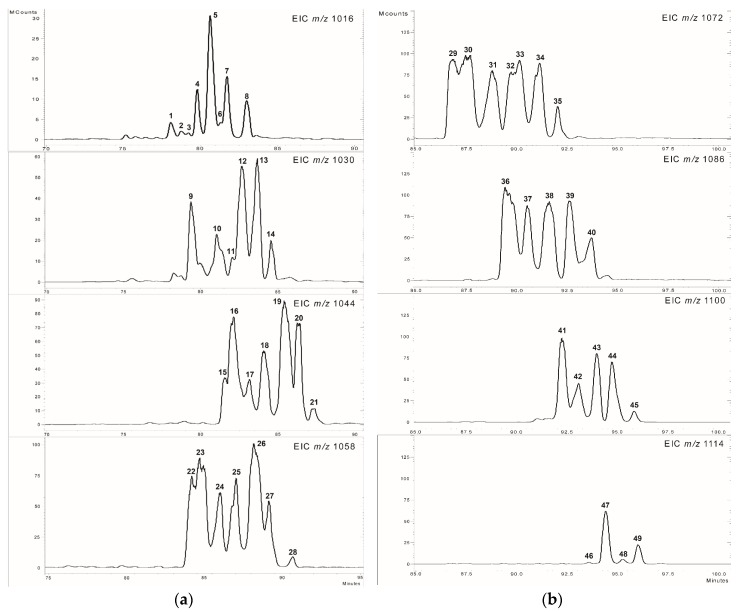
The extracted ion chromatograms of *m*/*z* 1016–1058 (**a**) and *m*/*z* 1072–1114 (**b**).

The starting point for the separation of lipopeptides was our previous study, where isocratic separations were applied [6] based on the method described by Akpa et al. [17]. Within the isocratic elution of the water/acetonitrile mixture (20:80, *v*/*v*), the surfactins with identical molecular weights eluted in well-defined time windows according to their polarities and increased molecular masses in the following order: *m*/*z* 1016, 15–27 min; *m*/*z* 1030, 16–36 min; 1044 *m*/*z*, 23–44 min; 1058 *m*/*z*, 31–55 min. This method allowed the detection of surfactins up to *m/z* 1058, but the last lipopeptide (C16-[Val7]) already eluted near to the end of the isocratic chromatographic run, suggesting the presence of other surfactin molecules with higher molecular weight [6]. The previously separated and identified isoforms represented three types of known surfactin variants and a fourth, previously unknown, group, which was characterised by the replacement of the leucine residue by valine in the amino acid position 2. Therefore, it was assumed that the prolonged separation is able to lead to gather mass spectrometric information about the possible novel surfactin variants eluted later. The introduction of the gradient separation resulted in a complete elution of the whole surfactins according to the chromatograms (Figure 2). The extracted ion chromatograms of 1016–1058 *m*/*z* values show the previously discussed surfactins (Figure 2a), and also, new peaks appeared with high intensities within the range of *m*/*z* 1072–1114 (Figure 2b), showing a 14 Da mass difference from the formerly described molecules and from each other.

The peaks on Figure 2a cover the earlier reported surfactin varieties [6]. According to these, the **1**, **3**–**7**, **11**–**14**, **18**–**21**, **25**–**28**, and **34**–**35** correspond to the C13-, C14-, C15-, and C16-[Val7], while the **2** to the C13 -[Val2]; the **8** to the C14-[Val2,7]; and finally the **9**–**10**, **15**–**17**, **22**–**24**, **30**, and **37** mark the C13-, C14-, C15-, C16-, and C17-[Sur] surfactin molecules, respectively (Supplementary Materials Figures S1–S49). These variants were already described and characterized in detail based on their MS analysis and MS^2^ patterns [6]; thus, our study focused on the newly detected forms that appeared in the higher *m*/*z* range and eluted at the end of chromatographic run.

The 21 molecules that appeared in the *m/z* range of 1072–1114 (**29**–**49**) have not been characterized in our previous work using isocratic separations, thus their identification is needed using MS^2^ fragmentations (Figure 2b, Table 2). The mass deviations following the sequence of known surfactin *m*/*z* values lead to the implication that these compounds may be the formerly characterized surfactin isoforms containing longer fatty acid chains, possibly increasing even to a length of 21 carbon atoms in the *m*/*z* value of 1114 (C21-[Val2,7]). In order to test this hypothesis, the MS^2^ analyses of the 21 possible surfactin components were conducted, whose *m*/*z* values were in the higher mass range (*m*/*z* 1072–1114). It is important to consider that surfactins possessing 1128 *m*/*z* value were not detectable at all using the MS mode of analysis and the absence of this *m*/*z* value was also confirmed later by the MS^2^ measurements.

### 2.2. Analysis of the MS^2^ Spectra of Surfactins Over m/z 1058

**Figure 3:**
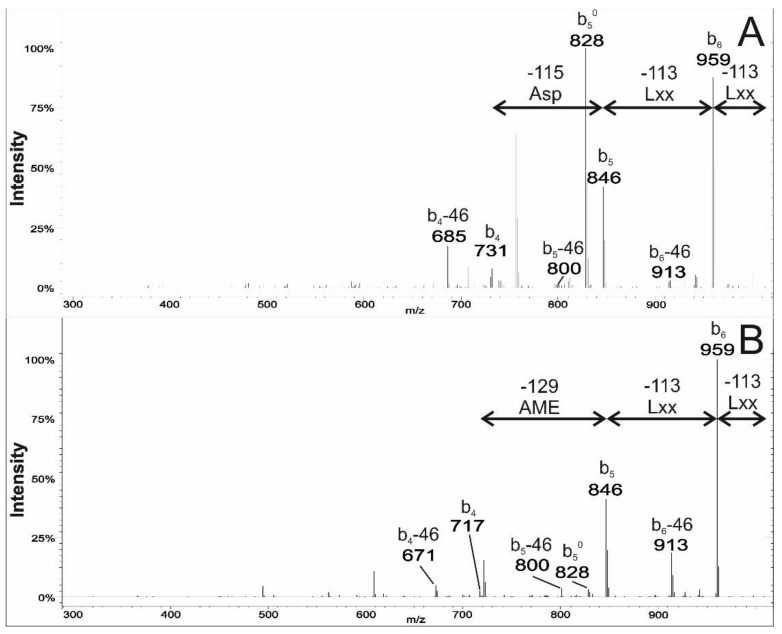
The characteristic fragment ions of C16-[Sur] (*m*/*z* 1072, **30**; (**A**)) and C15-[AME5] (*m*/*z* 1072, **29**, (**B**)) resulting via simple cleavage mechanism. AME means the aspartic acid 4-methyl ester residue.

The results of the MS^2^ analyses are demonstrated on the spectrum of the first eluted peak (**29**) of the extracted ion chromatogram with the *m/z* value of 1072 (Figure 3), while the MS^2^ spectra of the other examined components are in the Supplementary Material (Figure S1–S49). Based on the MS^2^ investigations, all the above mentioned 21 components are proven to be members of the surfactin group of the lipopeptide family. The MS^2^ spectra, originating from the fragmentation of sodiated surfactin molecules, could generally show three types of characteristic fragment ion series, which were characterized in detail in our previous study [6]. Briefly, the fragments appearing on the MS^2^ spectra recorded by the ion trap analyser could have resulted from the simple cleavage [18], internal fragmentation [6], and from McLafferty rearrangement [19] mechanisms.

In the first case, the series of fragment ions are formed when the lactone bridge connecting the C-terminal amino acid to the hydroxyl group of the fatty acid is opened by heterolytic cleavage of the bond between the sodiated oxygen atom and the carbonyl carbon atom. Then, successive eliminations of C-terminal amino acid residues produce a series of *N*-terminal fragments named b_6_, b_5_, and b_4_ [2,6]. Furthermore, the appearance of the b_5_^0^ ions were also observed, originating from the loss of water from the carboxyl group linked to the side chain of the acidic amino acid in the sequence, which commonly occurs in the case of *N*-terminal Glu residue [20]. It is important to consider that these fragments also contain the whole fatty acid part of the molecules besides the peptide sequence fragment. With the decrease in the length of the peptide chain, the intensities of these b series daughter ions were also reduced, such as the intensities of b^4^ and b^3^-type ions ranging only from 1–25% and 0–3%, respectively. The mass differences between these ions led to the identification of at least the three ending residues of the lipopeptides, including AA7, AA6, and AA5, but in certain cases, the AA4 was also able to characterize (Table 1). Furthermore, a characteristic neutral loss was also detected within the series of the simple cleavage ions on the MS^2^ spectra of each components, with the same mass differences (46 Da) from b_6_, b_5_, and b_4_ ions. This frequently appearing neutral loss could have originated either from the side chain carboxylic functions (H_2_O + CO) [21] or from the b_n_ − a_n_ (28 Da) transformation and an additional water loss (18 Da), which could be marked as a_n_^0^ transformed from the b_n_^0^ (Figure 3).

During the spectral evaluation of the surfactin components, in certain cases, extraordinary mass differences were observed between the b_5_ and b_4_ fragment ions, which indicated that there is a possibility for the replacement of the common Asp amino acid residue in the fifth position at certain surfactin variants. This observation led to the detection of the monomethyl ester surfactins containing AME instead of Asp in the fifth position of the peptide chain according to the detected mass difference between the b-series ions (Figure 3) [9]. This difference of the fragmentation patterns could be compared with each other between the [Sur] and the [AME5] type surfactins possessing same molecular weight (Figure 3). On the MS^2^ spectra resulting from the fragmentation of *m*/*z* 1072, it is obvious that the variances in the molecules are in the peptide sequences at the b_4_ fragment ions, which is *m*/*z* 731 (b_5_-115 Da) and *m*/*z* 717 (b_5_-129 Da) for [Sur] and [AME5], respectively. This observation also led to the discovery of a novel type of components within the surfactin lipopeptide family containing also AME instead of Asp in the fifth position, as well as Lxx in the fourth position ([Lxx4,AME5]) or Val in the seventh position ([AME5,Val7]). To the best of our knowledge, this is the first report on the existence of this type of surfactin molecules.

**Figure 4:**
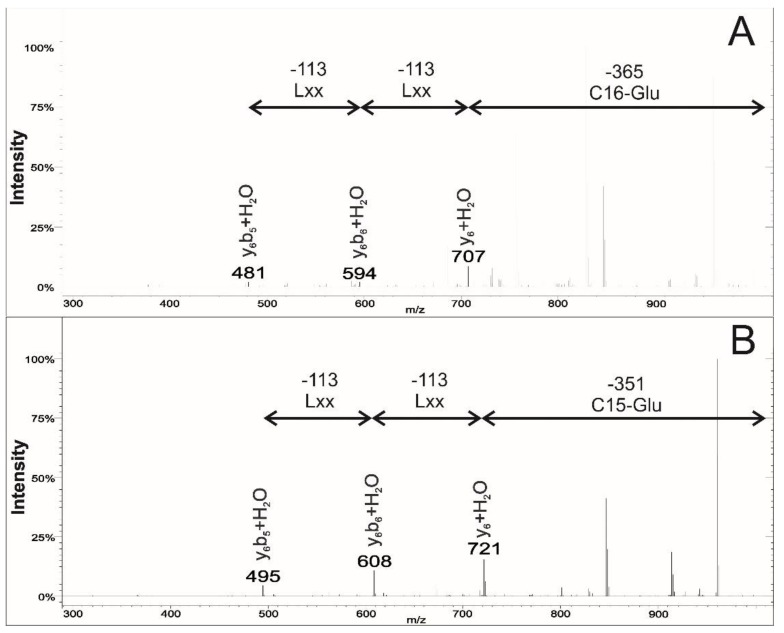
The characteristic fragment ions of C16-[Sur] (*m*/*z* 1072, **30**; (**A**)) and C15-[AME5] (*m*/*z* 1072, **29**, (**B**)) resulted via internal fragmentation mechanism.

The second series of fragments appeared at smaller *m*/*z* values, because they do not contain the fatty acid chain part of the molecule as well as the *N*-terminal Glu residue, only the internal parts of the heptapeptide of the surfactin molecules (Figure 4, Table 2). These fragments were formed via the double hydrogen transfer mechanism, which suggest the opening of the ring at the ester side causing the additional water increase on the y_6_ and y_6_b_m_ (m ≤ 6) ions [6,22]. The major fragment of this group is represented by the y_6_ + H_2_O and the following members derived from this major fragment ion by the same consecutive elimination of C-terminal amino acids, as in the case of the simple cleavage described above, thus represented as y_6_b_6_ + H_2_O and y_6_b_5_ + H_2_O [6].

Examining the *m*/*z* values of the internal fragment ions, two fragments are additionally important for the confirmation of the existence of the AME in the fifth position of the peptide sequence. The first is the *m*/*z* value of the y_6_ + H_2_O fragment representing sum mass of the last six C-terminal amino acids, while the second is the mass difference of this fragment ion from the precursor ion (sodiated adduct) resulting in the length of the linked fatty acid chains. This characteristic fragment ion and 14 Da deviation between the surfactins containing Asp_5_ or AME_5_ could be compared on their spectra (Figure 4). In the case of Figure 4B, the 14 Da mass increase of y_6_ + H_2_O suggests that the fatty acid chain of **29** molecule consists of 15 carbon atoms, while the **30** contain C-16 fatty acid residue (Figure 4). Therefore, the knowledge of the two C-terminal amino acids from the simple cleavage fragmentation pattern (both Lxx for **29** and **30**) and the peculiar shift in the mass of y_6_b_4_ + H_2_O fragment ions could have only originated from the deviation in the amino acid sequence between the second and the fifth (AA_2_–AA_5_) positions of the sequence. On the other hand, a major confirmatory argument would also be the presence of y_6_b_4_ + H_2_O fragment ions, but it was not visible on the MS_2_ spectra recorded with the applied ion trap mass spectrometric analyzer. However, it could be concluded that the examination of the presence of b_4_ and y_6_ + H_2_O fragment ions on the MS^2^ spectra of surfactins, as well as their relationship with the sodium adduct precursor ion masses, comprehensively determine the alteration of the Asp to AME in the AA_5_ position in this type of surfactins.

The third series of fragments resulted from intraresidual McLafferty rearrangement, where the hydrogen at the γ-carbon atom from the *N*-terminal Glu residue transfers to its carbonyl oxygen causing the cleavage of the link inside the amino residue between the αC–βC atoms. These rearrangements could usually be measured related to the surfactins as a mass difference of 72 Da from both the sodiated precursor ion and the b_5_^0^ on the product ion spectra [6]. These neutral losses were detected in all cases in the lower *m*/*z* region up to *m*/*z* 1030, while it completely missed in the highest *m/z* region ranged from *m*/*z* 1086 (Table 2, Figure S1–S49). Thus, it seems to be that the presence or absence of the neutral loss originating from the McLafferty rearrangement depends mainly on the length of the linked fatty acid chain.

Using the above-mentioned rules, the MS^2^ spectra of each suspected surfactin molecule were also analysed. Each surfactin compound in the higher mass range (*m*/*z* > 1058) was identified based on the MS^2^ spectra of all detected peaks of the extracted ion chromatograms and the mentioned considerations. Altogether, three types of surfactins were recognized containing AME at the fifth position including [AME5], [Lxx4, AME5], and [AME5, Val7]; neither the GME monomethyl isoforms nor the dimethyl isomorfs were detected. The characteristic ions of the simple cleavages and the internal fragments, as well as the McLafferty rearrangement related to the different isoforms, are shown in Table 2 and their MS_2_ spectra are in the Supplementary Material (Figure S1–S49). The surfactins with lower masses (*m*/*z* < 1072) produced by the *B. subtilis* SZMC 6179J strain, as well as their structural elucidations, were published in our previous report [6]. The MS^2^ fragmentation of 1128 *m*/*z* value was also carried out in a separated chromatographic run, but no fragments were detected supporting the absence of this surfactin variant.

### 2.3. Characterization of the Amount of Produced Surfactin 

Observing the results provided by the MS^2^ analyses of the sodiated surfactin molecules led us to the conclusion that the examined *B. subtilis* strain produced relatively high amounts of the different isoforms in total. In certain cases, an accurate gas chromatographic method could be applied for the quantitation of surfactins. This approach was capable of simultaneously measuring both the derivatized fatty acid chain and amino acids after the hydrolysis of the surfactins [23]. However, because of the high complexity of our sample, the relative quantities of the variants found in the sample extract were expressed as percentages of the total surfactin content based on the areas of their sodiated aducts ([M + Na]^+^) estimating their relative amounts semi-quantitatively. The ionization efficiency in the used ion source may vary along the different variants, but it could provide useful information about the approximate proportions of their amounts, as most of the variants have not yet been isolated in pure form and the analytical reference standards are not available.

**Figure 5:**
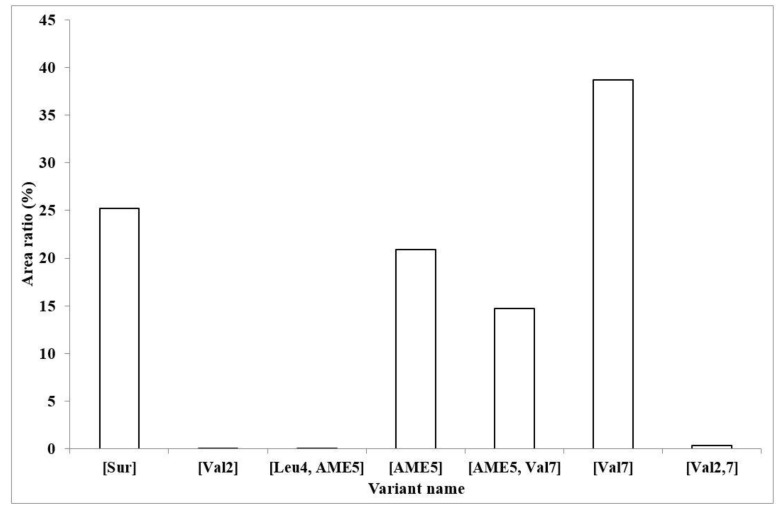
The ratios of all the produced surfactin isoforms in our sample. The percentage values are based on the integrated areas of the particular peaks of each isoform in the extracted ion chromatograms.

To compare the relative quantity of the isoforms containing an AME residue with the other variants previously found and characterized from samples of strain SZMC 6179J, the ratio of the integrated areas of sodiated molecular ions in full scan MS mode of both the previously reported lower *m*/*z* region and the ones described in the present work bearing higher *m*/*z* values were represented in a diagram (Figure 5). The presence of these monomethyl isoforms is not negligible by any means, altogether having an area ratio of 36%; this sum exceeds the amount of the first discovered surfactin molecule ([Sur], 25%), which is considered to be the most common variant. The appearance of Val in the seventh position is the most dominant, 54% of the compounds possess this change in the amino acid sequence, while the presence of Val in the second and Leu in the fourth position occurs in less than 1% in both cases.

**Figure 6:**
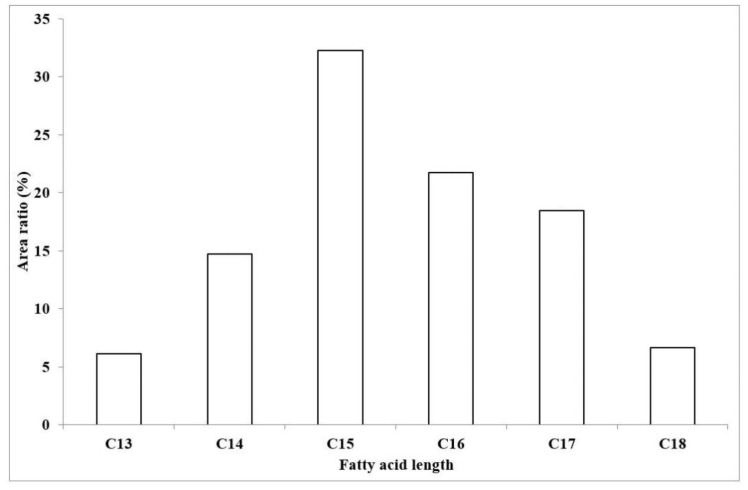
The ratios of the produced surfactin molecules possessing different fatty acid chain lengths based on the integrated areas of the peaks in the extracted ion chromatograms.

To examine the ratios of the different fatty acid chain lengths of the produced compounds, their areas were also compared (Figure 6). This excludes the previously suggested possibility of an isoform having a 21 carbon atom long chain in our sample, and also shows the longest fatty acid chain as C18 in about 7% of the total amount of surfactin, extending the carbon chain length suggested by Bonmatin et al. [2] to 15. Although Bóka et al. [6] found the C16-[Val7] variant of surfactin in a mere 3% relative amount, the introduction of our modified gradient program enabling the separation of component ions of higher masses increased the ratio of C16 compounds to 22%, not to mention the fact that 47% of the surfactin molecules found in our sample bears an aliphatic chain length of 16 or more carbon atoms.

**Figure 7:**
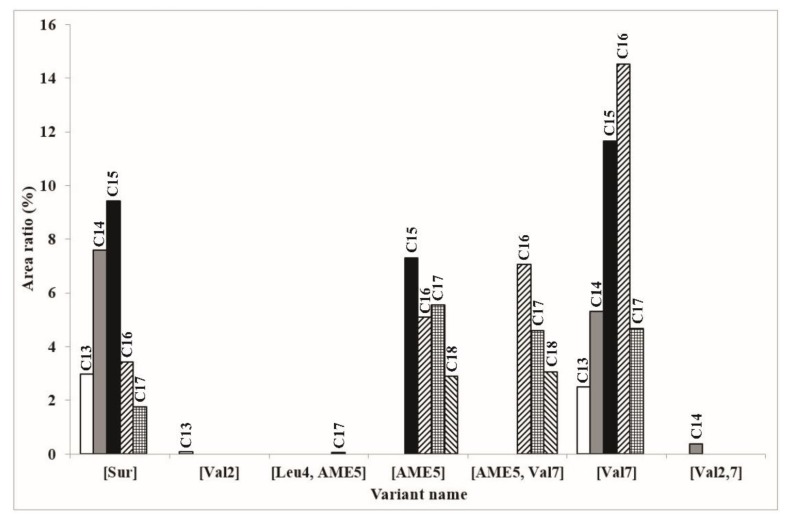
The summarizing diagram of all the different surfactin molecules and their homologues found in *B. subtilis* SZMC 6179J.

For the full perception of the spectrum of the surfactin molecules found in our sample, a summarizing diagram was prepared (Figure 7). It reveals the reason why the [AME] variants have rarely been found before, as their fatty acid chain lengths range from C15 to C18, which means that their sodiated ion masses are 1072–1114 Da, respectively. The chromatographic parameters applied before did not allow the separation of these fractions, which also explains the low relative amount of the C16-[Val7] isoform found by Bóka et al. [6], although according to our recent examinations, this variant is produced in the highest amount by *B. subtilis* SZMC 6179J. The surfactin molecules being the most diverse regarding the aliphatic chain lengths are the [Sur] and the [Val7] variants, both with five different homologues ranging from C13 to C17, while only the ones containing an AME residue in their fifth amino acid positions ([AME5] and [AME5, Val7]) have the C18 homologue.

## 3. Materials and Methods 

### 3.1. Chemicals and Reagents

All solvents and reagents were analytical or the highest grade available. Hydrochloric acid, methanol, and acetonitrile were purchased from VWR International (Budapest, Hungary). Trifluoroacetic acid (TFA) was purchased from Sigma Aldrich (Budapest, Hungary). HPLC grade water with a resistivity of 18 MΩ was produced by ultrafiltration with a Millipore Milli-Q Gradient A10 water purification system (Merck, Budapest, Hungary).

### 3.2. Microorganism and Culture Conditions

The examined *Bacillus subtilis* strain SZMC 6179J was previously isolated from tomato rhizosphere and its antagonistic properties were reported and characterized by Vágvölgyi et al. [24]. The strain was stored in the Szeged Microbiology Collection (SZMC, www.szmc.hu) for a long period, and for the daily usage, it was maintained on nutrient agar (5 g/L peptone, 3 g/L yeast extract, 5 g/L NaCl, 15 g/L agar) slants and stored at 4 °C.

For the surfactin production, a liquid ferment broth was applied according to Besson et al. [25] containing 10 g/L glucose, 5 g/L glutamic acid, 1 g/L KH_2_PO_4_, 1 g/L K_2_HPO_4_, 1 g/L KCl, 500 mg/L MgSO_4_ × 7 H_2_O, 5 mg/L FeSO_4_ × 7 H_2_O, and 160 µg/L CuSO_4_ × 5 H_2_O. Bacteria (5 × 10^7^ cells) were inoculated into 50 mL medium in 250 mL Erlenmeyer flasks followed by the incubation on a rotary shaker at 120 rpm for five days at 25 °C.

### 3.3. Extraction of Surfactins

The bacterial cells were separated from the ferment broths via centrifugation at 8000 *g* for 10 min. The pH of the supernatant was decreased to 2 with HCl and the lipopeptides were precipitated overnight at 4 °C. The pellets were collected by centrifugation (10,000 *g*, 10 min) and resolved in 3 mL methanol [6].

### 3.4. Liquid Chromatography–Mass Spectrometry Analysis

Surfactin homologues extracted from the *B. subtilis* strain SZMC 6179J were identified by HPLC-ESI-IT-MS. Gradient RP-HPLC elution was carried out on a Phenomenex Prodigy analytical column (100 × 2.0 mm, 3 µm; Gen-Lab, Budapest, Hungary) with an Agilent 1100 Series HPLC system (Palo Alto, CA, USA) equipped with a binary pump, a vacuum degasser, and a µWell-plate autosampler. The columns were thermostated at 35 °C using a model 7990 Space column heater (Jones Chromatography, Hengoed, UK). The gradient solvent-delivery system consisted of two solvents, A was H_2_O and B was a mixture of acetonitrile/methanol (1:1, *V*/*V* %), both solvents supplemented with 0.05% TFA. The gradient elution was applied at a flow rate of 250 µL/min and was started with 47% of eluent B for 12 min and increased linearly to 95% at 92 min, where value was held for 16 min and then decreased to the initial 15% in two minutes, and remained constant until the pressure stabilized ending the run of 120 min in total. The injection volume was 3 µL.

The MS and MS^2^ analyses were performed on a 500-MS ion trap mass spectrometer (Agilent, Palo Alto, CA, USA) equipped with an ESI source in positive mode based on our previous study [6]. The utilized ESI parameters were as follows: spray chamber temperature, 50 °C; drying gas (N_2_) pressure and temperature, 30 psi and 350 °C, respectively; nebulizer gas (N_2_) pressure, 50 psi; needle voltage, 5000 V; spray shield voltage, 600 V. The general parameters were as follows: maximum scan times, 1.51 s/scan; scans averaged, 2 µscans; data rate, 0.66 Hz; multiplier offset, 0 V. Ionization control parameters were as follows: target TIC, 100%; max ion time, 250,000 µseconds. The full scan parameters were as follows: capillary voltage, 231.3 V; RF loading, 68%; low mass, 100 *m*/*z*; high mass, 2000 *m*/*z*. 

The MS^2^ measurements on the sodium adducts of the molecules were also achieved using the same ESI source and general MS parameters mentioned above. In the case of MS^2^ measurements, for the 1016, 1030, 1044, 1058, 1072, 1086, 1100, 1114, and 1128 *m*/*z* values, the following excitation storage level (*m*/*z*) /excitation amplitude values were applied: (V) 273.5/3.91, 277.4/9.96, 281.3/4.02, 285.3/4.07, 289.3/4.12, 293.3/4.17, 297.4/4.22, 301.5/4.27, and 305.6/4.32, respectively. Each *m/z* value of the sodium adducts was monitored alone in separated chromatographic runs to avoid the possible cross-talk effects in the mass analyser. 

### 3.5. Nomenclature

Surfactin isoforms were designated according to Grangemard et al. [13] and Bóka et al. [6]. Briefly, the first discovered surfactin sequence (Glu-Leu-Leu-Val-Asp-Leu-Leu) was denoted as [Sur] and any changes in the peptide sequence were indicated with the abbreviation and position of the altered amino acid, for example, [Val2], [Val7], and [Val2,7]. The esterified form of aspartic acid and glutamic acid at the side chain carboxyl group were abbreviated as AME and GME, respectively. As the applied mass spectrometric technique could not distinguish between the Leu and Ile isobaric residues, this sequence element was marked as Lxx in this paper. The amino acid residues present in the sequences of the surfactins are designated in general by AA^n^, the superscript ‘n’ indicating the position number of each amino acid from the *N*-terminal end of the peptide chain. Furthermore, the fragment ions on the MS^2^ spectra were designated according to the terminology published by Roepstorff and Fohlman [26], as well as Biemann [27], while the internal fragments of sodiated fragment ions were designated by the y_n_b_m_ nomenclature [6,22].

## 4. Conclusions

We developed an efficient HPLC gradient elution to effectively separate the fractions of surfactin produced by *B. subtilis* in the higher range of *m/z* values. When the structures of these compounds with greater masses were examined by MS^2^ analyses, isoforms of surfactin molecules containing AME in their fifth peptide position were recognized, also involving the newly described types ([Lxx4,AME5], [AME5,Val7]). After the study of the MS^2^ spectra validated our suggestions regarding the amino acid sequence, we calculated and compared the ratios of the produced surfactin isoforms. The results of these semi-quantitative comparisons proved the presence of these variants to be remarkable amount. In the case of the highest *m/z* values (at *m*/*z* 1100 and 1114), only these new isoforms possessing AME_5_ could be detected and the formation of C18-[AME5] had a yield close to 100%, thus posing the possibility of a facilitated selective separation of the novel isoform closest to the firstly discovered surfactin in structure; therefore, its biological effects can be characterized in comparison to its most extensively studied variants.

## Figures and Tables

**Table 1:** Natural surfactin peptide sequence variants including the recently found isoforms containing AME in the fifth position.

Nomenclature of Surfactin	Peptide							References
	AA_1_	AA_2_	AA_3_	AA_4_	AA_5_	AA_6_	AA_7_	
[Sur] surfactin	Glu	Leu	Leu	Val	Asp	Leu	Leu	[10]
[Val2] surfactin	Glu	Val	Lxx	Val	Asp	Lxx	Lxx	[6]
[Ala4] surfactin	Glu	Leu	Leu	Ala	Asp	Leu	Leu	[11]
[Leu4] surfactin	Glu	Leu	Leu	Leu	Asp	Leu	Leu	[12]
[Ile4] surfactin	Glu	Leu	Leu	Ile	Asp	Leu	Leu	[12]
[Ile2,4] surfactin	Glu	Ile	Leu	Ile	Asp	Leu	Leu	[13]
[Val7] surfactin	Glu	Leu	Leu	Val	Asp	Leu	Val	[14,15]
[Ile7] surfactin	Glu	Leu	Leu	Val	Asp	Leu	Ile	[14]
[Val2,7] surfactin	Glu	Val	Leu	Val	Asp	Leu	Val	[13]
[Val2, Ile7] surfactin	Glu	Val	Leu	Val	Asp	Leu	Ile	[16]
[Ile2, Val7] surfactin	Glu	Ile	Leu	Val	Asp	Leu	Val	[16]
[Ile2,4,7] surfactin	Glu	Ile	Leu	Ile	Asp	Leu	Ile	[13]
[GME1,AME5] surfactin	GME	Leu	Leu	Val	AME	Leu	Leu	[7]
[GME1,Val7] surfactin	GME	Leu	Leu	Val	Asp	Leu	Val	[7]
[GME1, Ile7] surfactin	GME	Leu	Leu	Val	Asp	Leu	Ile	[7]
[AME5] surfactin	Glu	Leu	Leu	Val	AME	Leu	Leu	[8]
[AME5,Val7] surfactin	Glu	Lxx	Lxx	Val	AME	Lxx	Val	This study
[Lxx4, AME5] surfactin	Glu	Lxx	Lxx	Lxx	AME	Lxx	Lxx	This study

GME—glutamic acid 5-methyl ester, AME—aspartic acid 4-methyl ester.

**Table 2:** Characteristic ions of the different surfactin isoforms found in the sample from the fragmentation of *m*/*z* 1072, 1086, 1100, and 1114.

Precursor Ion [M + Na]^+^ (*m*/*z*)	Peak No.	Name	Simple Cleavage				McLafferty Fragments		Internal Fragments		
			b_6_	b_5_	b_5_^0^	b_4_	[M+Na]^+^-72	b_5_^0^-72	y_6_+H_2_O	y_6_b_6_+H_2_O	y_6_b_5_+H_2_O
1072	**30**	C16-[Sur]	959	846	828	731	1000	756	707	594	481
	**34–35**	C17-[Val7]	973	860	842	745	1000	770	693	594	481
	**29, 31**	C15-[AME5]	959	846	828	717	n.d.	756	721	608	495
	**32–33**	C16-[AME5,Val7]	973	860	842	731	1000	n.d.	707	608	495
1086	**37, 39**	C17-[Sur]	973	860	842	745	1014	770	707	594	481
	**36, 38**	C16-[AME5]	973	860	842	731	n.d.	n.d.	721	608	495
	**40**	C17-[AME5,Val7]	987	874	856	745	n.d.	n.d.	707	608	495
1100	**41–43**	C17-[AME5]	987	874	856	745	n.d.	n.d.	721	608	495
	**44–45**	C18-[AME5,Val7]	1001	888	870	759	n.d.	n.d.	707	608	495
1114	**46**	C17-[Lxx4,AME5]	1001	888	870	759	n.d.	n.d.	735	622	509
	**47–49**	C18-[AME5]	1001	888	870	759	n.d.	n.d.	721	608	495

n.d.: not detected.

## Supplementary Materials

The following are available online, Figures S1–S49.
