# Supplementary material for: High-Frequency Occurrence of Surfactin Monomethyl Isoforms in the Ferment Broth of a Bacillus subtilis Strain Revealed by Ion Trap Mass Spectrometry
Source: Molecules. 2018 Sep 1;23(9):2224. doi: 10.3390/molecules23092224 (PMC6225151; doi:10.3390/molecules23092224)
Supplement: Supplementary file 1 [file molecules-23-02224-s001.pdf]

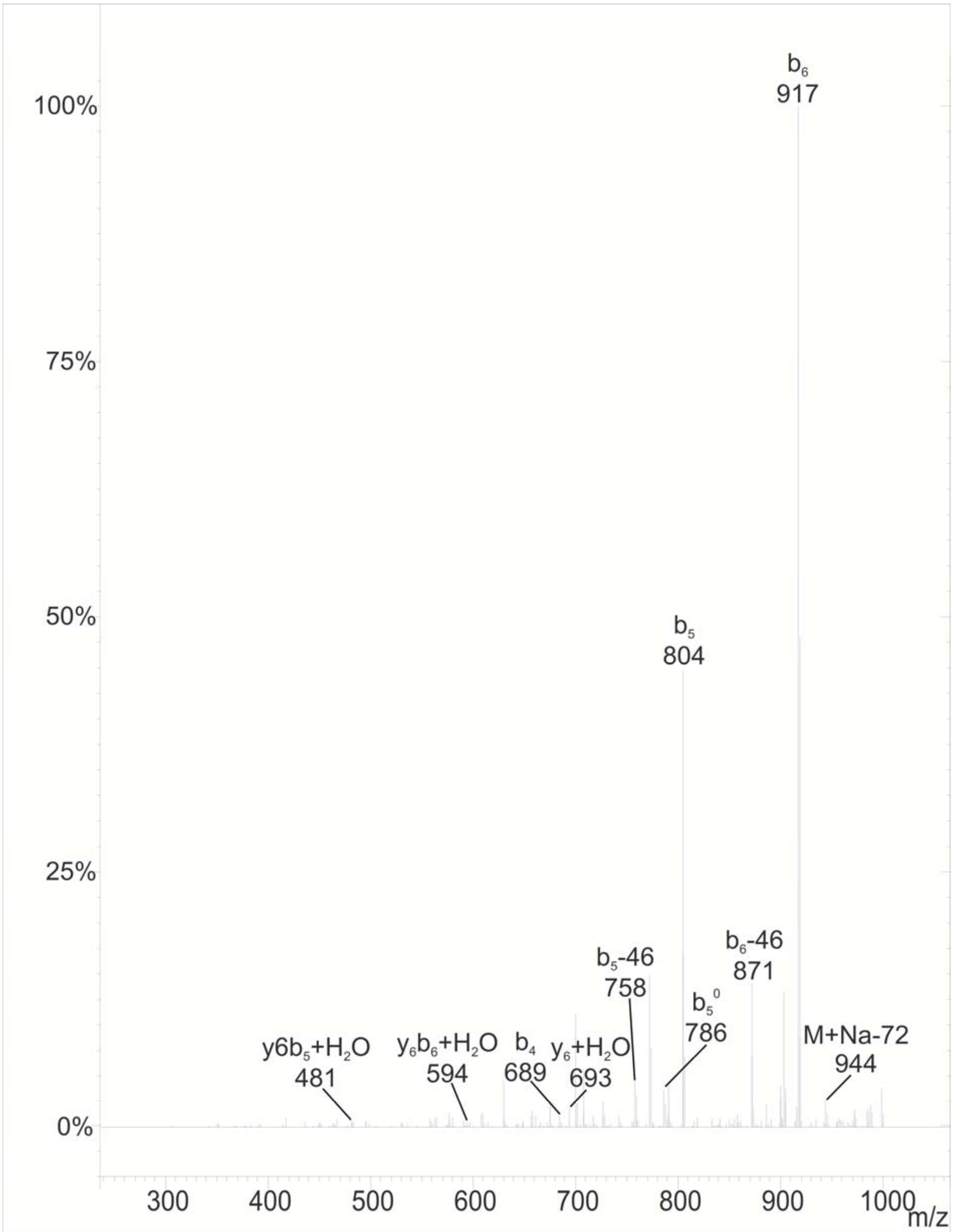


**Figure S1.** The MS[2] spectrum of **1** (C13-[Val7]) eluted at Rt = 78.08 min (*m*/*z* = 1016).

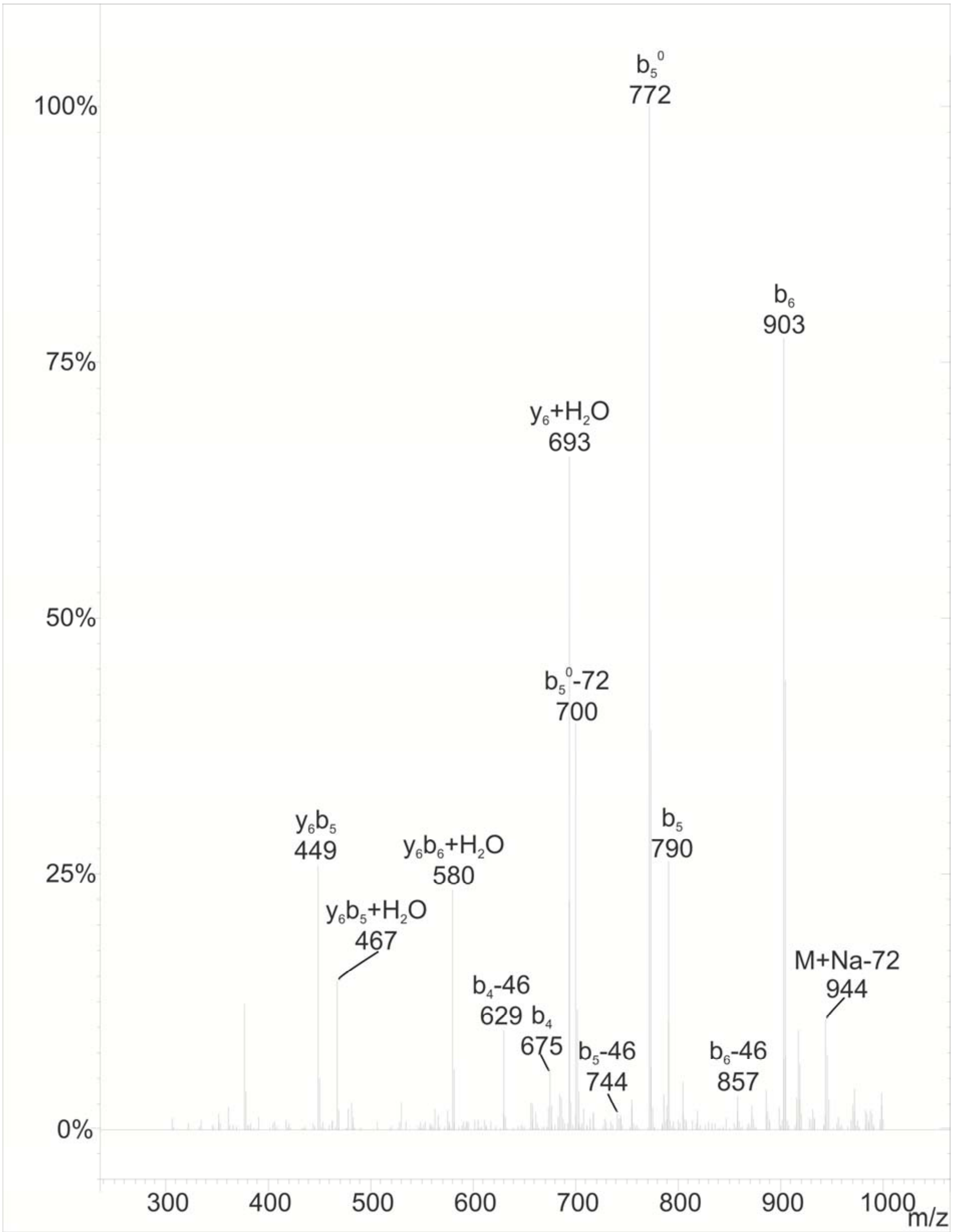


**Figure S2.** The MS[2] spectrum of **2** (C13-[Val2]) eluted at Rt = 78.74 min (*m*/*z* = 1016).

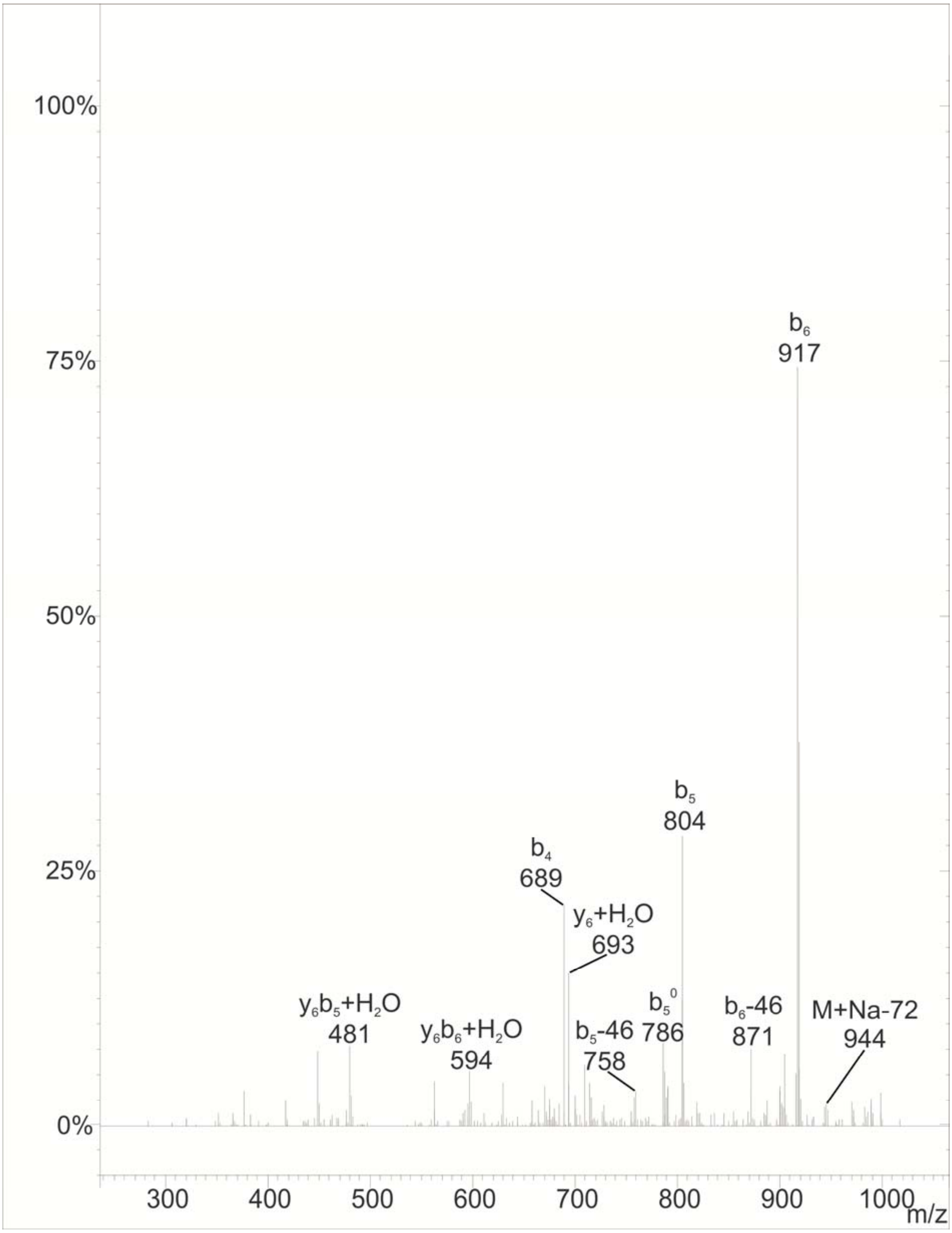


**Figure S3.** The MS² spectrum of **3** (C13-[Val7]) eluted at Rt = 79.23 min (*m*/*z* = 1016).

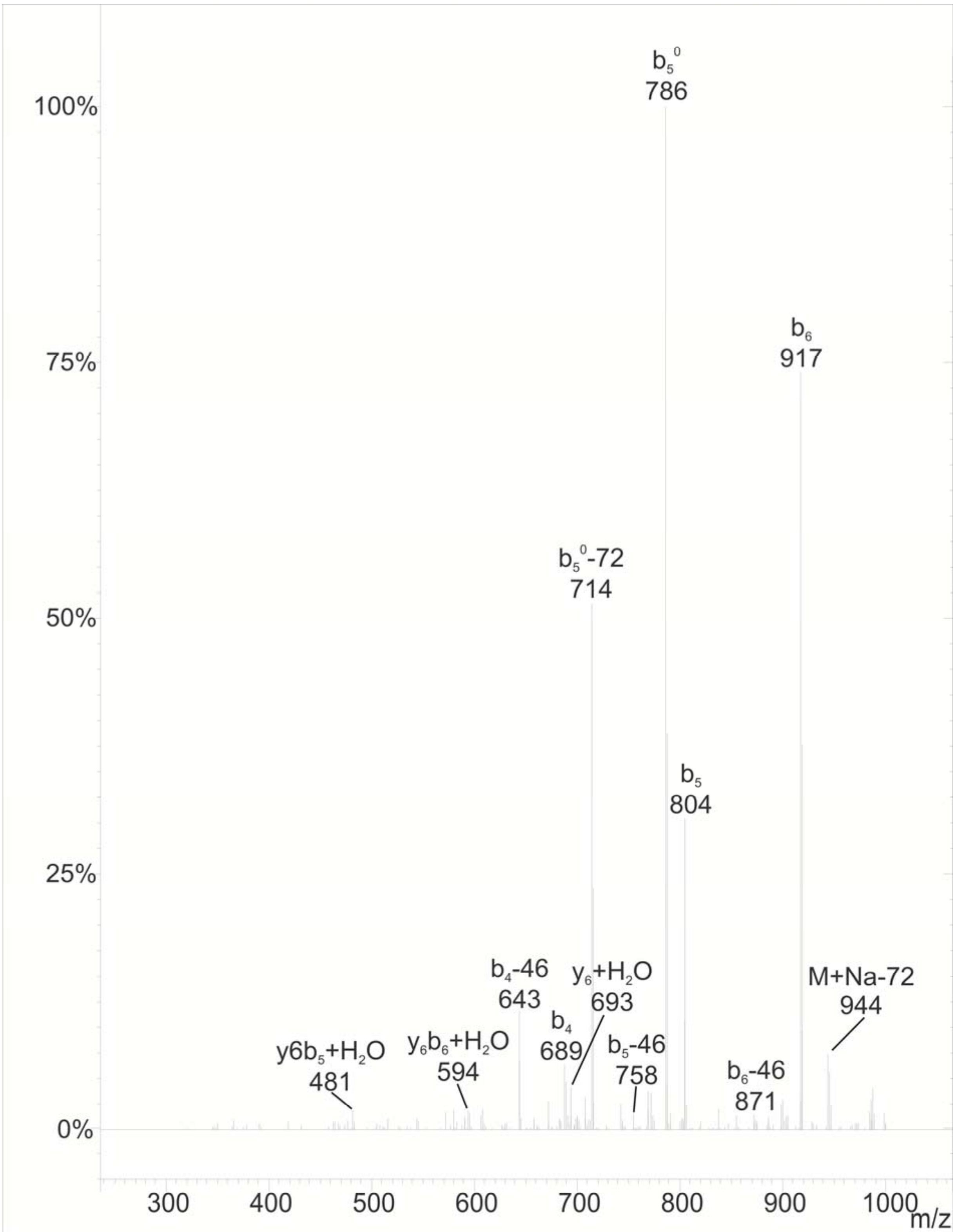


**Figure S4.** The $MS^2$ spectrum of **4** (C13-[Val7]) eluted at Rt = 79.80 min ($m/z$ = 1016).

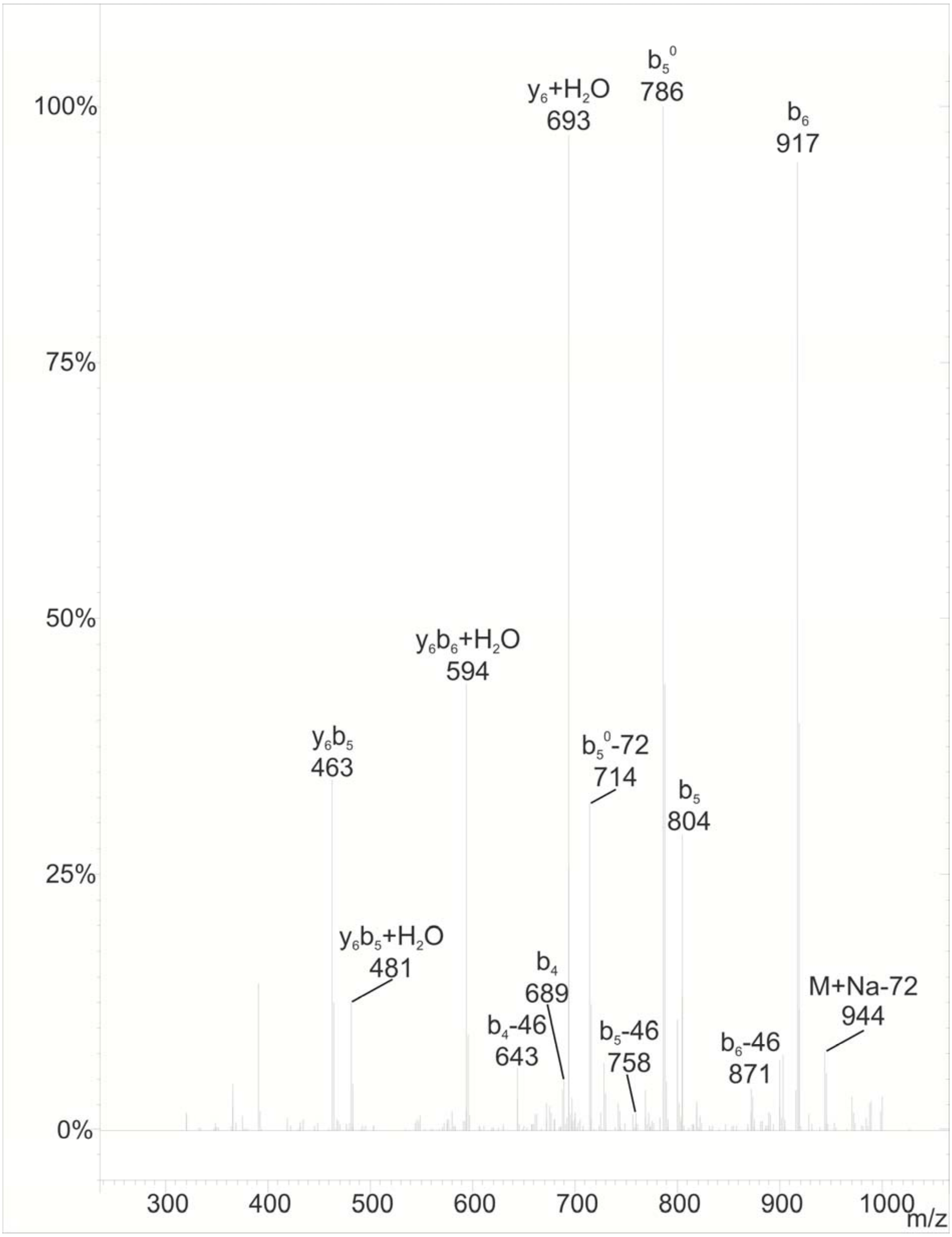


**Figure S5.** The MS[2] spectrum of **5** (C13-[Val7]) eluted at Rt = 80.63 min (*m*/*z* = 1016).

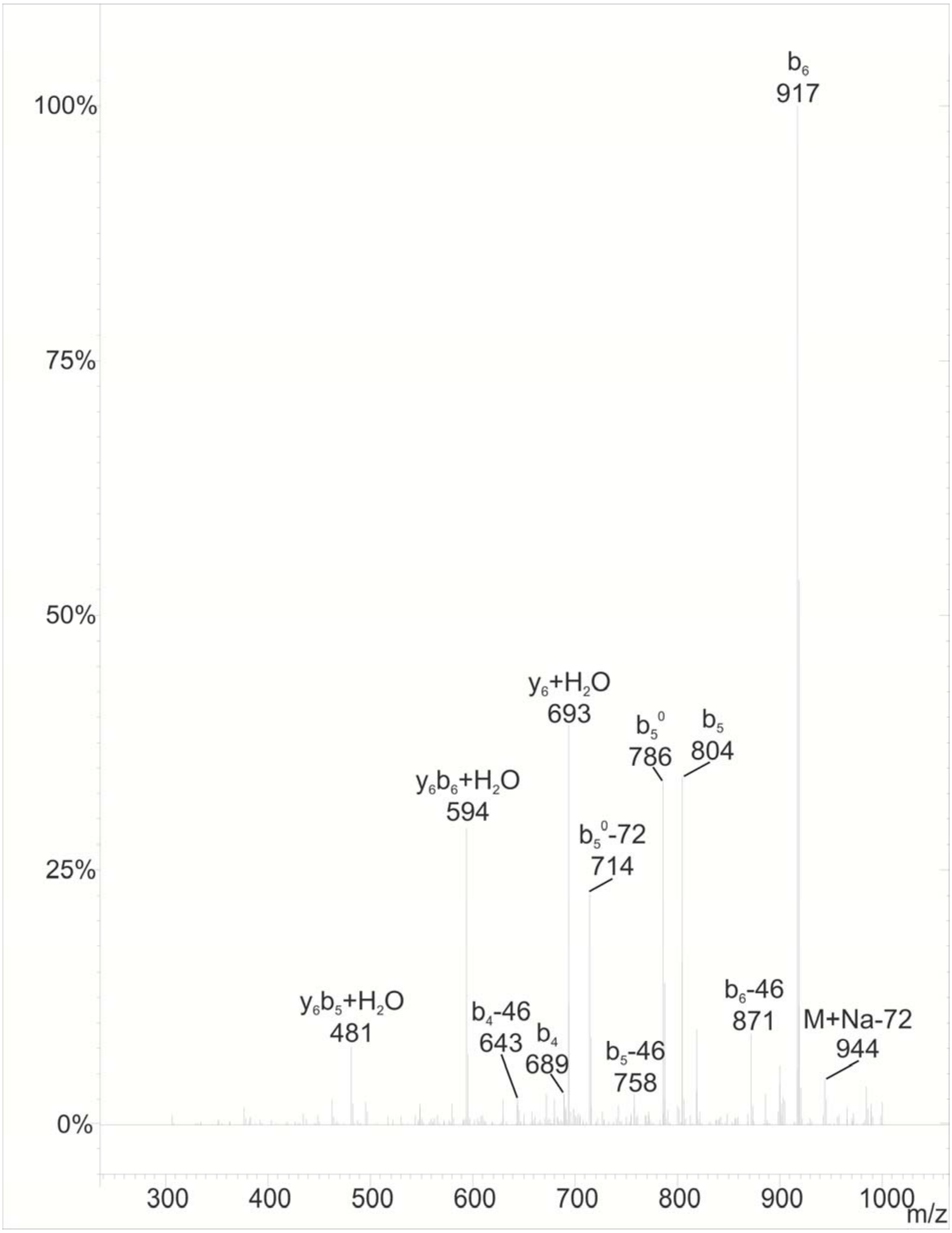


**Figure S6.** The MS$^2$ spectrum of **6** (C13-[Val7]) eluted at Rt = 81.29 min (*m*/*z* = 1016).

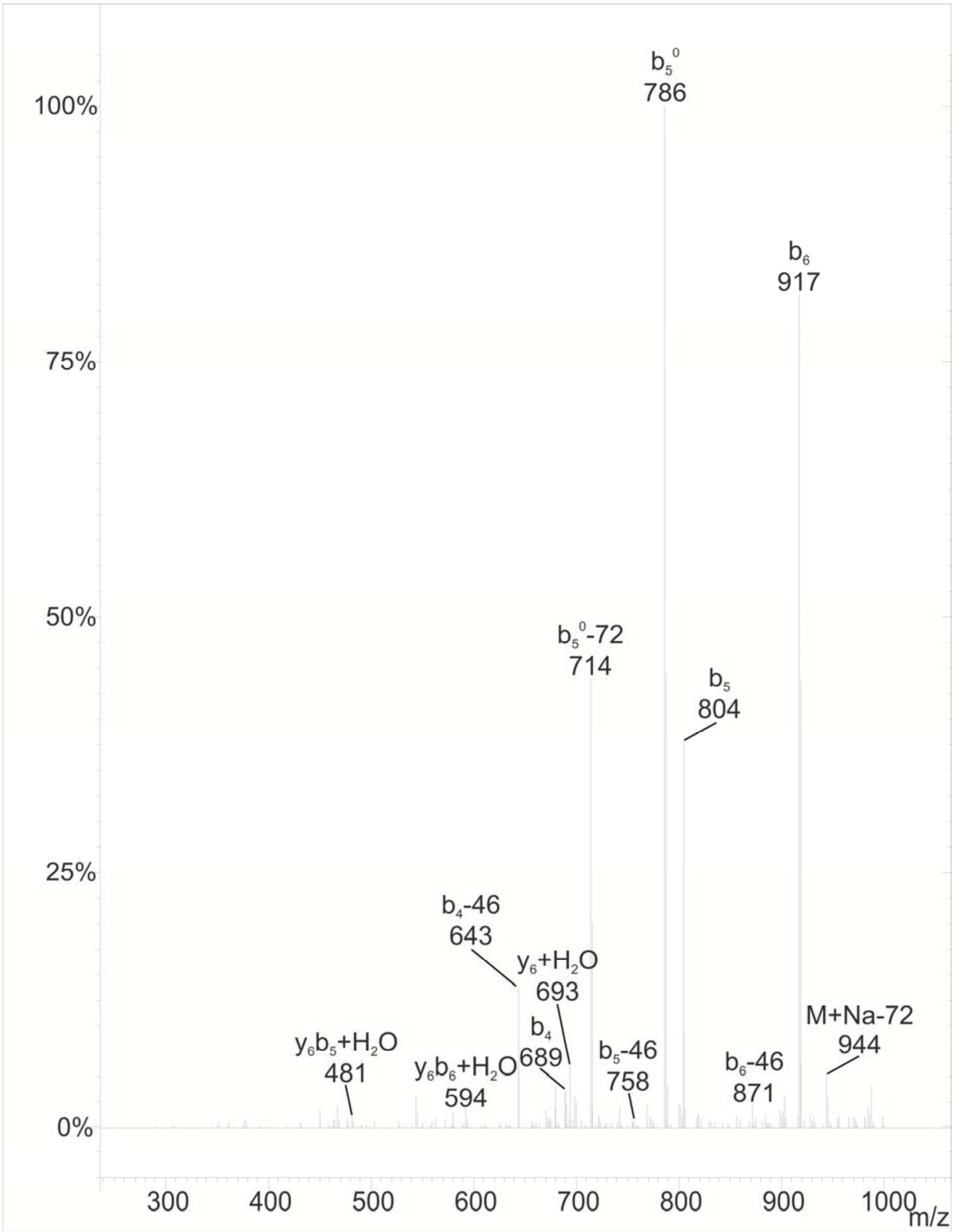


**Figure S7.** The MS[2] spectrum of **7** (C13-[Val7]) eluted at Rt = 81.73 min ($m/z$ = 1016).

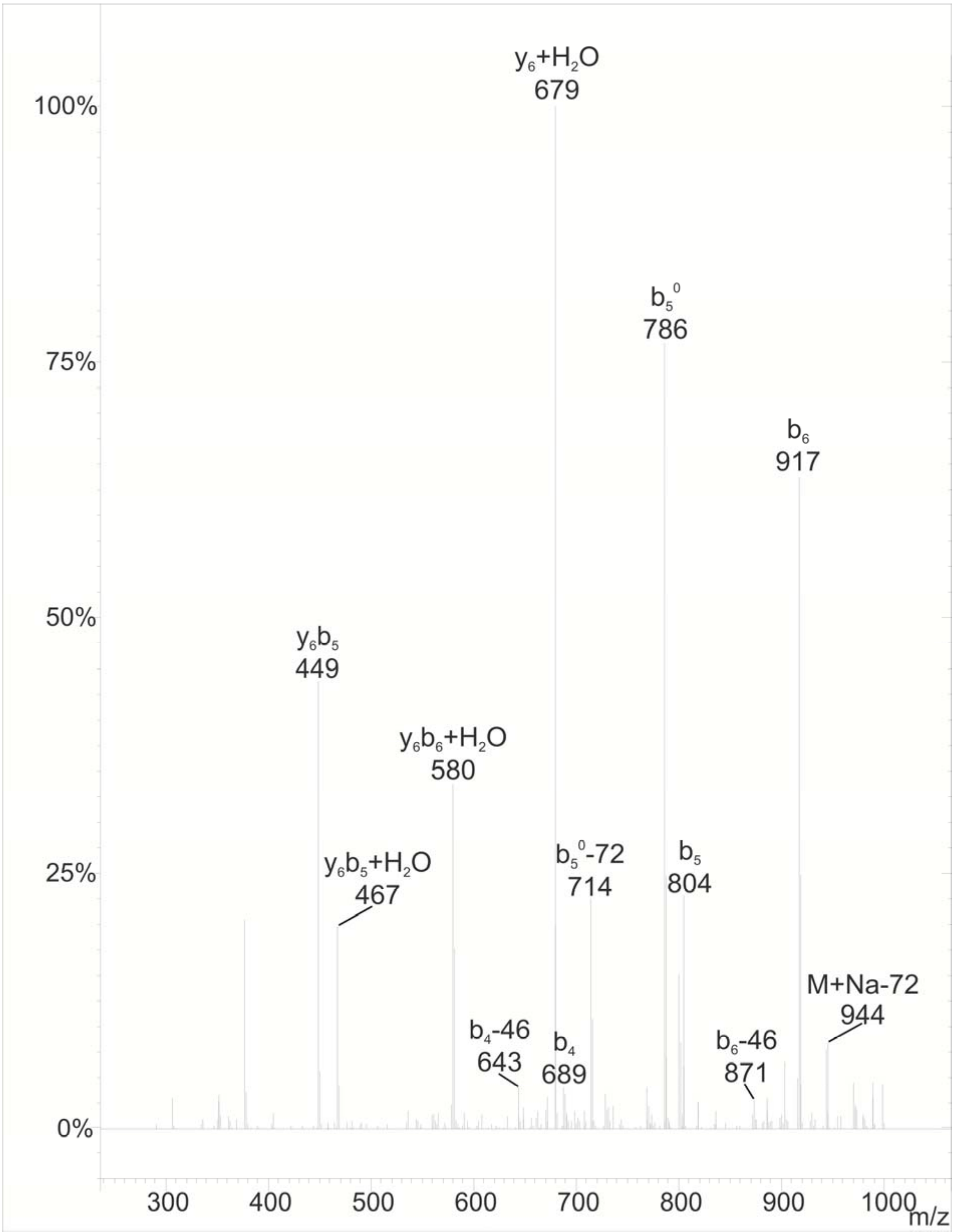


**Figure S8.** The MS[2] spectrum of **8** (C14-[Val2,7]) eluted at Rt = 82.99 min (*m*/*z* = 1016).

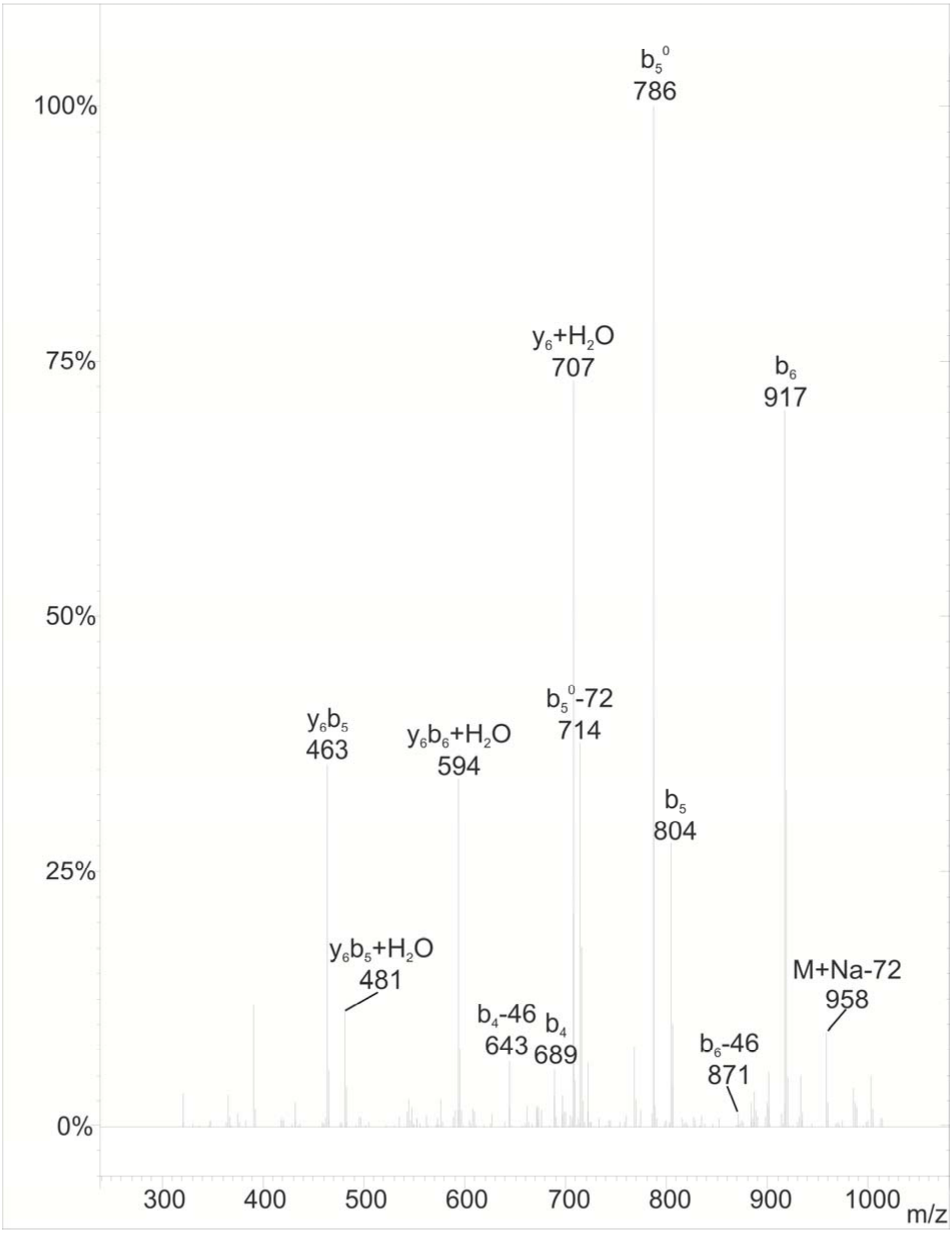


**Figure S9.** The MS[2] spectrum of **9** (C13-[Sur]) eluted at Rt = 79.37 min (*m*/*z* = 1030).

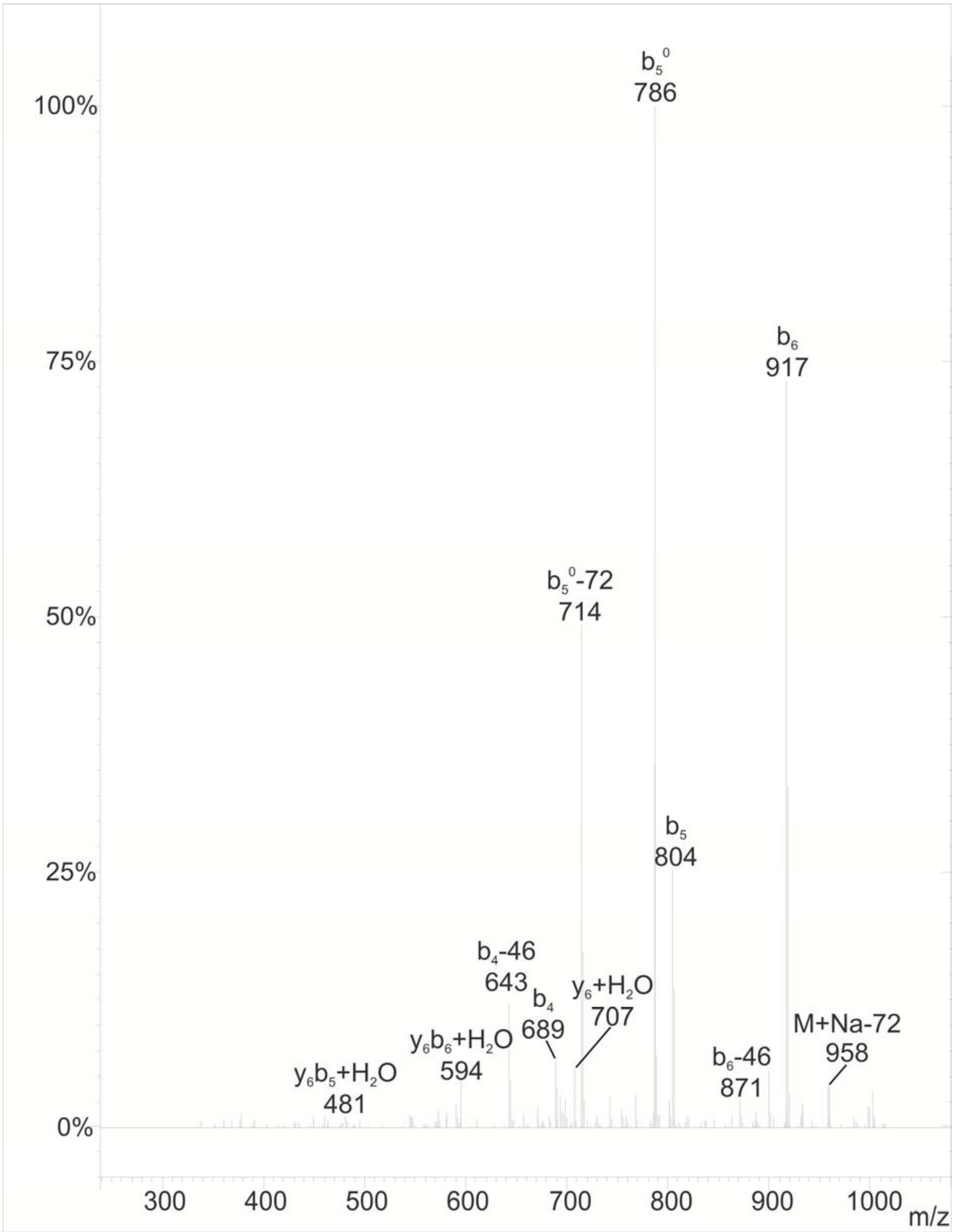


**Figure S10.** The MS[2] spectrum of **10** (C13-[Sur]) eluted at Rt = 81.03 min (*m/z* = 1030).

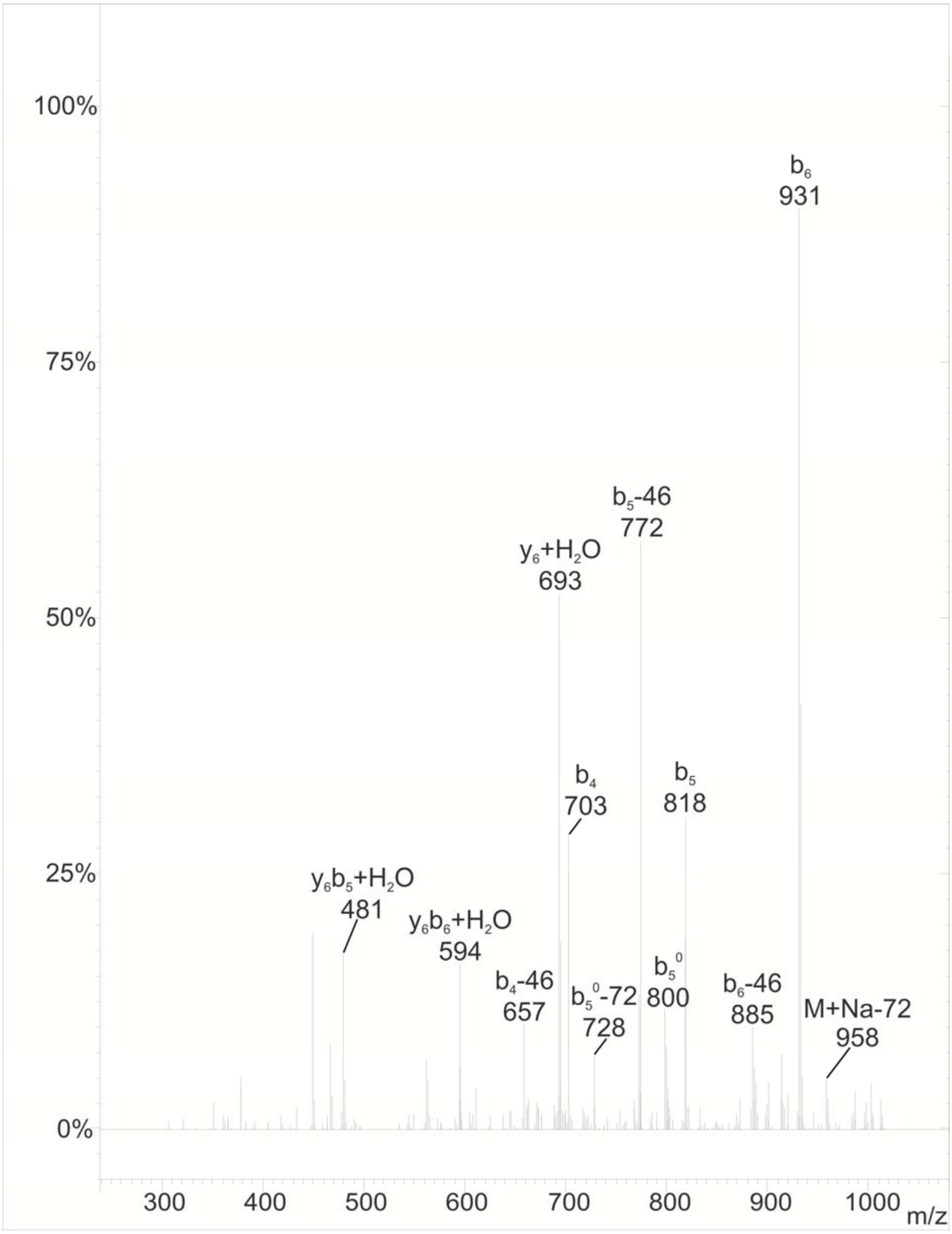


**Figure S11.** The MS[2] spectrum of **11** (C14-[Val7]) eluted at Rt = 82.03 min (*m/z* = 1030).

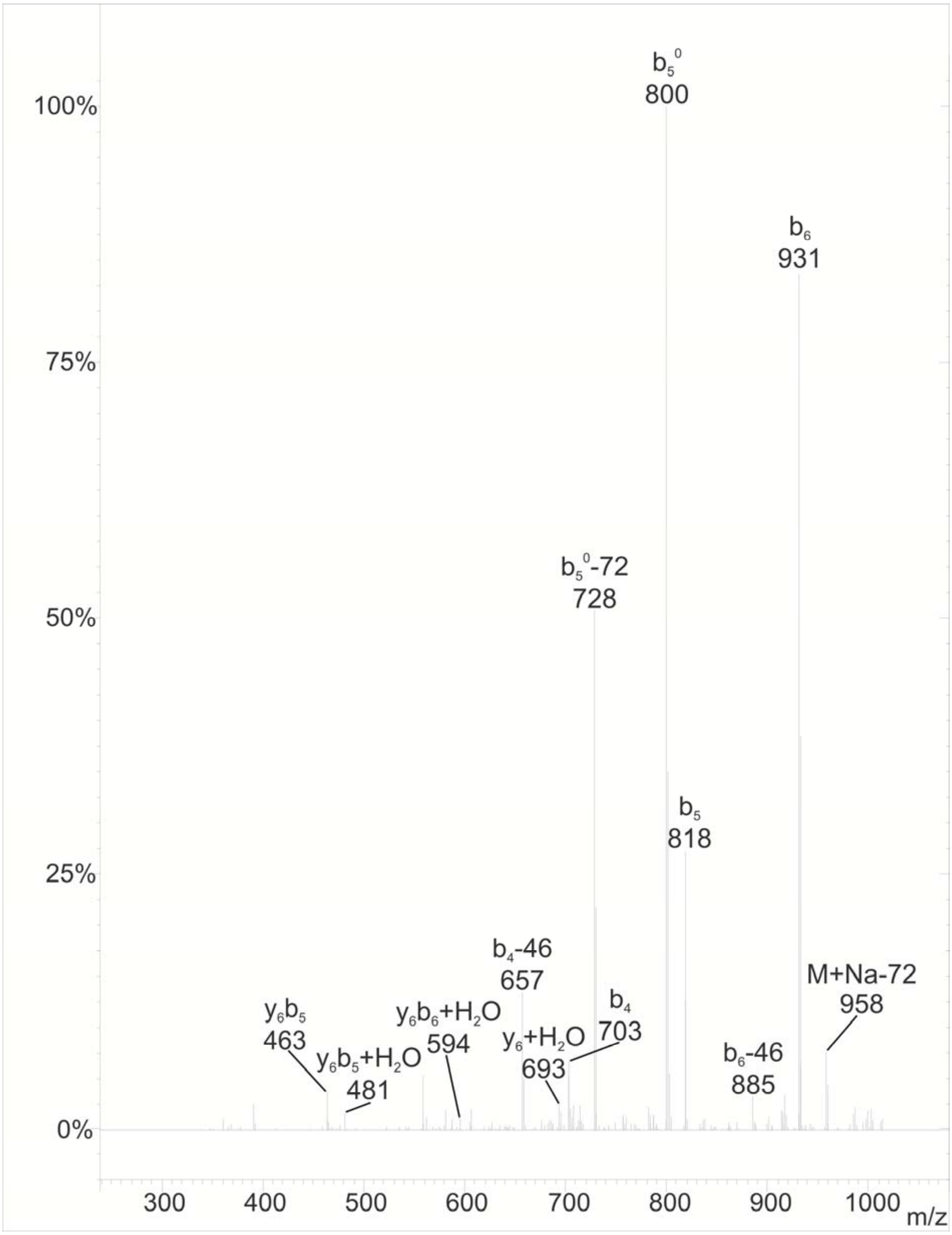


**Figure S12.** The MS[2] spectrum of **12** (C14-[Val7]) eluted at Rt = 82.66 min ($m/z$ = 1030).

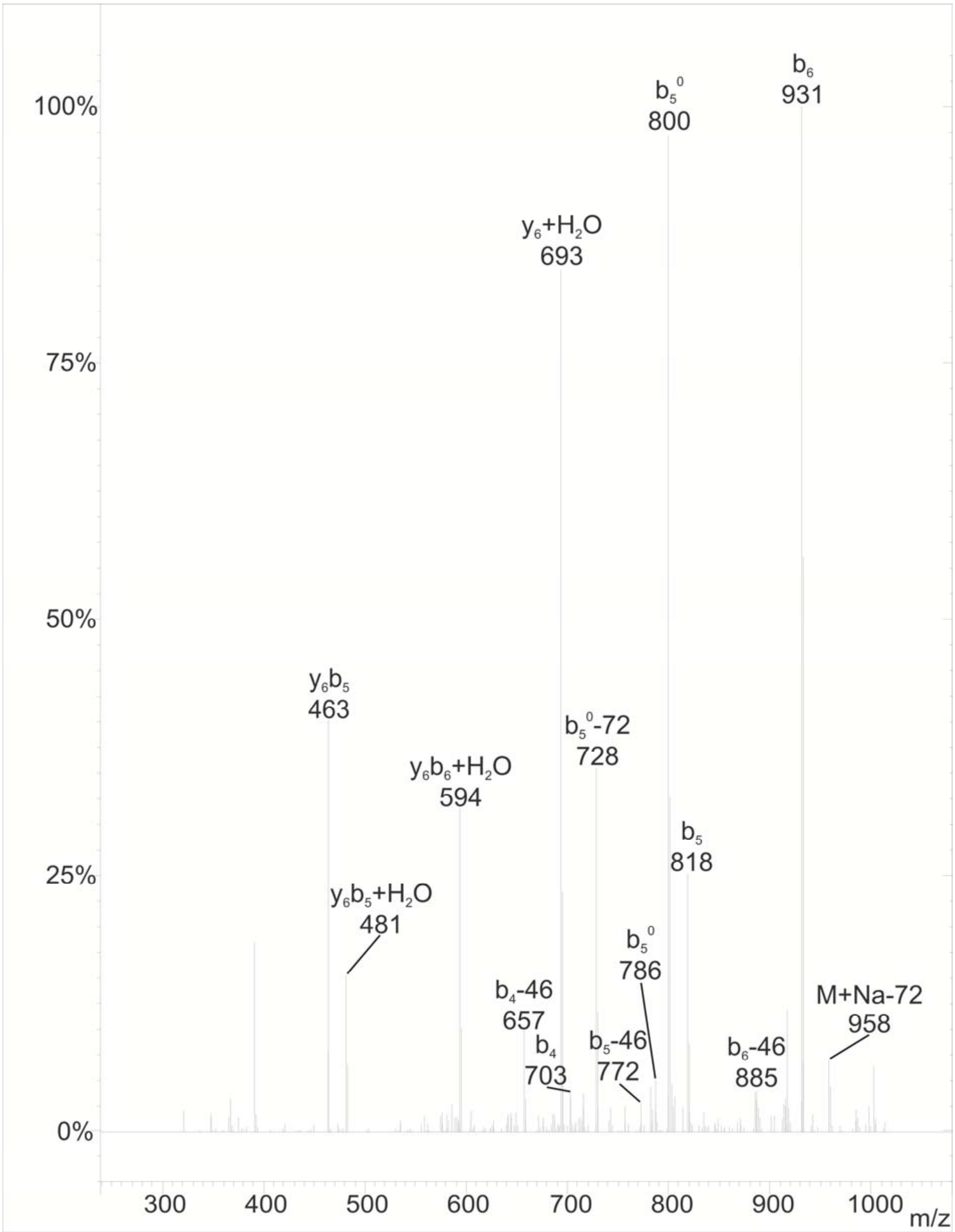


**Figure S13.** The MS[2] spectrum of **13** (C14-[Val7]) eluted at Rt = 83.66 min (*m*/*z* = 1030).

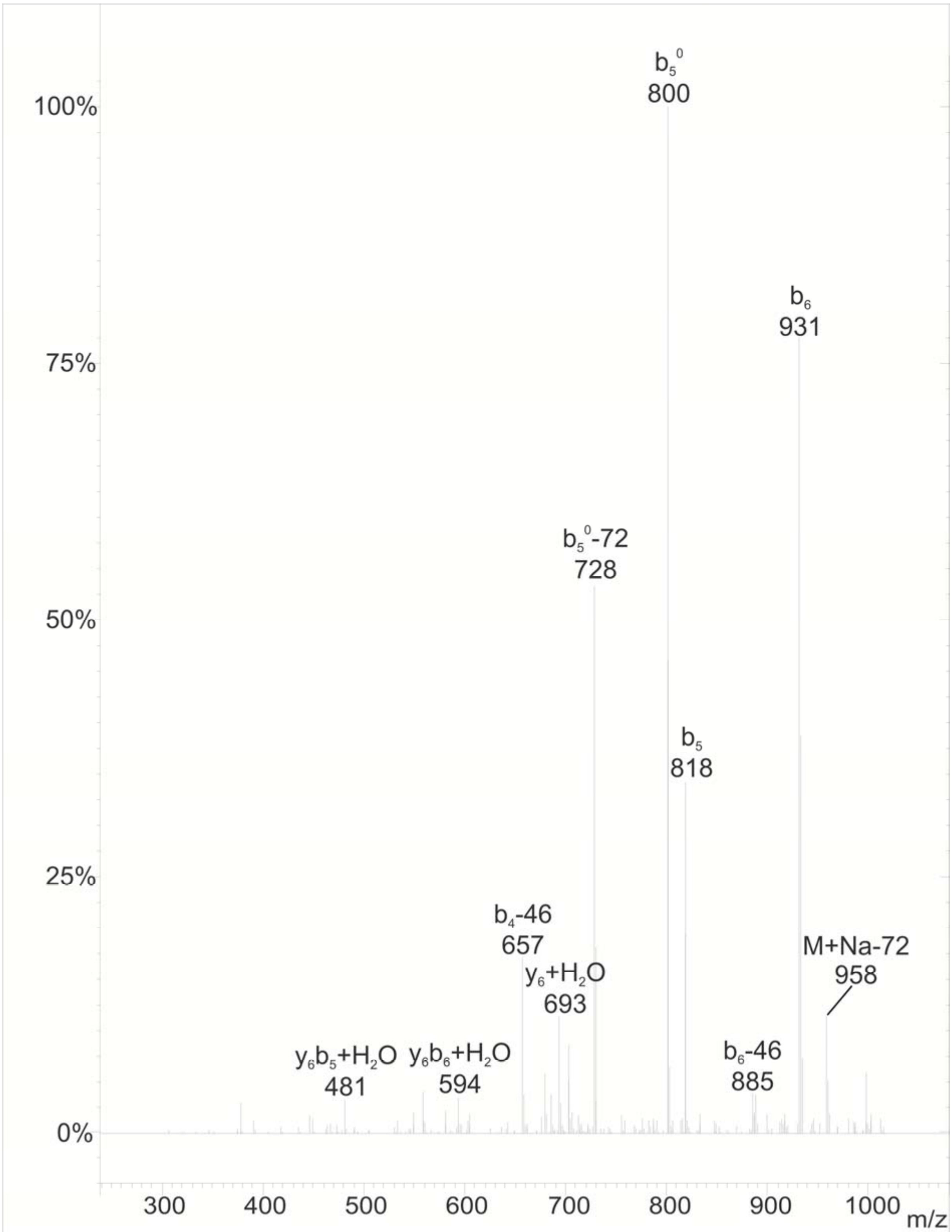


**Figure S14.** The MS$^2$ spectrum of **14** (C14-[Val7]) eluted at Rt = 84.57 min (*m*/*z* = 1030).

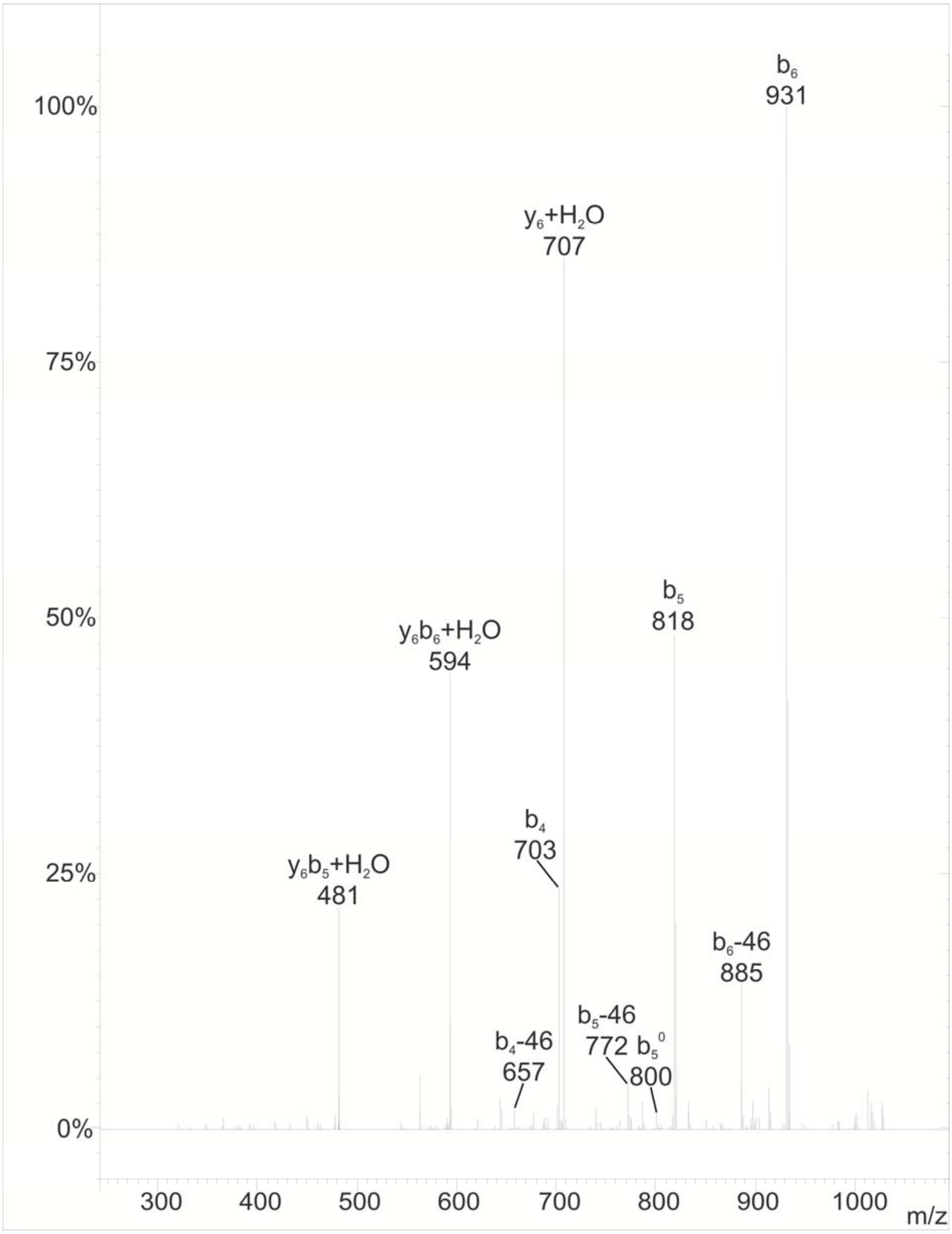


**Figure S15.** The MS[2] spectrum of **15** (C14-[Sur]) eluted at Rt = 81.55 min (*m/z* = 1044).

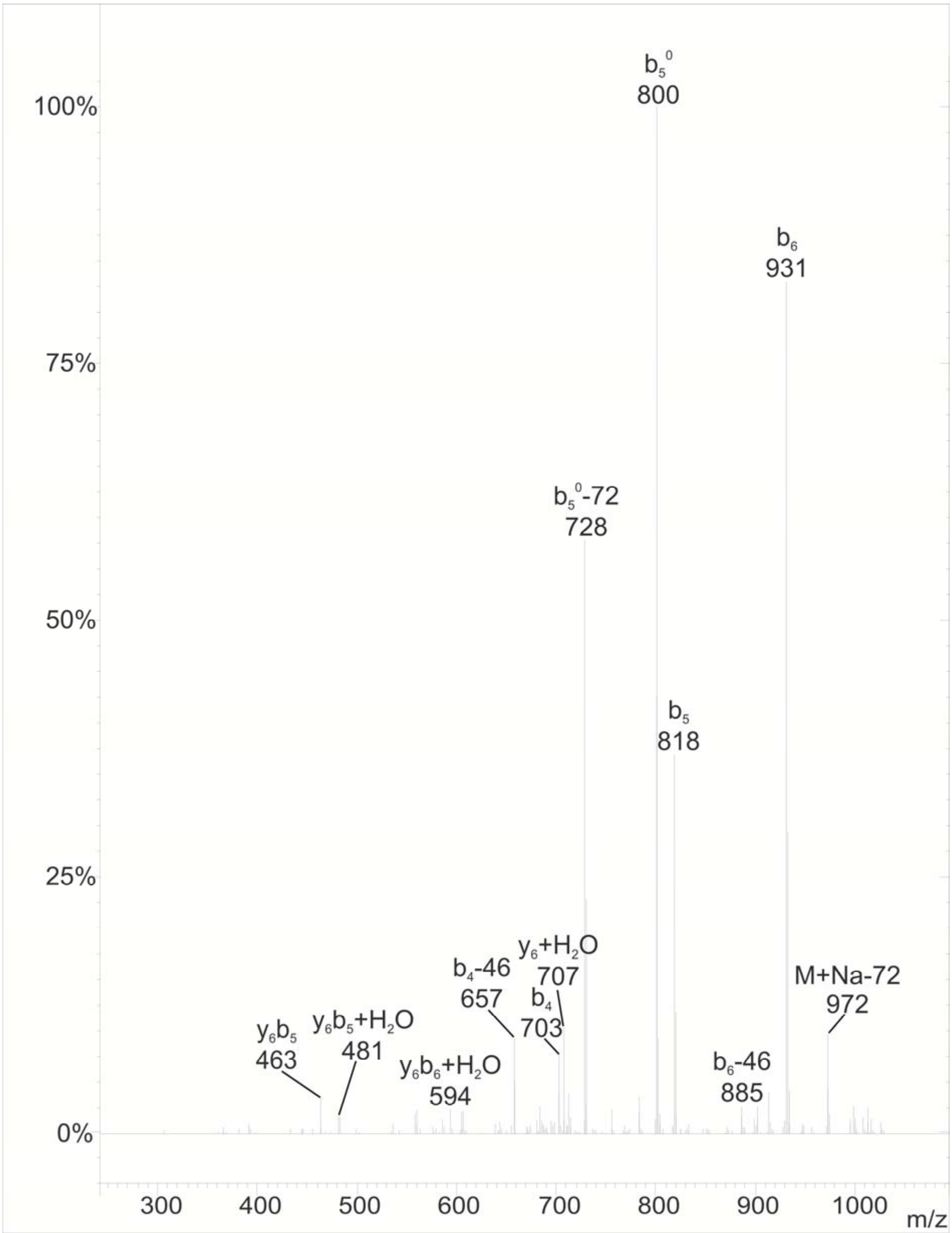


**Figure S16.** The MS$^2$ spectrum of **16** (C14-[Sur]) eluted at Rt = 82.13 min (*m/z* = 1044).

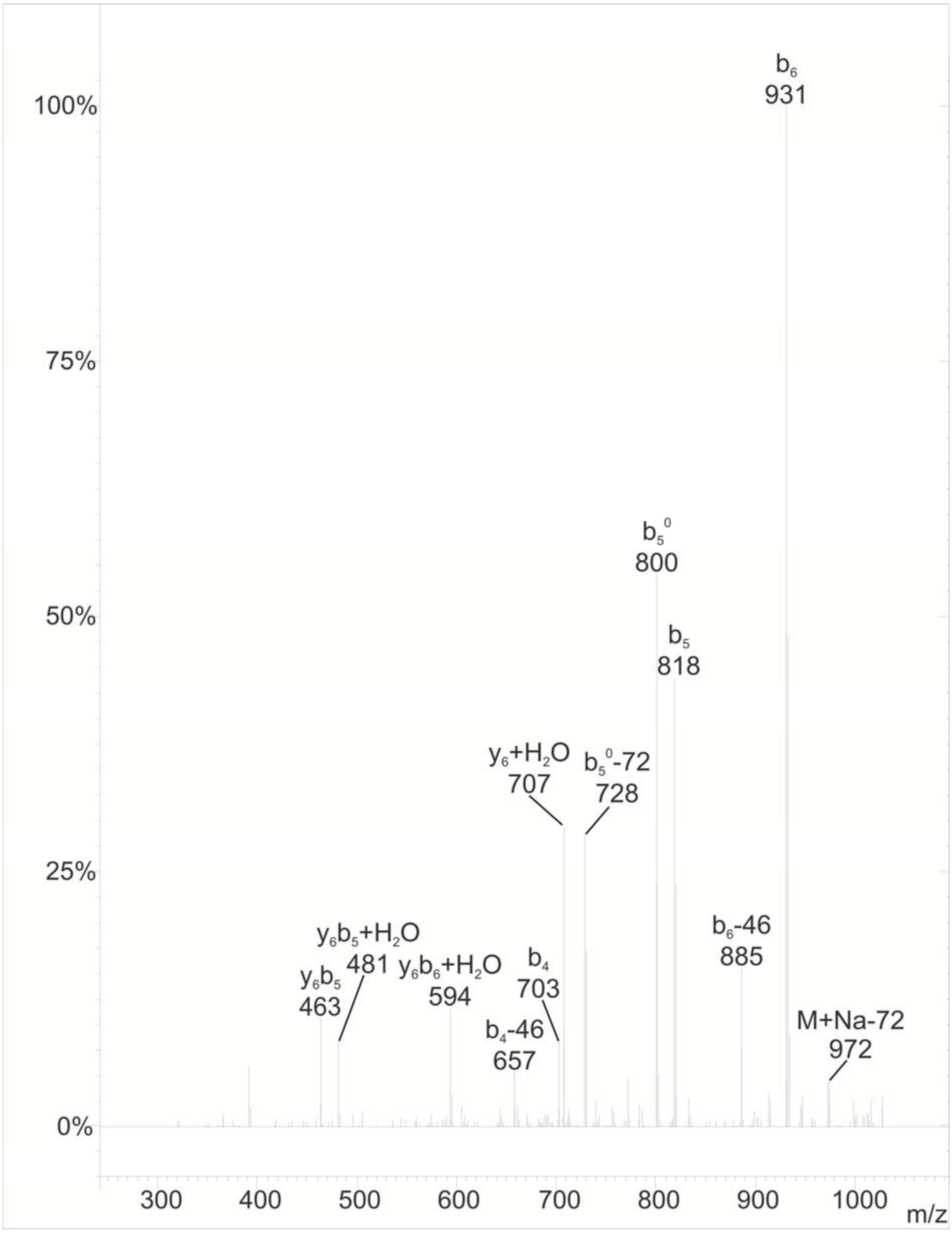


**Figure S17.** The MS[2] spectrum of **17** (C14-[Sur]) eluted at Rt = 83.14 min (*m/z* = 1044).

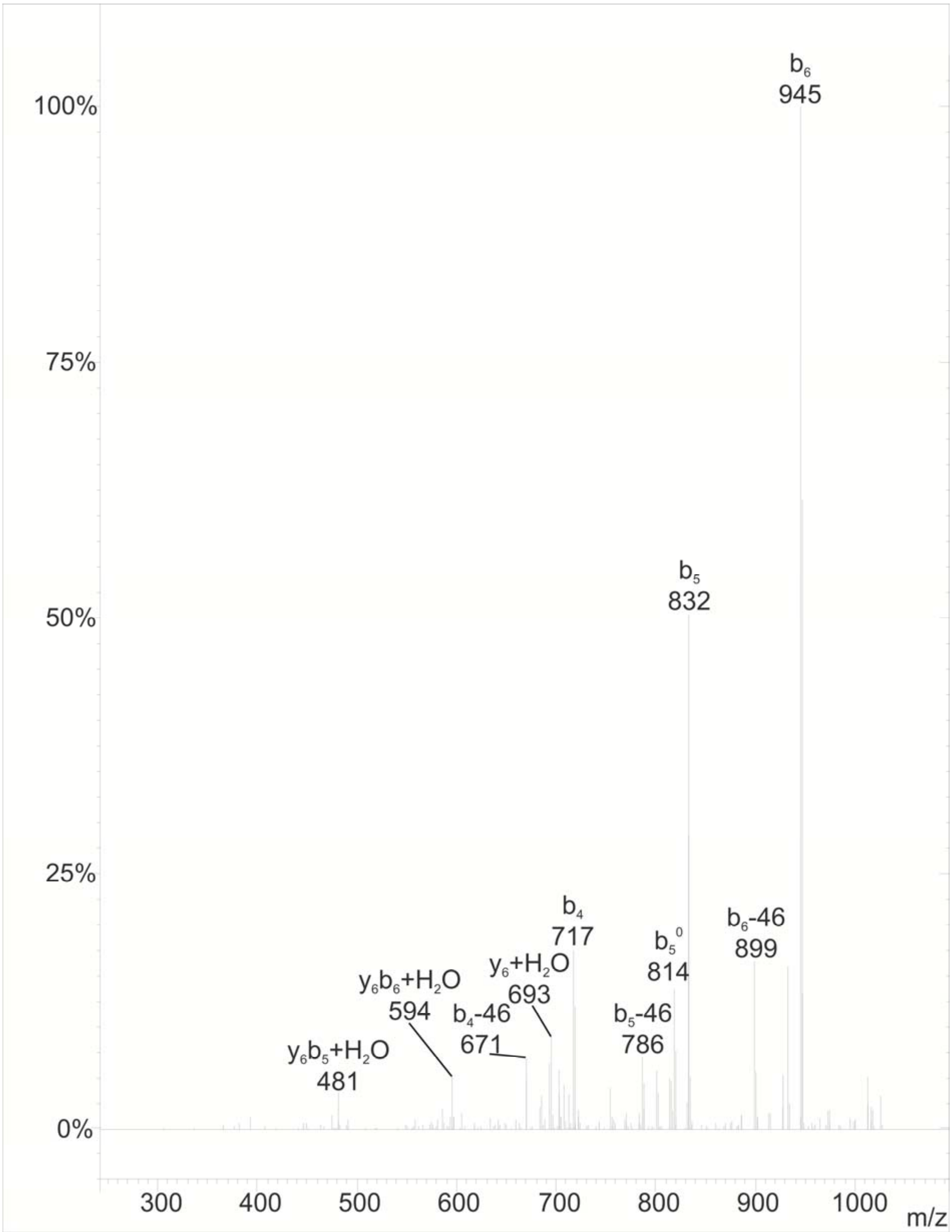


**Figure S18.** The MS$^2$ spectrum of **18** (C15-[Val7]) eluted at Rt = 84.08 min ($m/z$ = 1044).

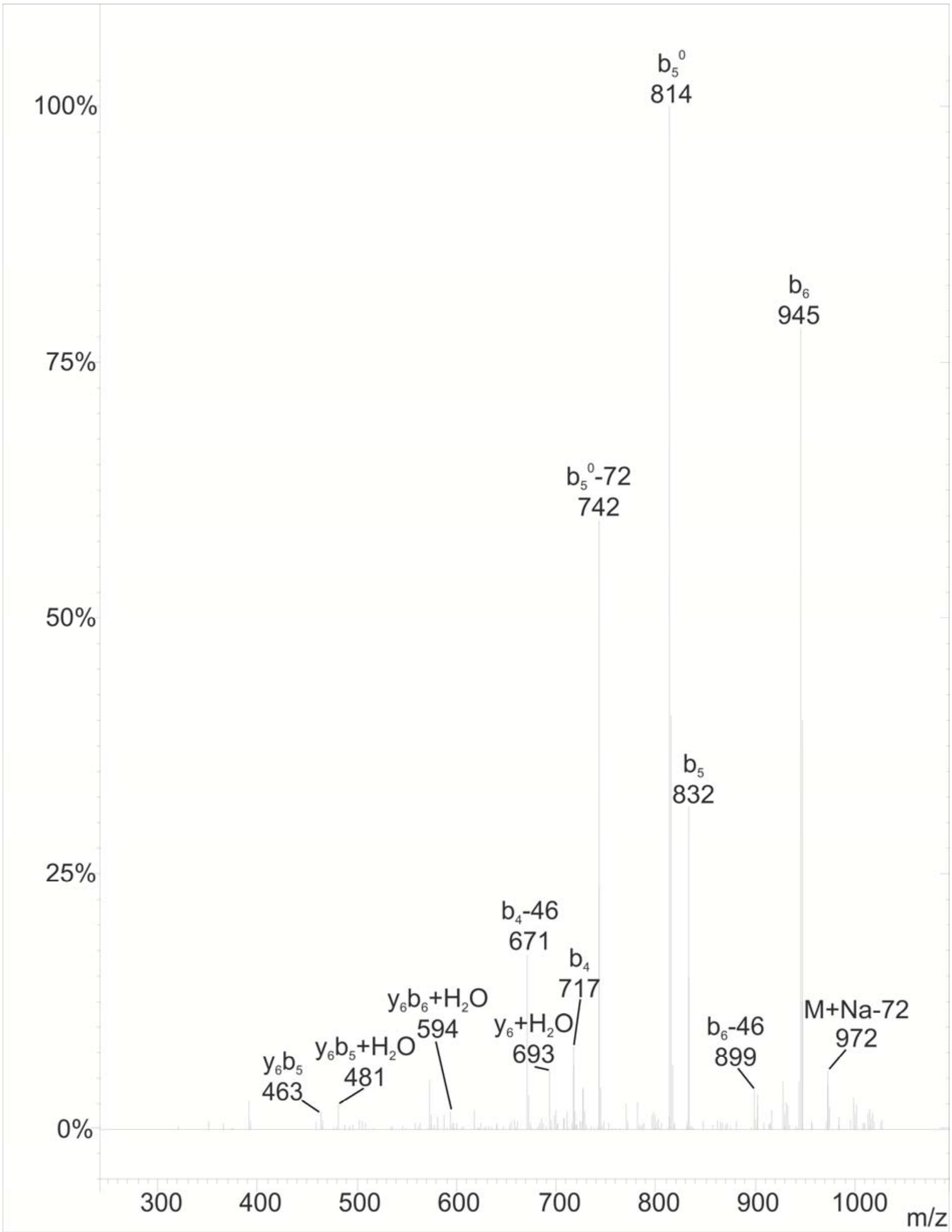


**Figure S19.** The MS[2] spectrum of **19** (C15-[Val7]) eluted at Rt = 85.44 min (*m*/*z* = 1044).

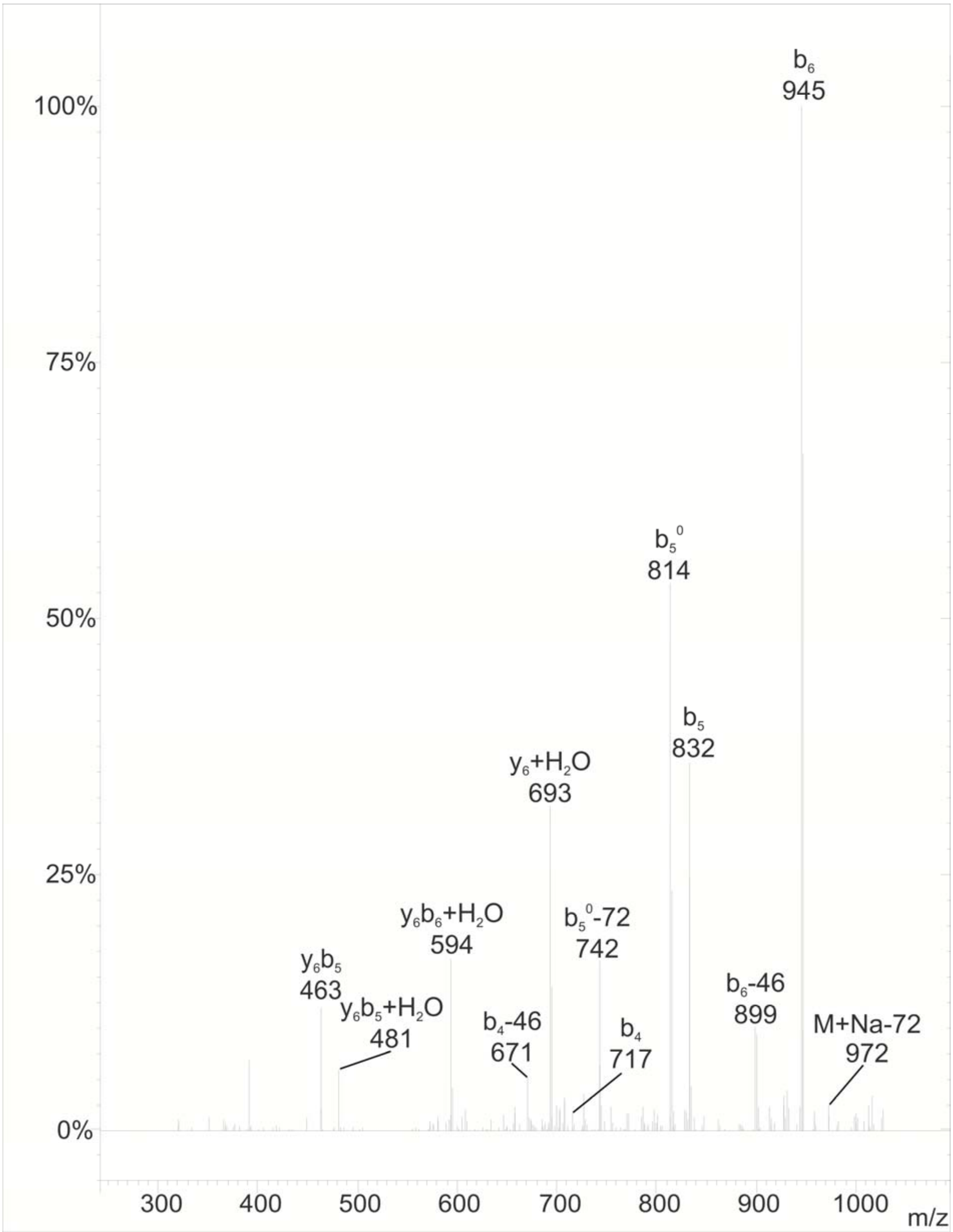


**Figure S20.** The MS[2] spectrum of **20** (C15-[Val7]) eluted at Rt = 86.32 min (*m*/*z* = 1044).

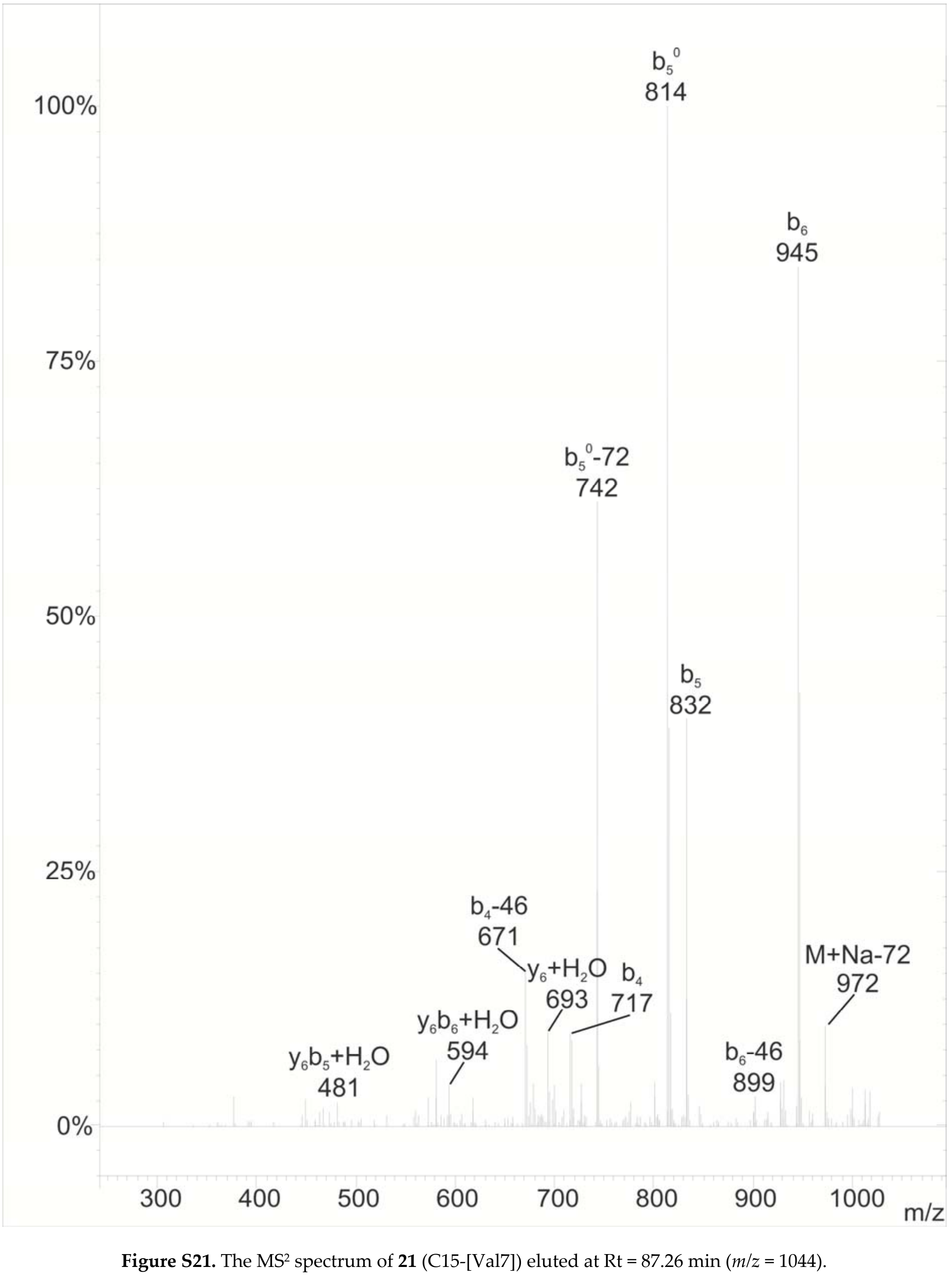


**Figure S21.** The MS[2] spectrum of **21** (C15-[Val7]) eluted at Rt = 87.26 min (*m*/*z* = 1044).

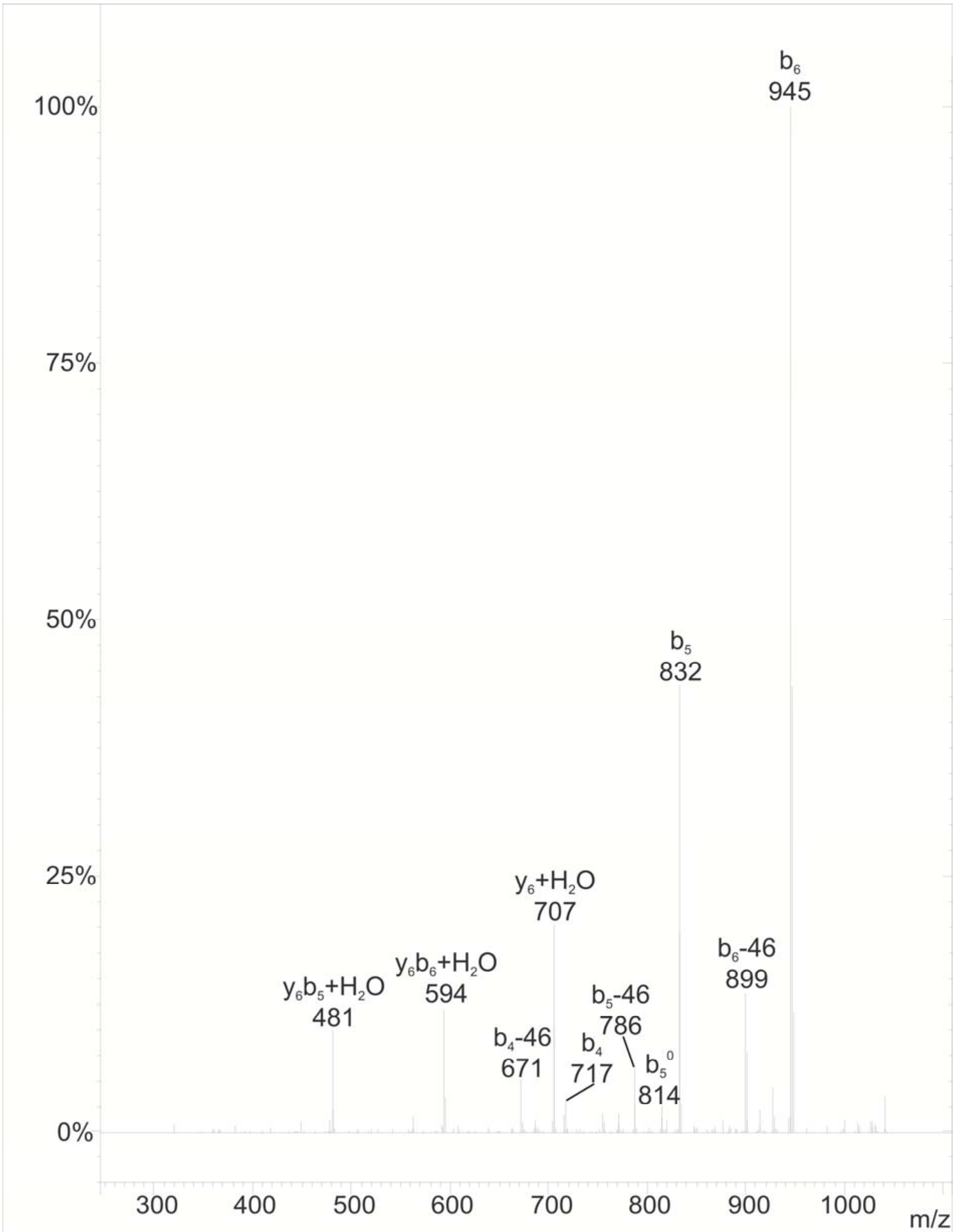


**Figure S22.** The MS[2] spectrum of **22** (C15-[Sur]) eluted at Rt = 84.18 min (*m/z* = 1058).

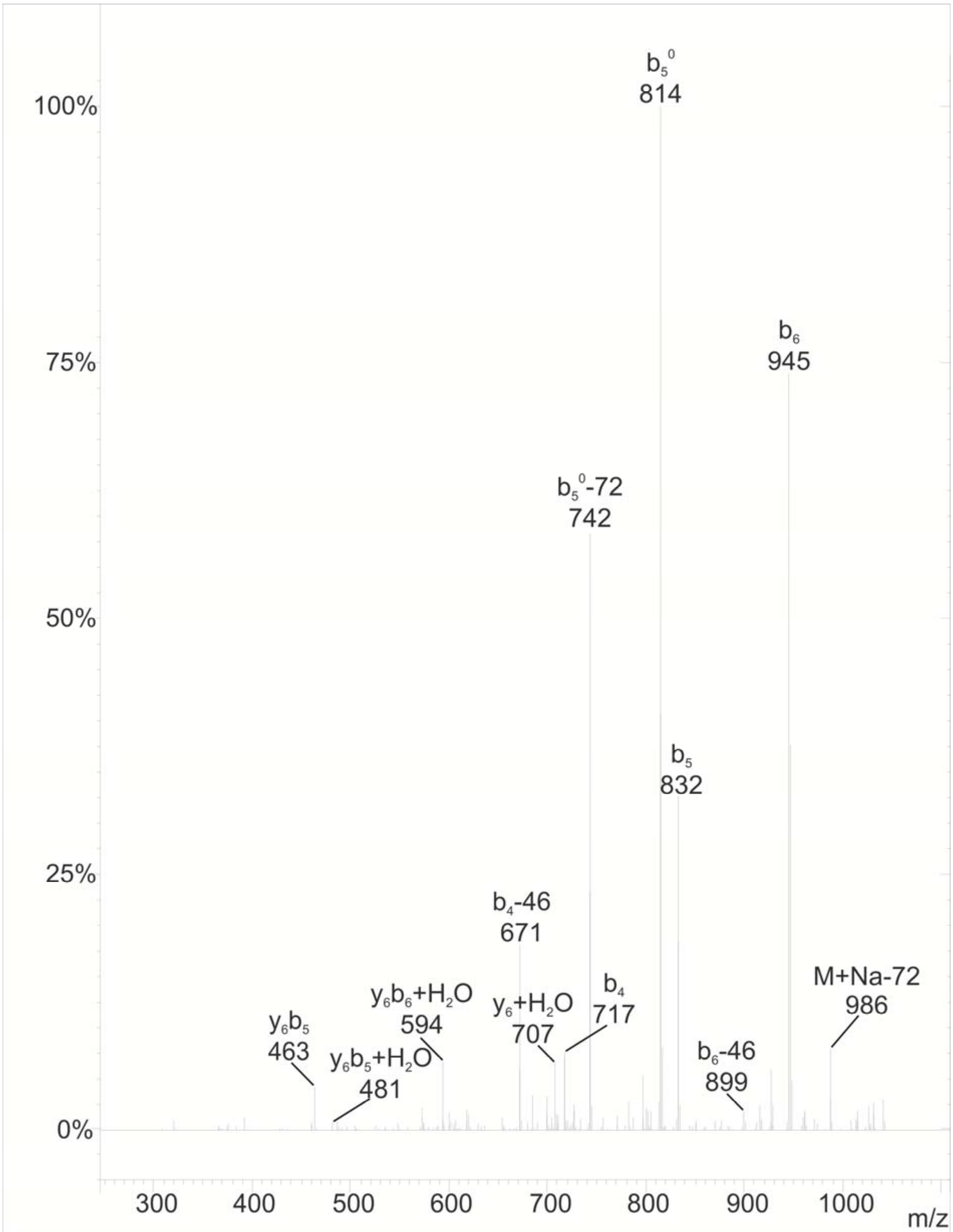


**Figure S23.** The MS² spectrum of **23** (C15-[Sur]) eluted at Rt = 84.70 min (*m/z* = 1058).

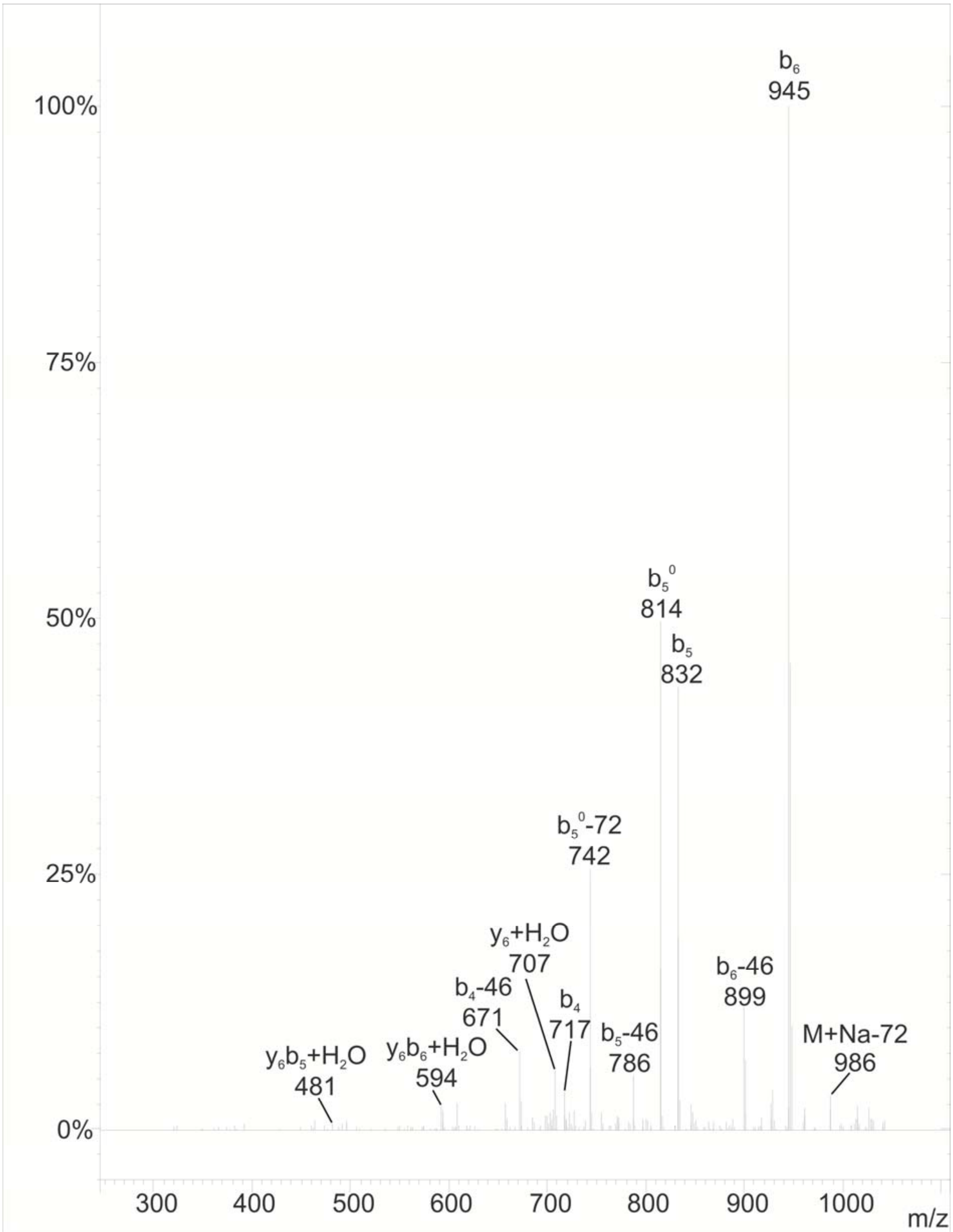


**Figure S24.** The MS[2] spectrum of **24** (C15-[Sur]) eluted at Rt = 86.01 min (*m/z* = 1058).

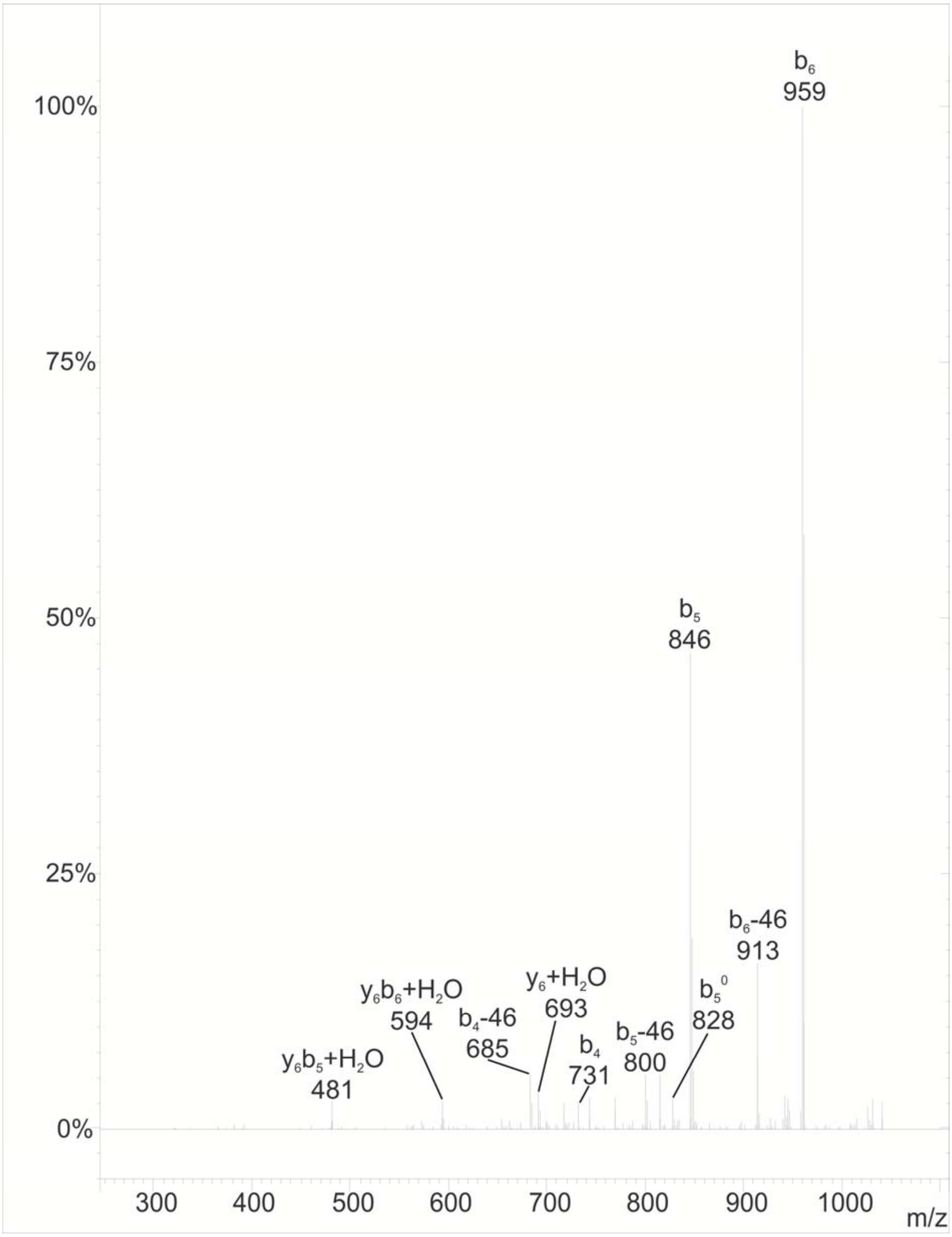


**Figure S25.** The MS[2] spectrum of **25** (C16-[Val7]) eluted at Rt = 87.02 min (*m*/*z* = 1058).

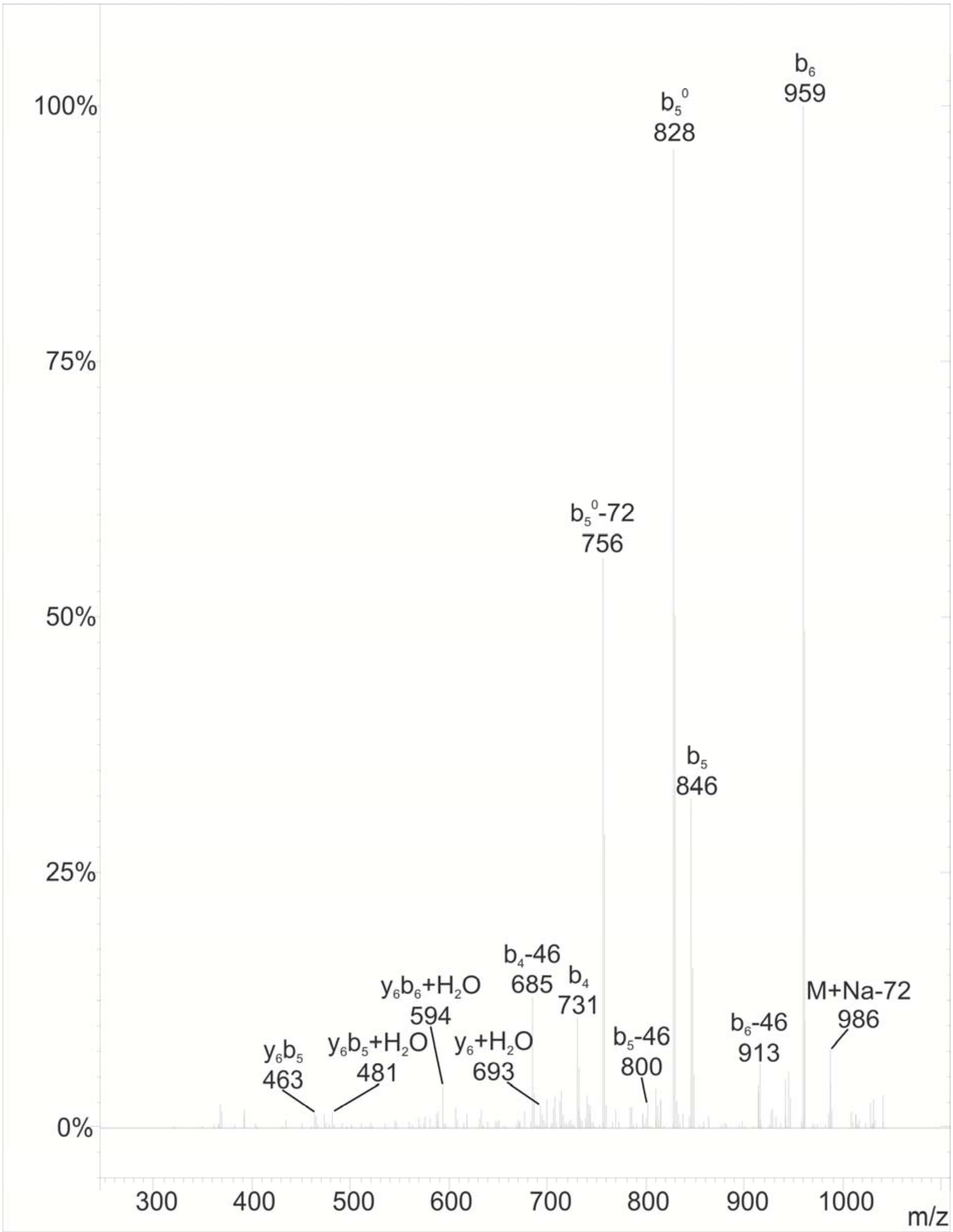


**Figure S26.** The MS[2] spectrum of **26** (C16-[Val7]) eluted at Rt = 88.18 min ($m/z$ = 1058).

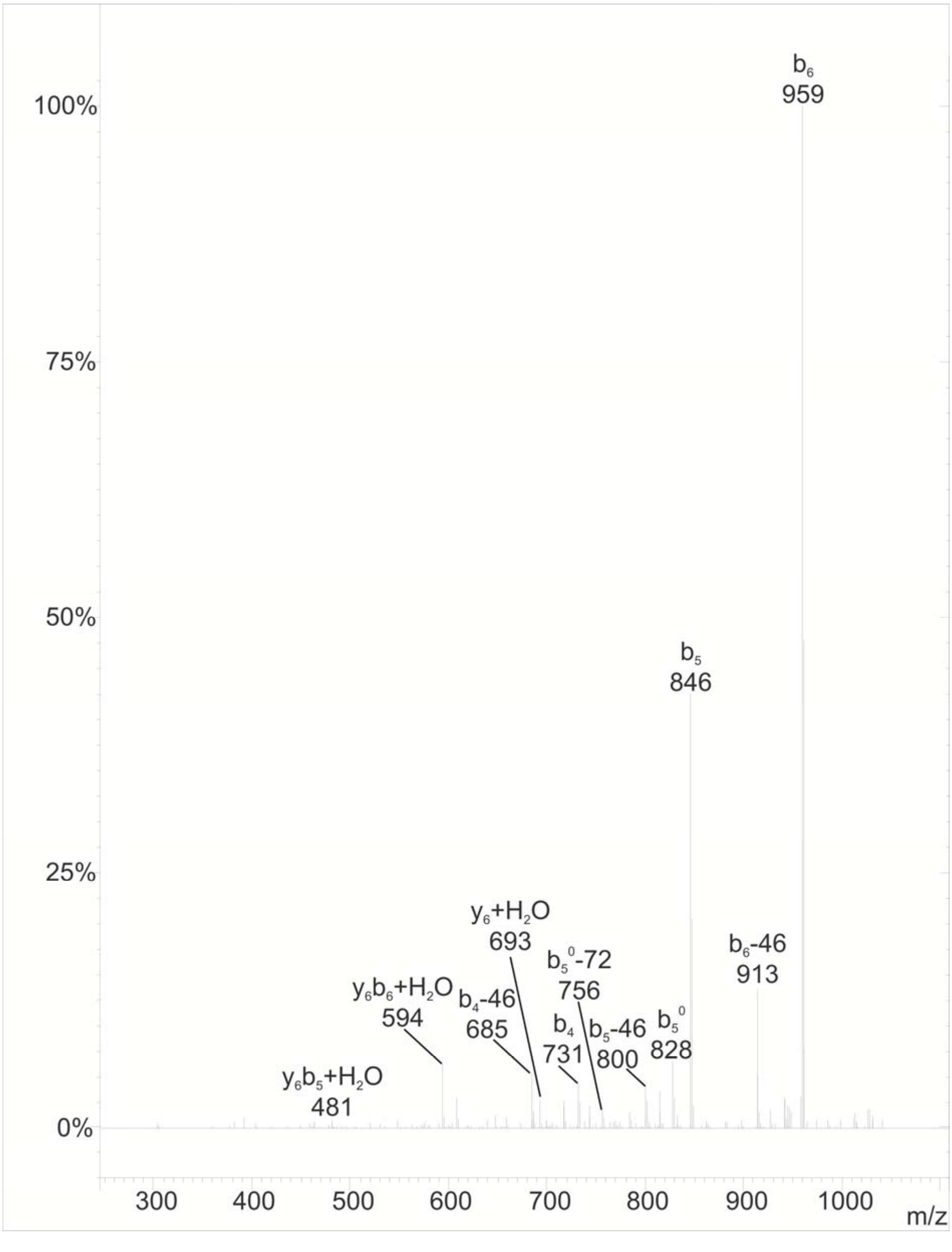


**Figure S27.** The MS[2] spectrum of **27** (C16-[Val7]) eluted at Rt = 89.15 min (*m*/*z* = 1058).

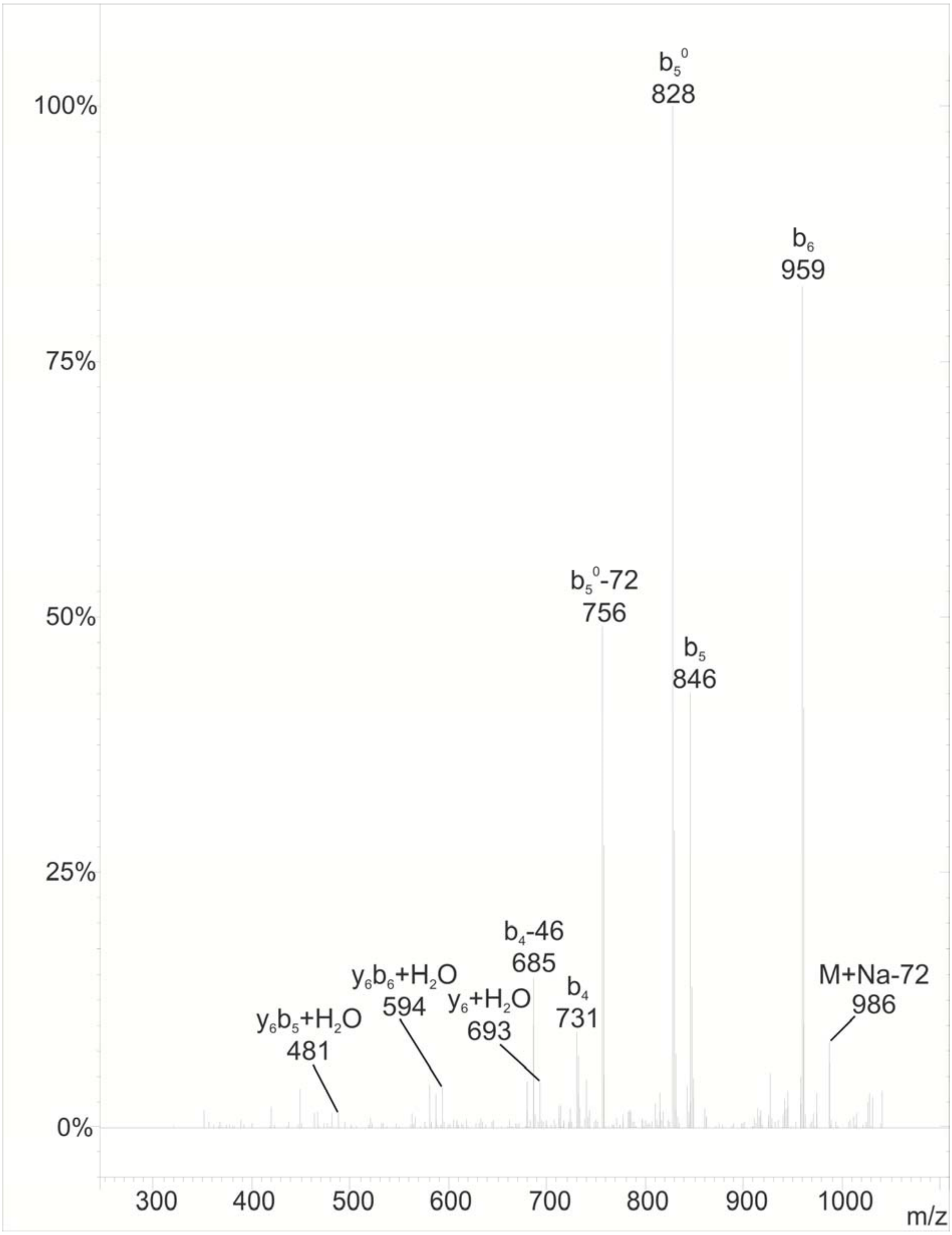


**Figure S28.** The $MS^2$ spectrum of **28** (C16-[Val7]) eluted at Rt = 90.68 min ($m/z$ = 1058).

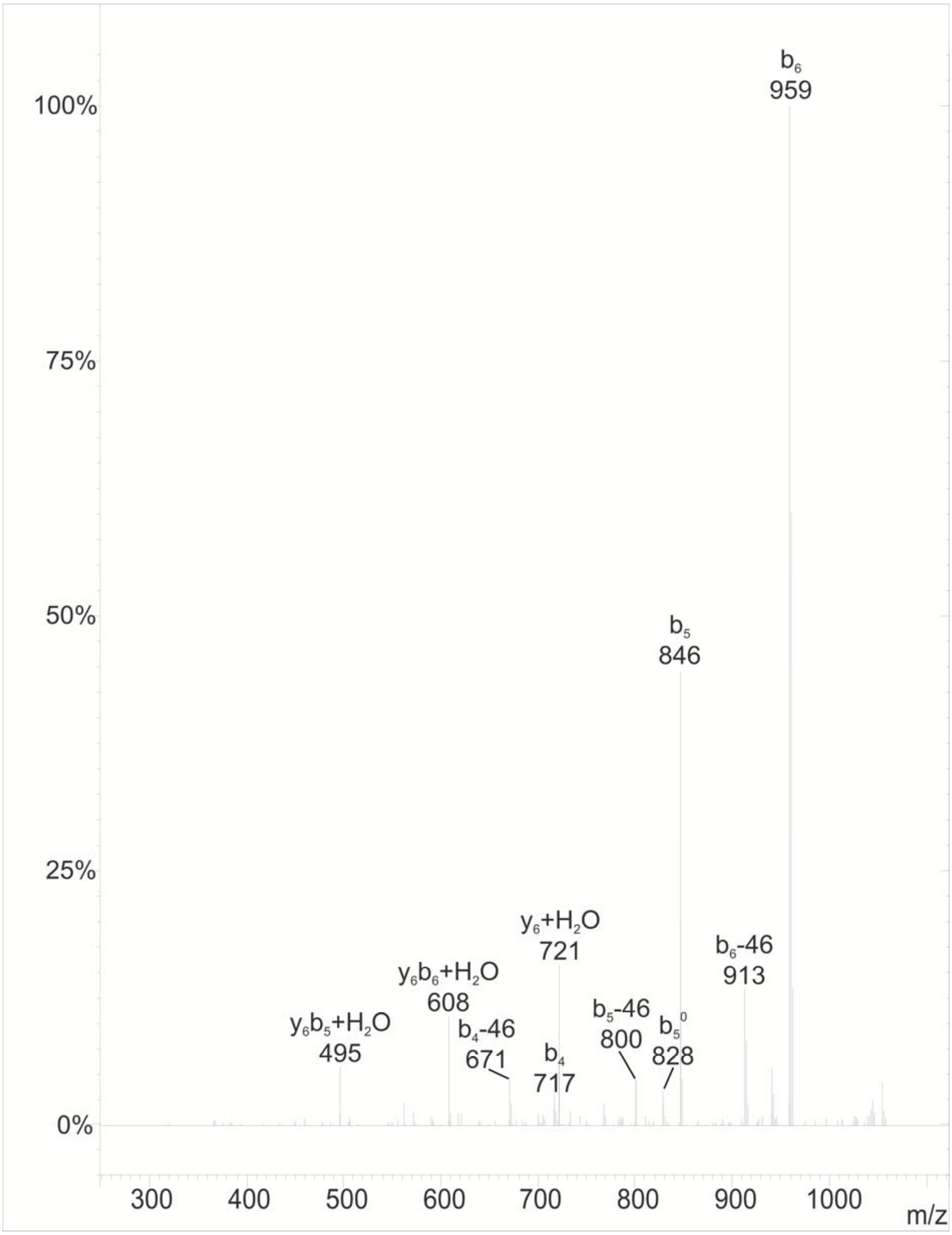


**Figure S29.** The MS[2] spectrum of **29** (C15-[AME5]) eluted at Rt = 86.80 min (*m*/*z* = 1072).

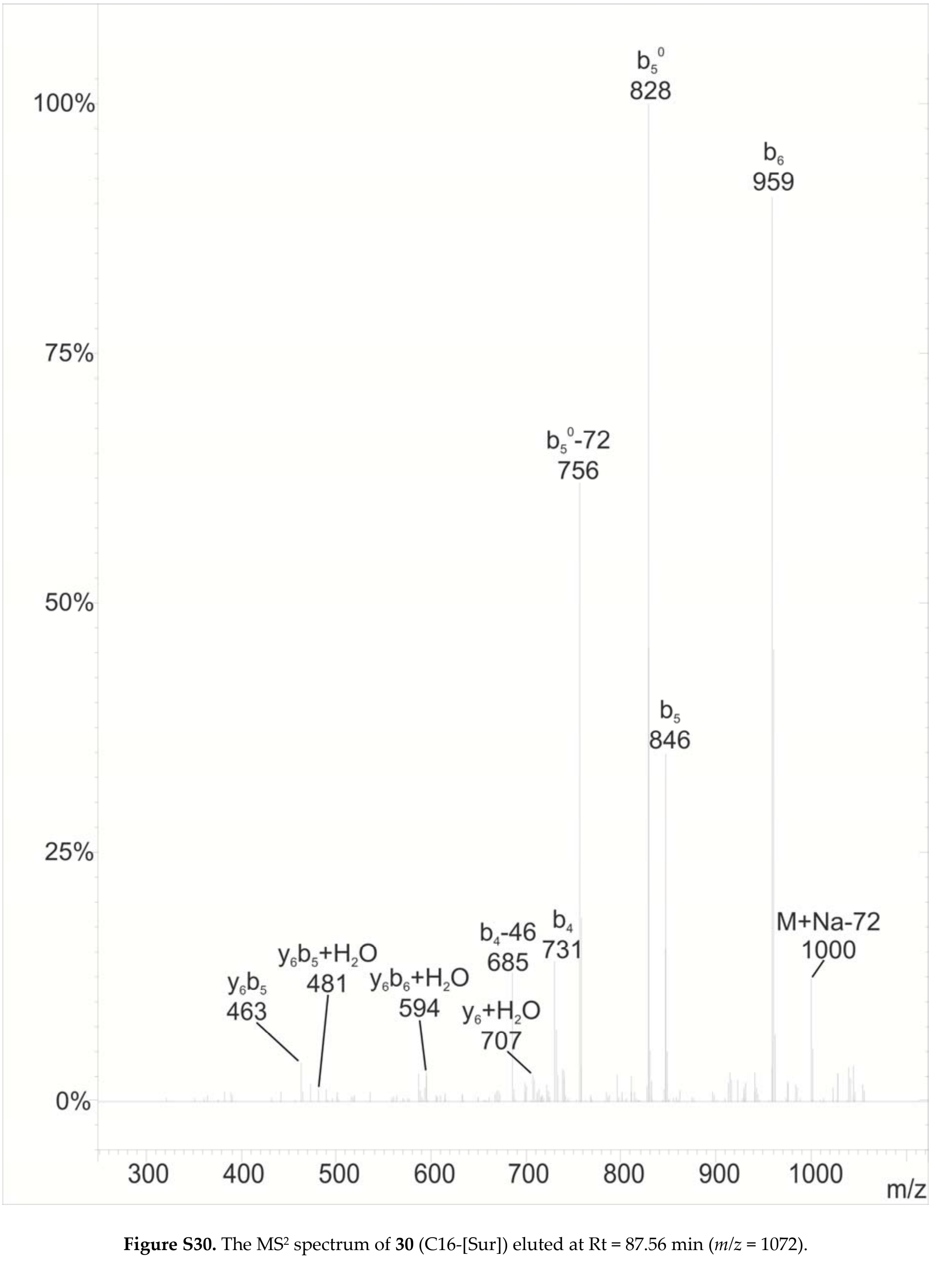


**Figure S30.** The MS[2] spectrum of **30** (C16-[Sur]) eluted at Rt = 87.56 min (*m/z* = 1072).

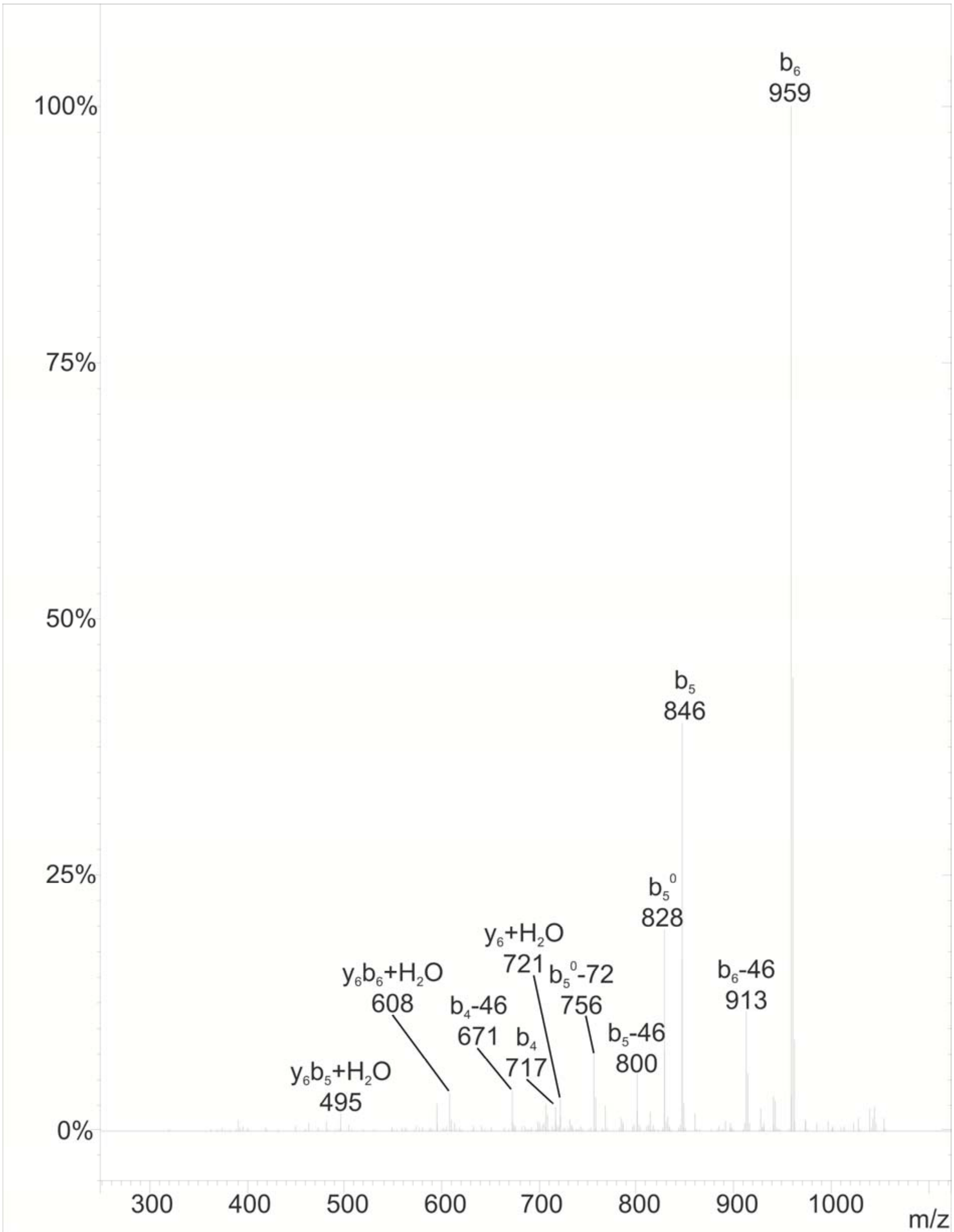


**Figure S31.** The MS[2] spectrum of **31** (C15-[AME5]) eluted at Rt = 88.77 min (*m*/*z* = 1072).

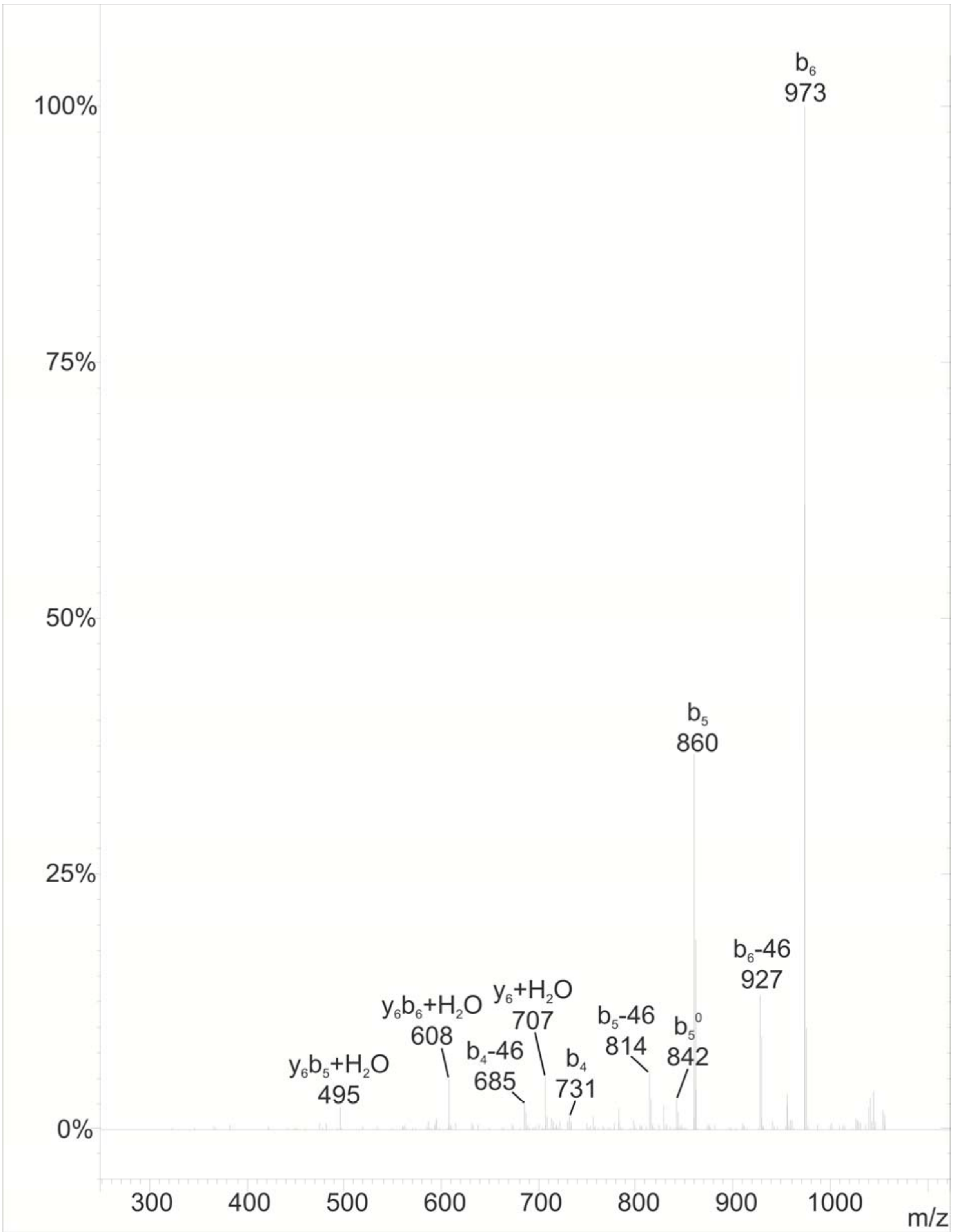


**Figure S32.** The MS² spectrum of **32** (C16-[AME5,Val7]) eluted at Rt = 89.71 min (*m*/*z* = 1072).

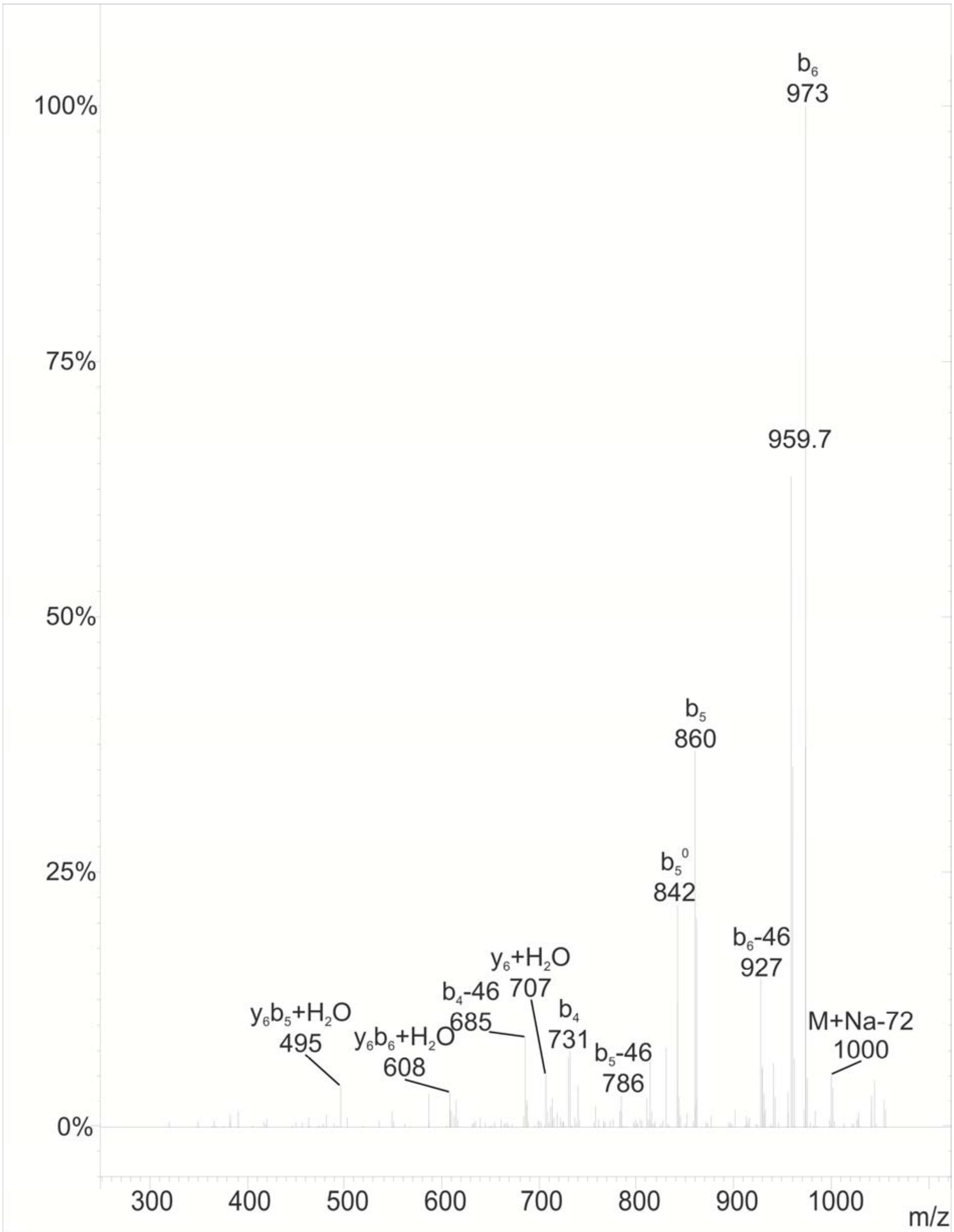


**Figure S33.** The MS$^2$ spectrum of **33** (C16-[AME5,Val7]) eluted at Rt = 90.13 min (*m*/*z* = 1072).

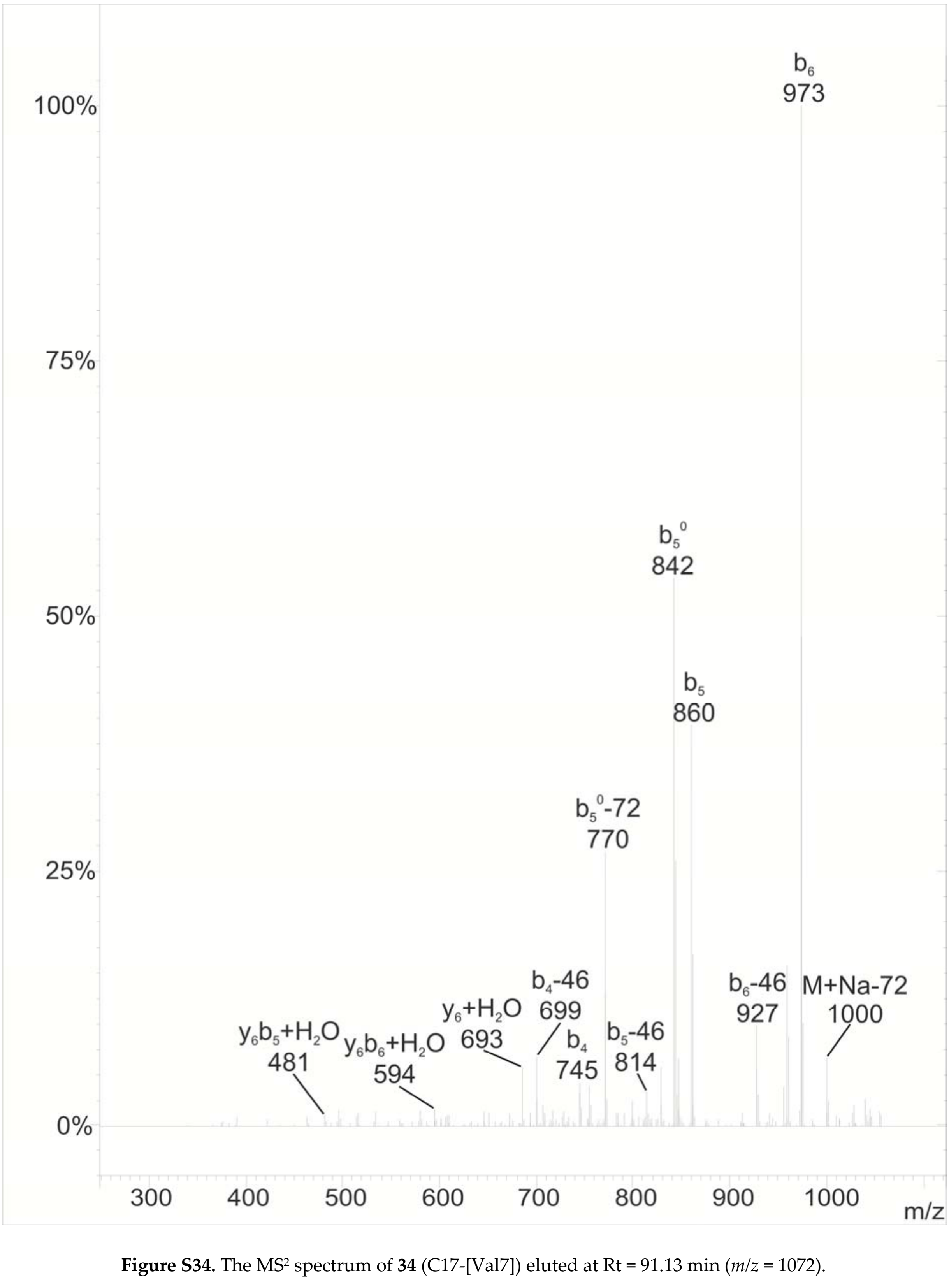


**Figure S34.** The $MS^2$ spectrum of **34** (C17-[Val7]) eluted at Rt = 91.13 min (*m*/*z* = 1072).

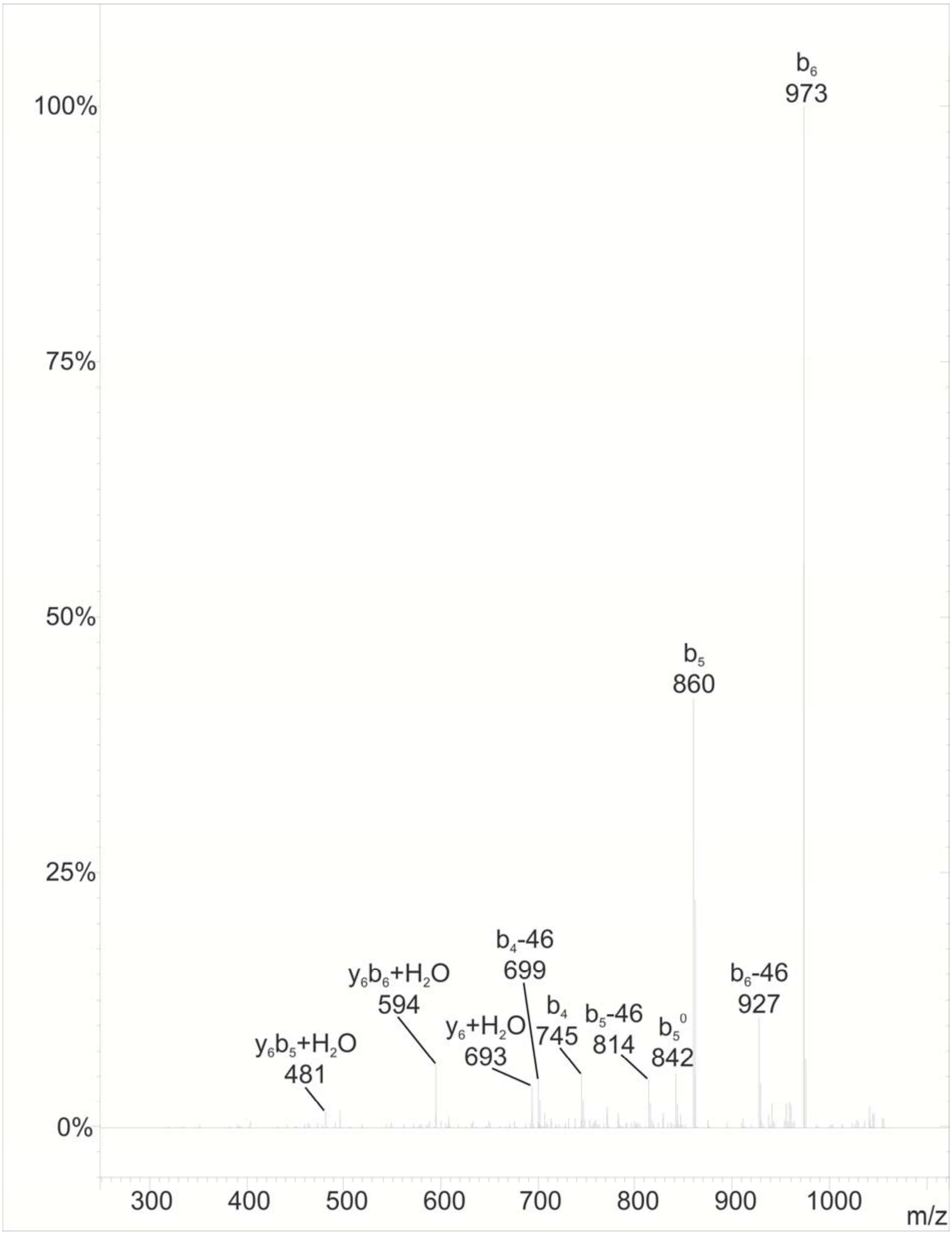


**Figure S35.** The MS[2] spectrum of **35** (C17-[Val7]) eluted at Rt = 92.02 min ($m/z$ = 1072).

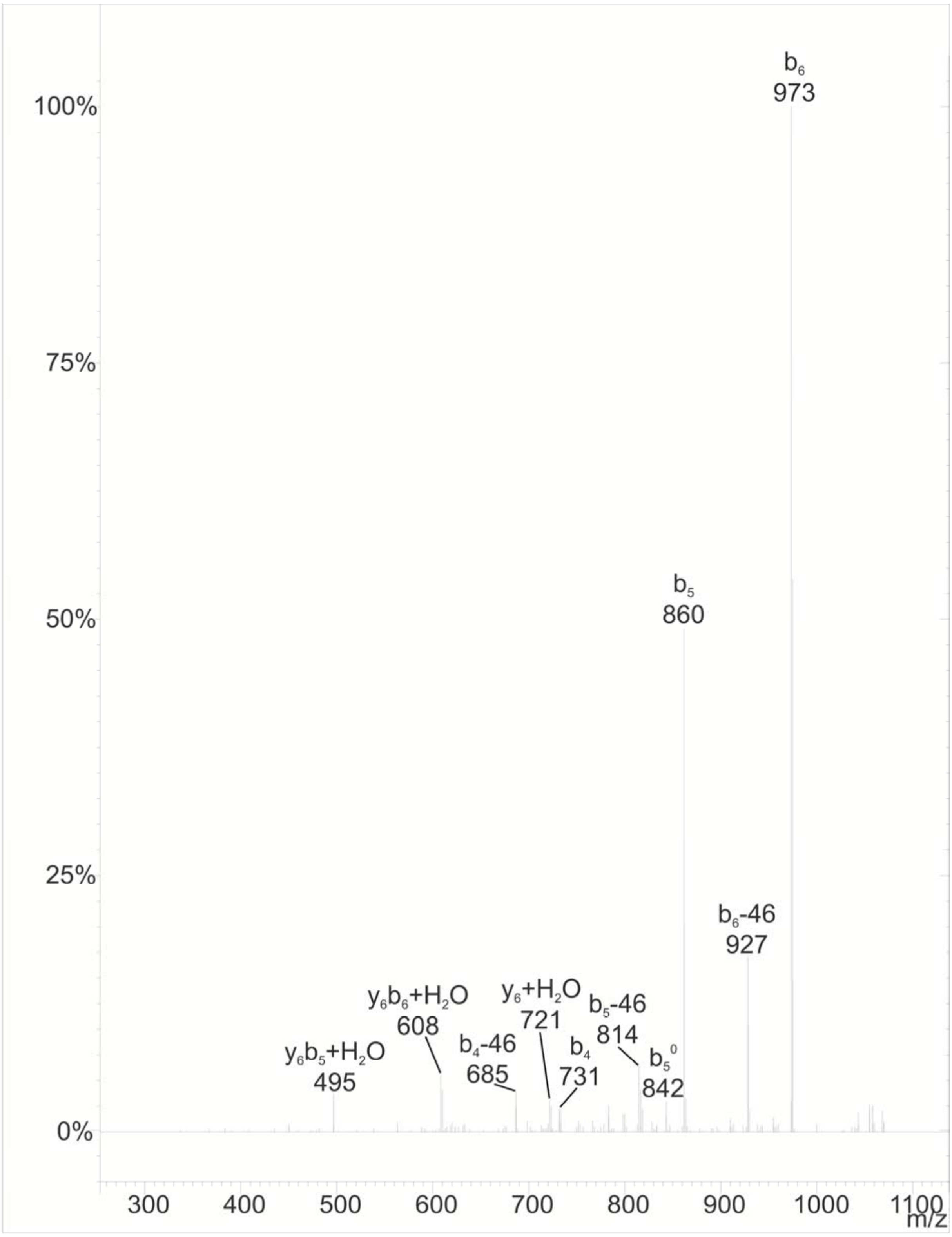


**Figure S36.** The MS² spectrum of **36** (C16-[AME5]) eluted at Rt = 89.41 min (*m*/*z* = 1086).

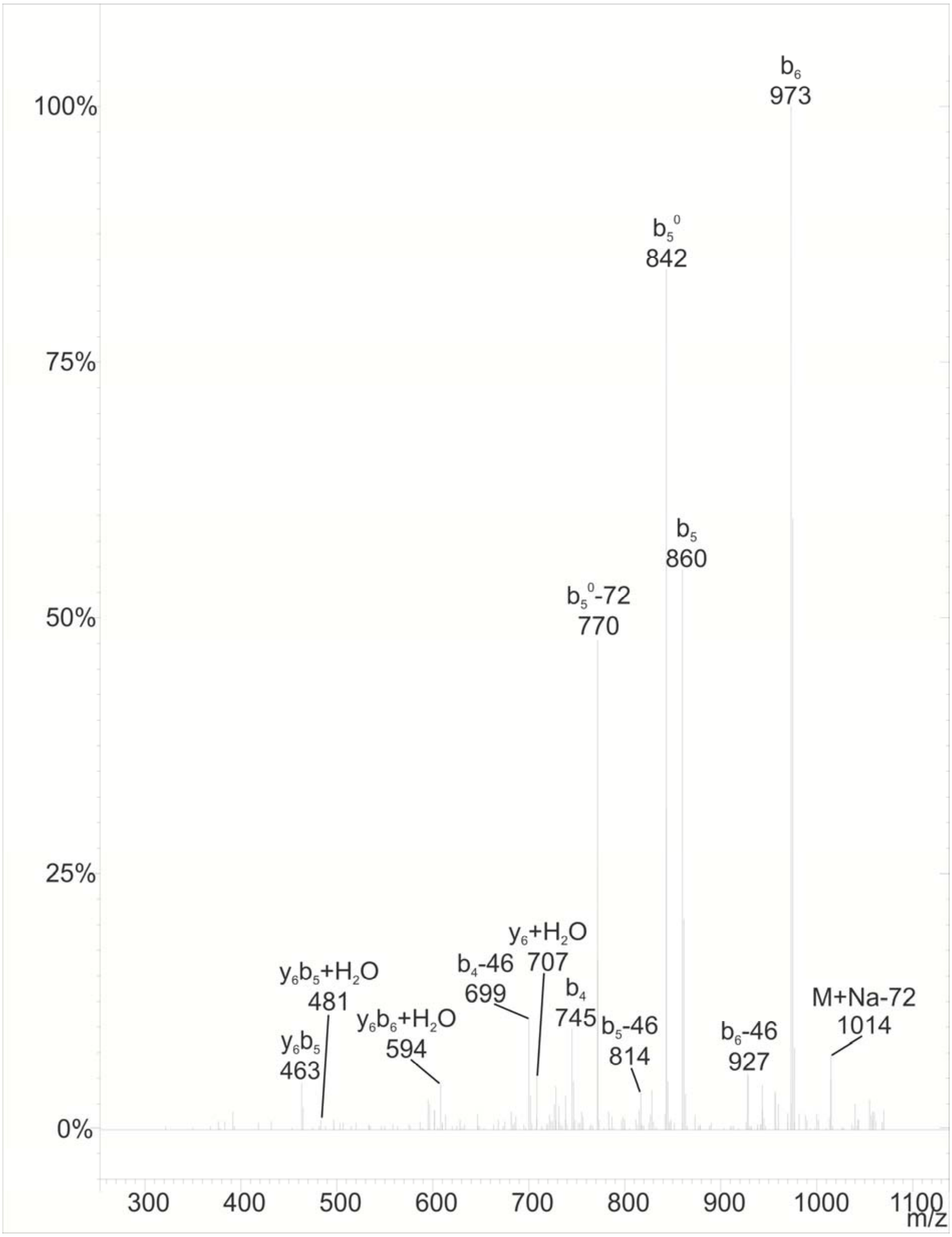


**Figure S37.** The MS[2] spectrum of **37** (C17-[Sur]) eluted at Rt = 90.50 min (*m*/*z* = 1086).

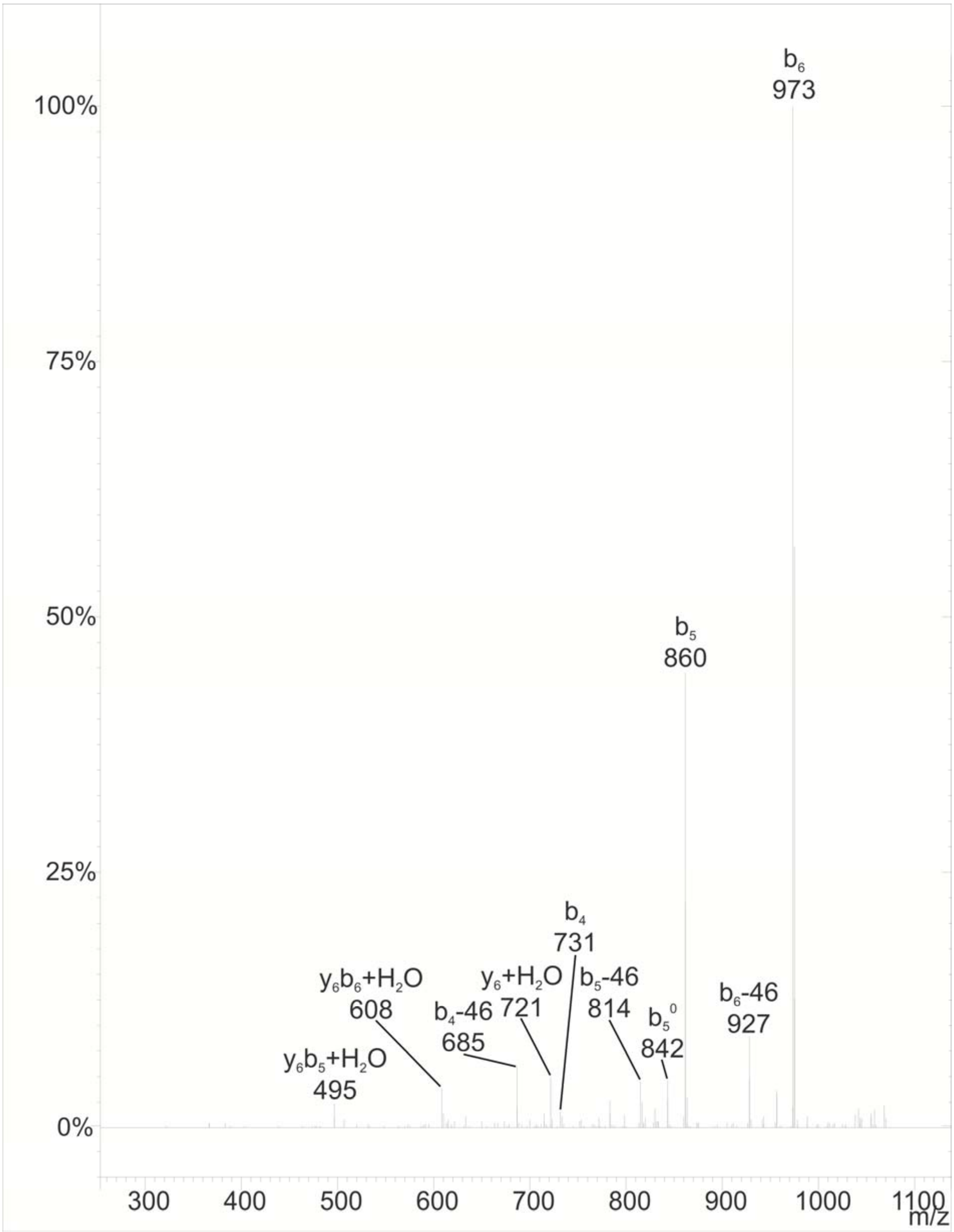


**Figure S38.** The MS[2] spectrum of **38** (C16-[AME5]) eluted at Rt = 91.61 min (*m*/*z* = 1086).

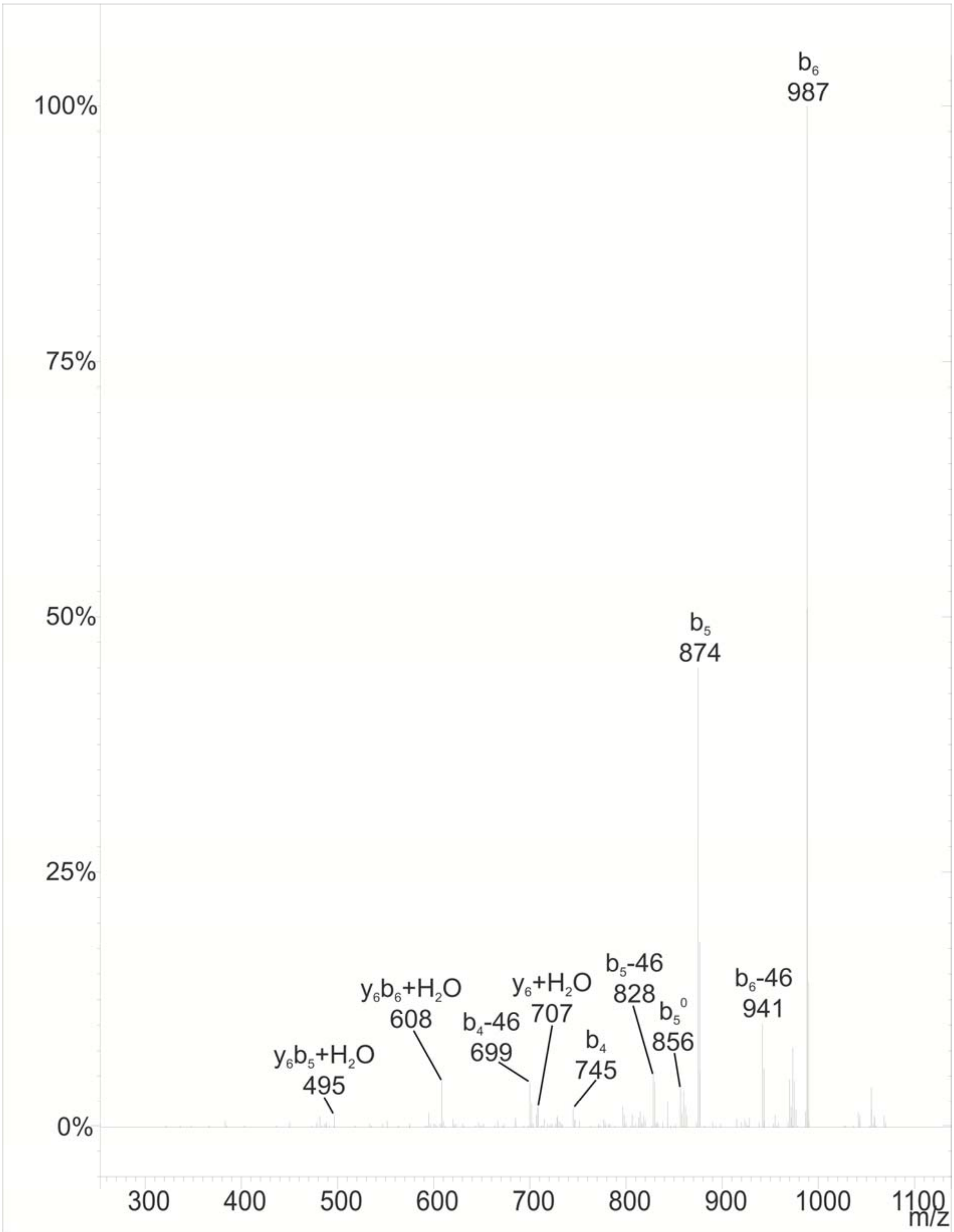


**Figure S39.** The $MS^2$ spectrum of **39** (C17-[AME5,Val7]) eluted at Rt = 92.61 min (*m*/*z* = 1086).

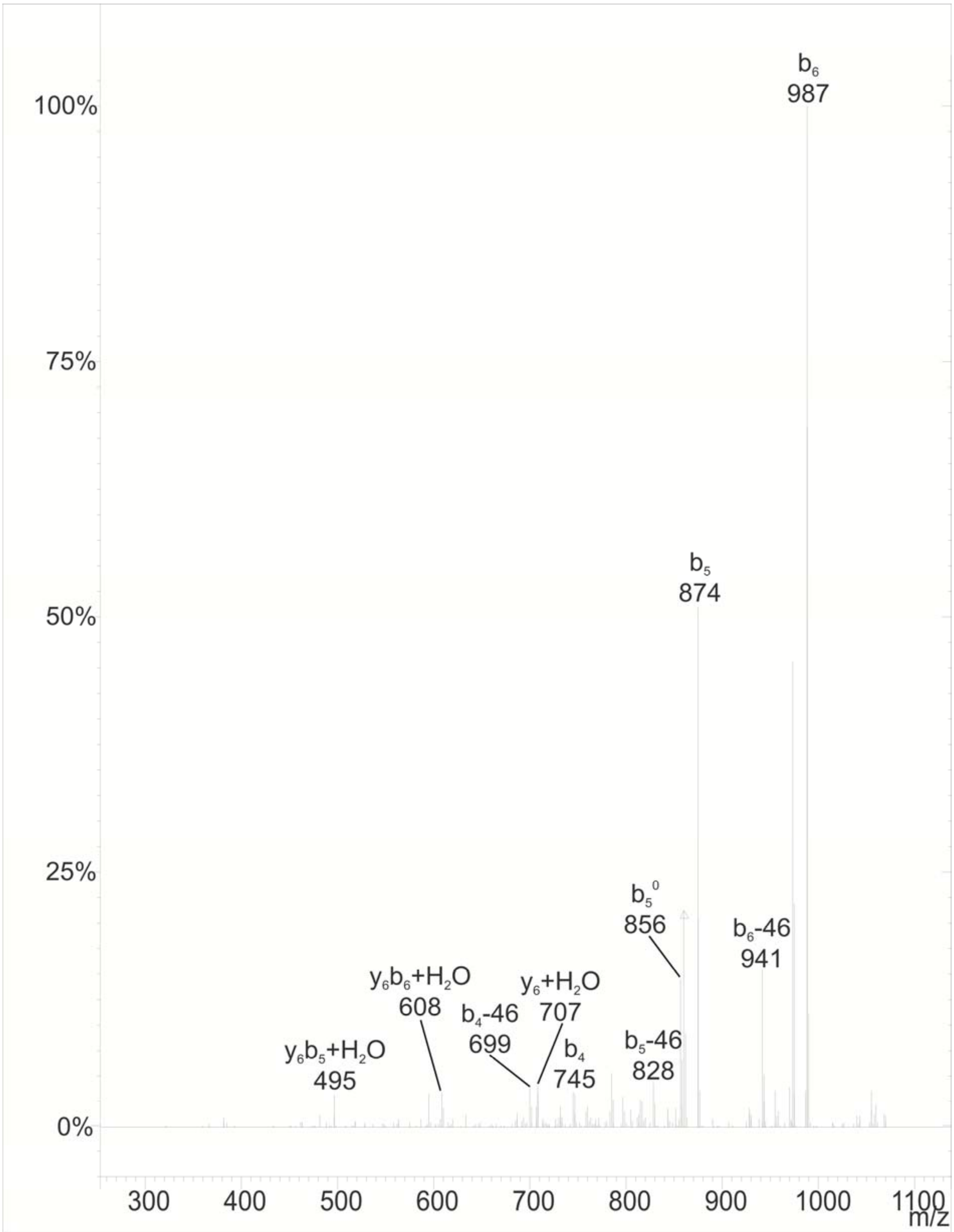


**Figure S40.** The MS[2] spectrum of **40** (C17-[AME5,Val7]) eluted at Rt = 93.69 min (*m*/*z* = 1086).

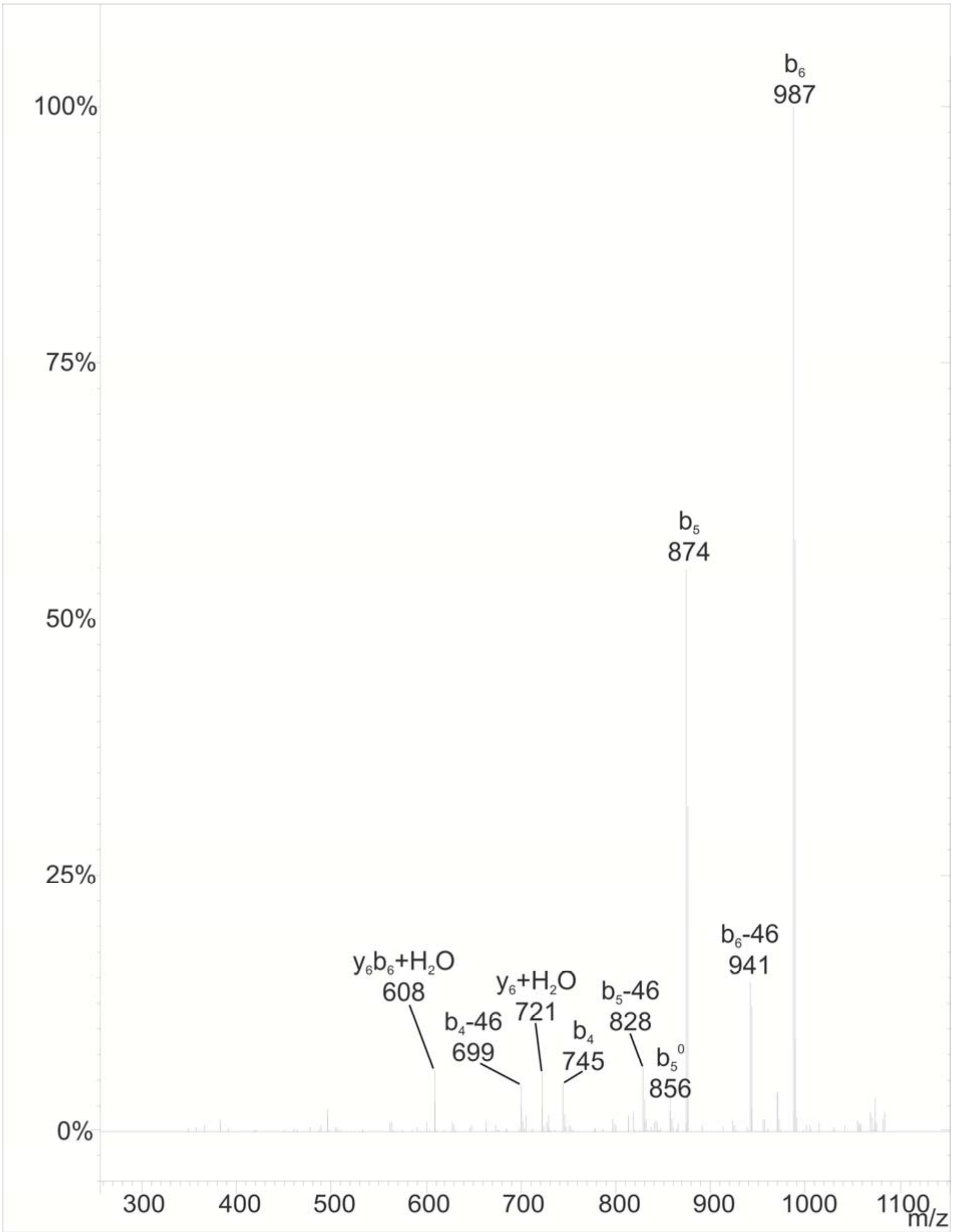


**Figure S41.** The MS[2] spectrum of **41** (C17-[AME5]) eluted at Rt = 92.24 min (*m/z* = 1100).

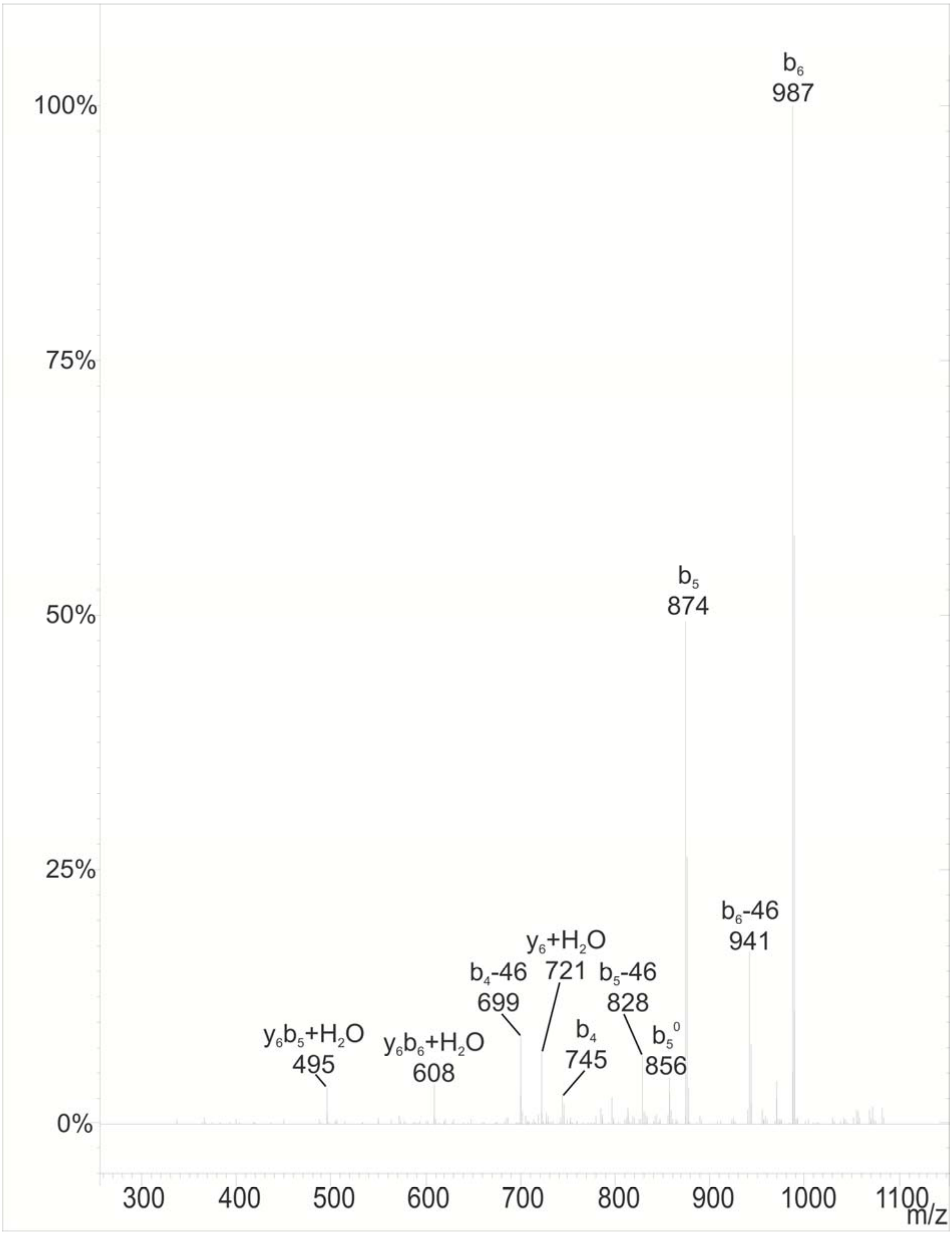


**Figure S42.** The $MS^2$ spectrum of **42** (C17-[AME5]) eluted at Rt = 93.06 min ($m/z$ = 1100).

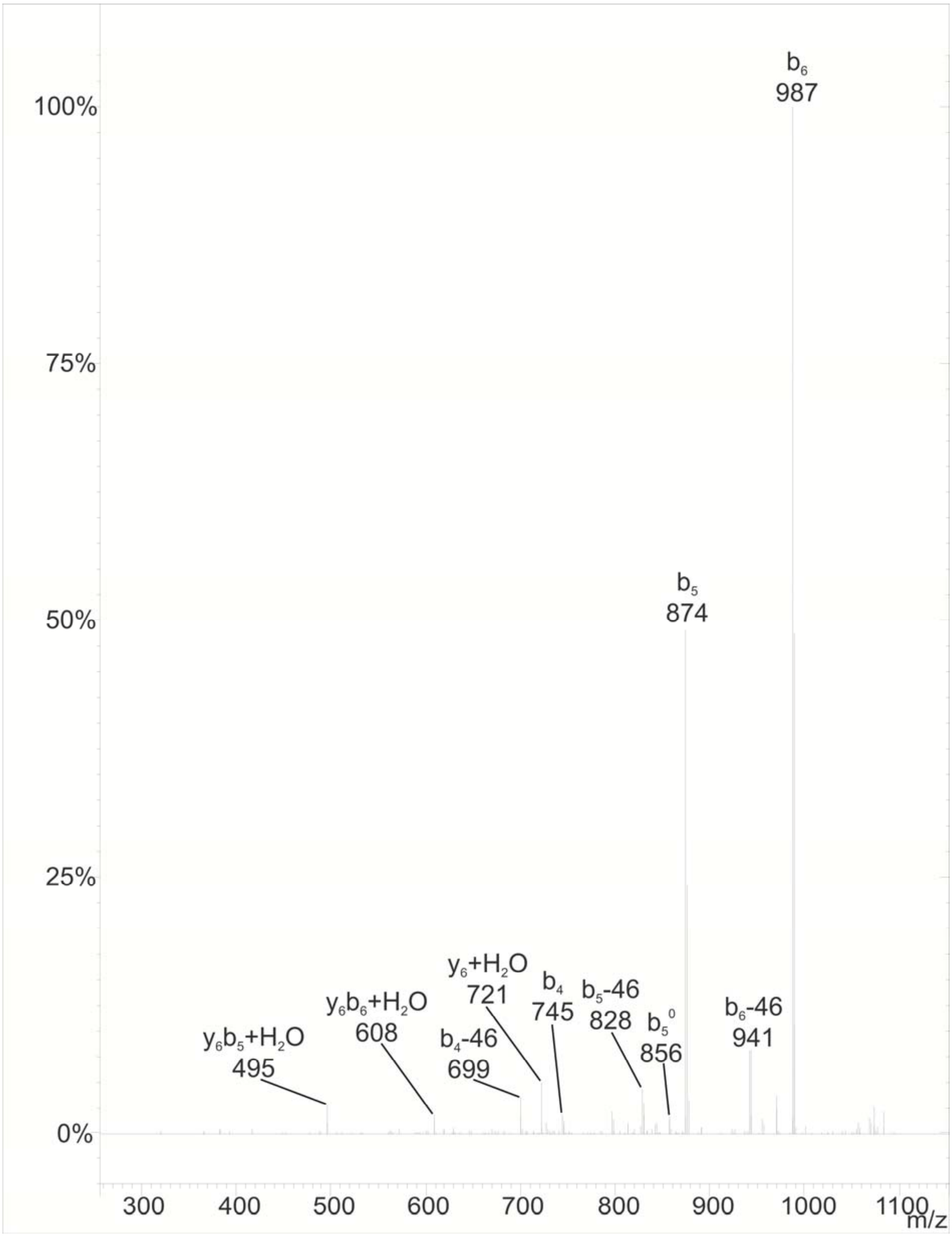


**Figure S43.** The MS$^2$ spectrum of **43** (C17-[AME5]) eluted at Rt = 93.96 min (*m*/*z* = 1100).

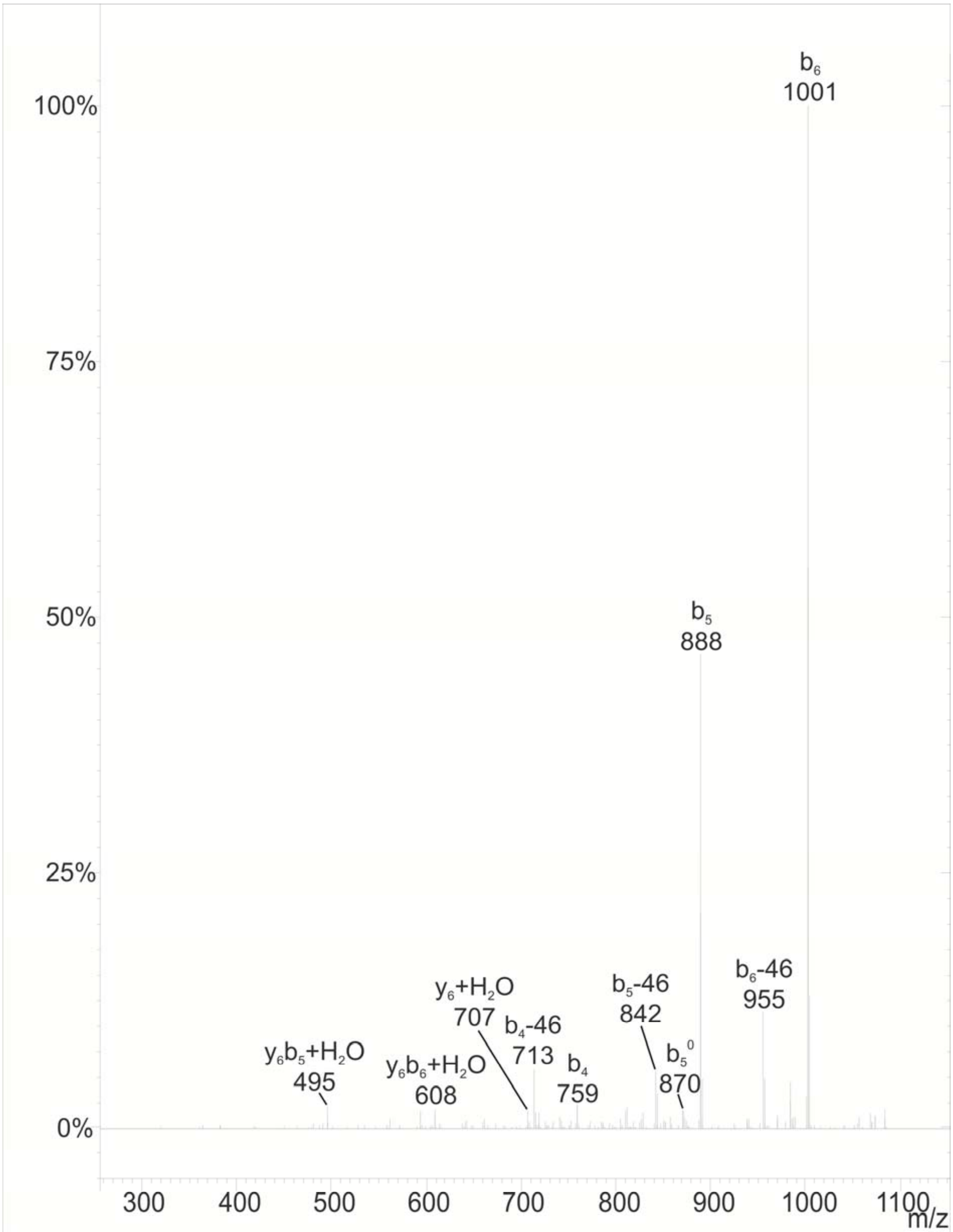


**Figure S44.** The MS[2] spectrum of **44** (C18-[AME5,Val7]) eluted at Rt = 94.71 min (*m*/*z* = 1100).

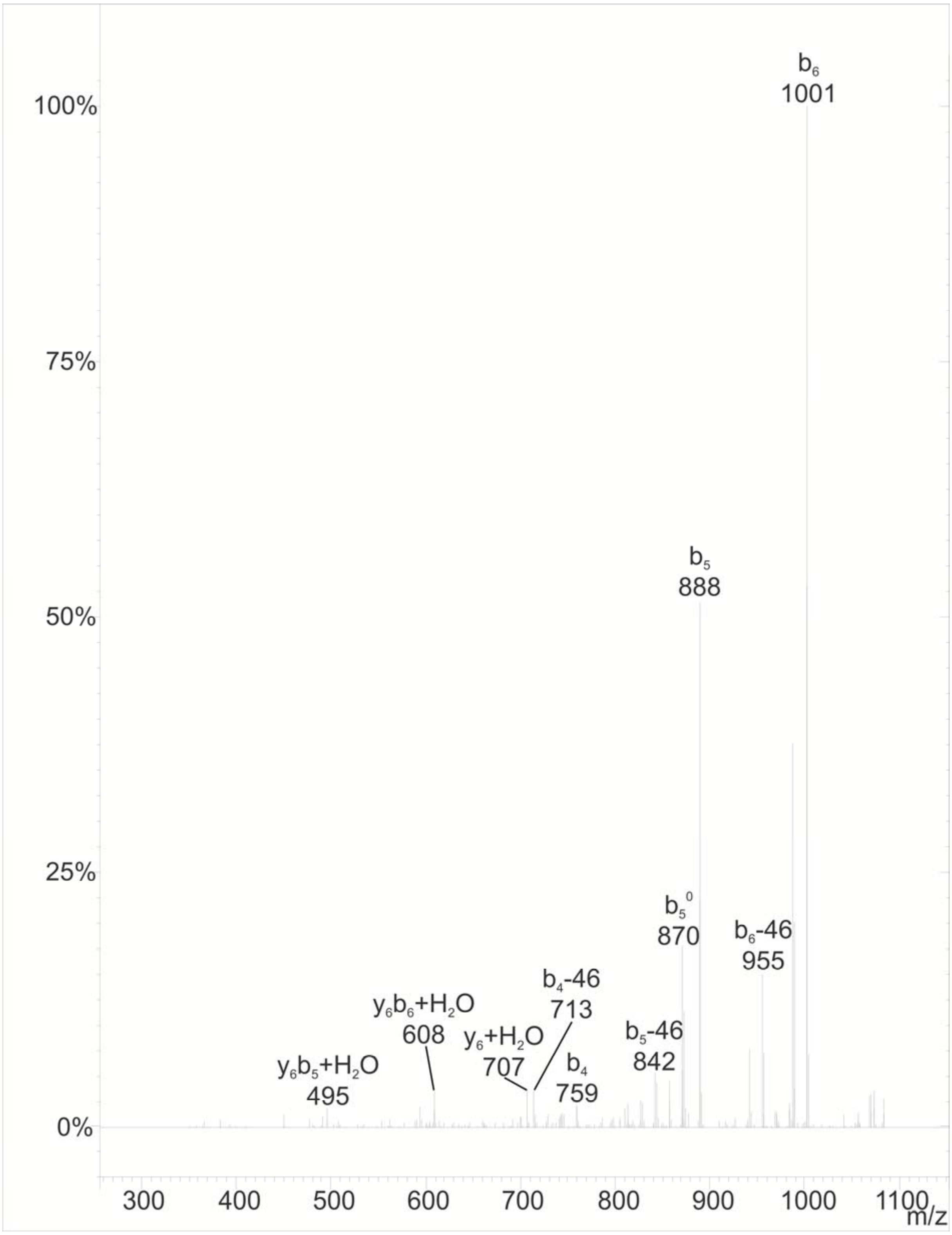


**Figure S45.** The MS[2] spectrum of **45** (C18-[AME5,Val7]) eluted at Rt = 95.80 min (*m*/*z* = 1100).

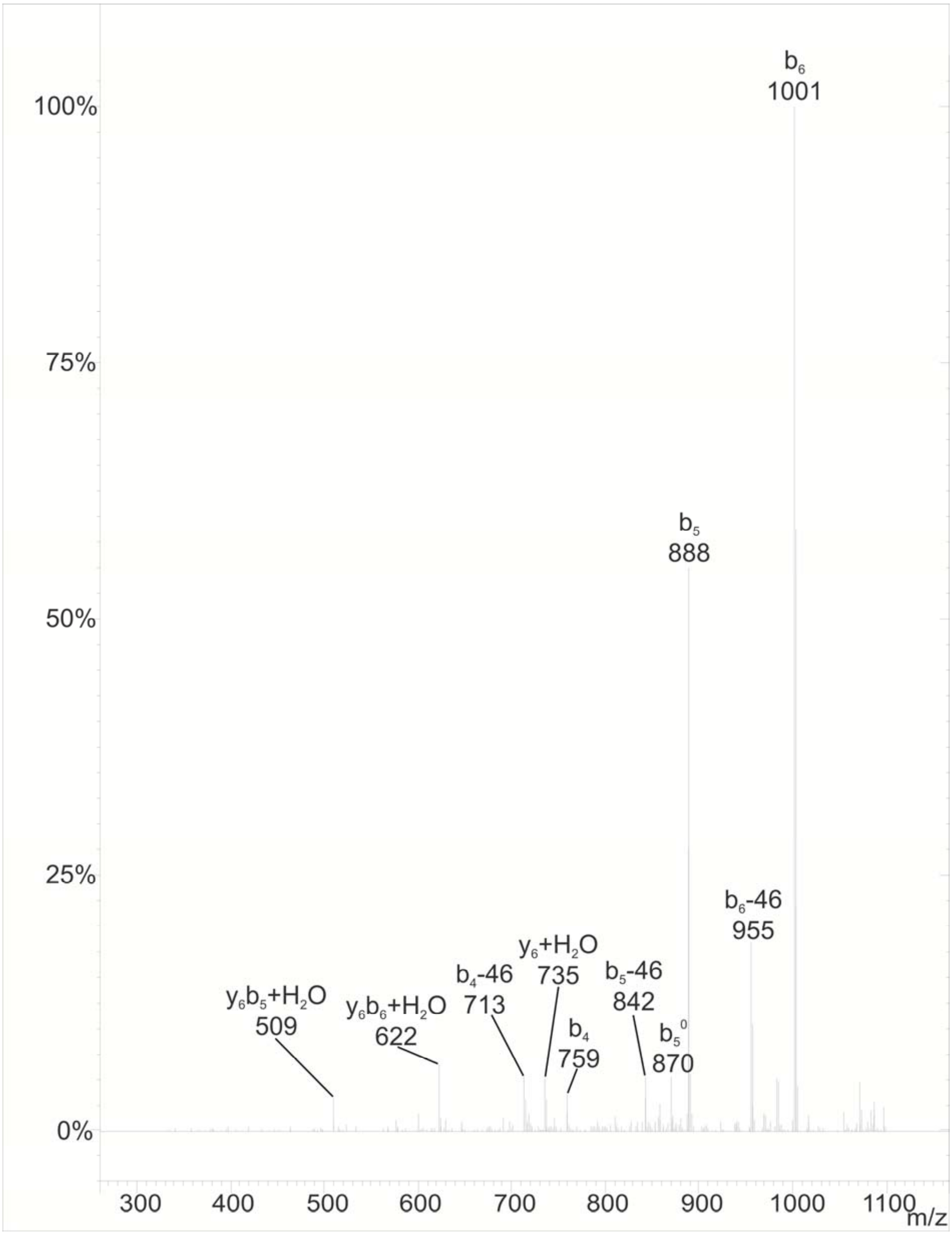


**Figure S46.** The MS[2] spectrum of **46** (C17-[Leu4,AME5]) eluted at Rt = 93.56 min (*m*/*z* = 1114).

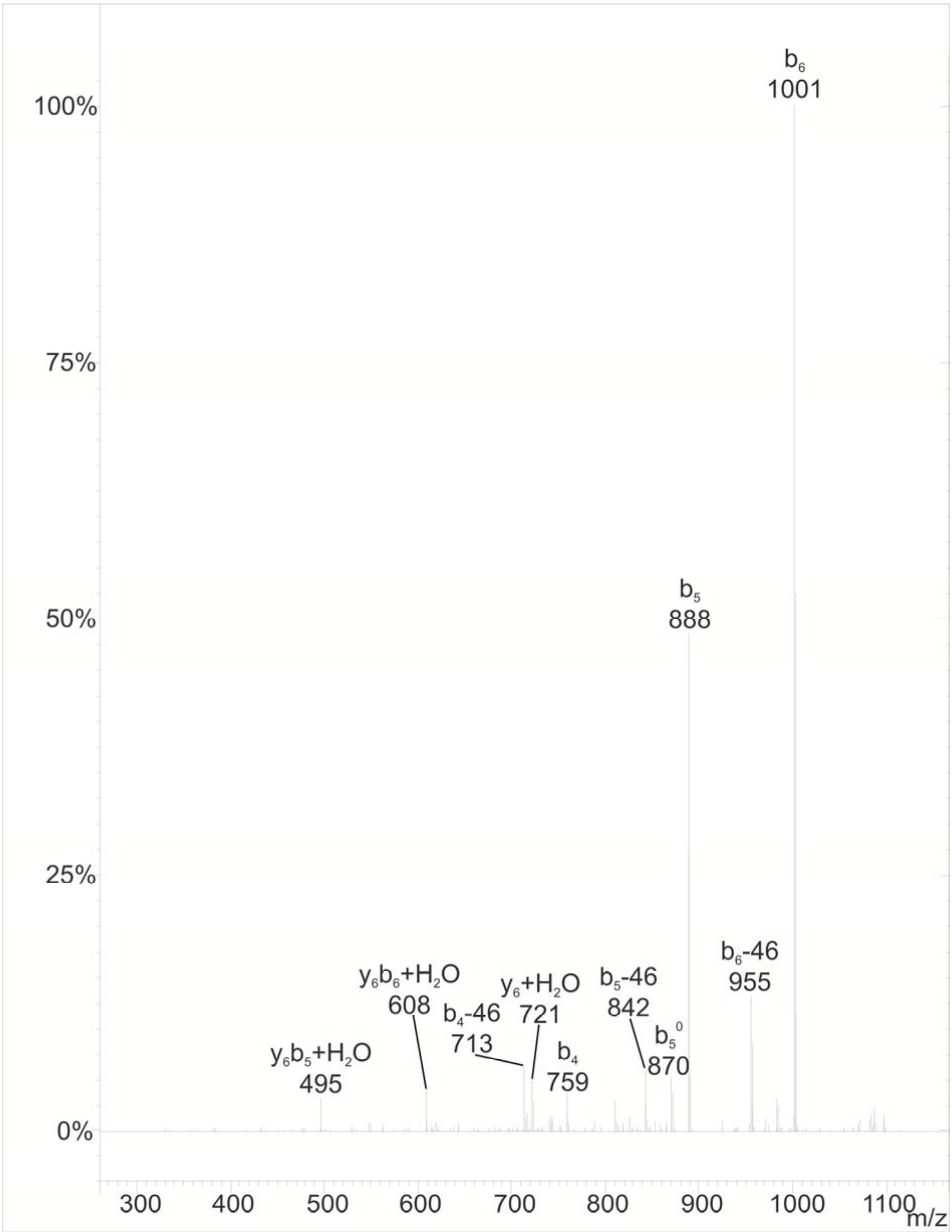


**Figure S47.** The $MS^2$ spectrum of **47** (C18-[AME5]) eluted at Rt = 94.39 min ($m/z$ = 1114).

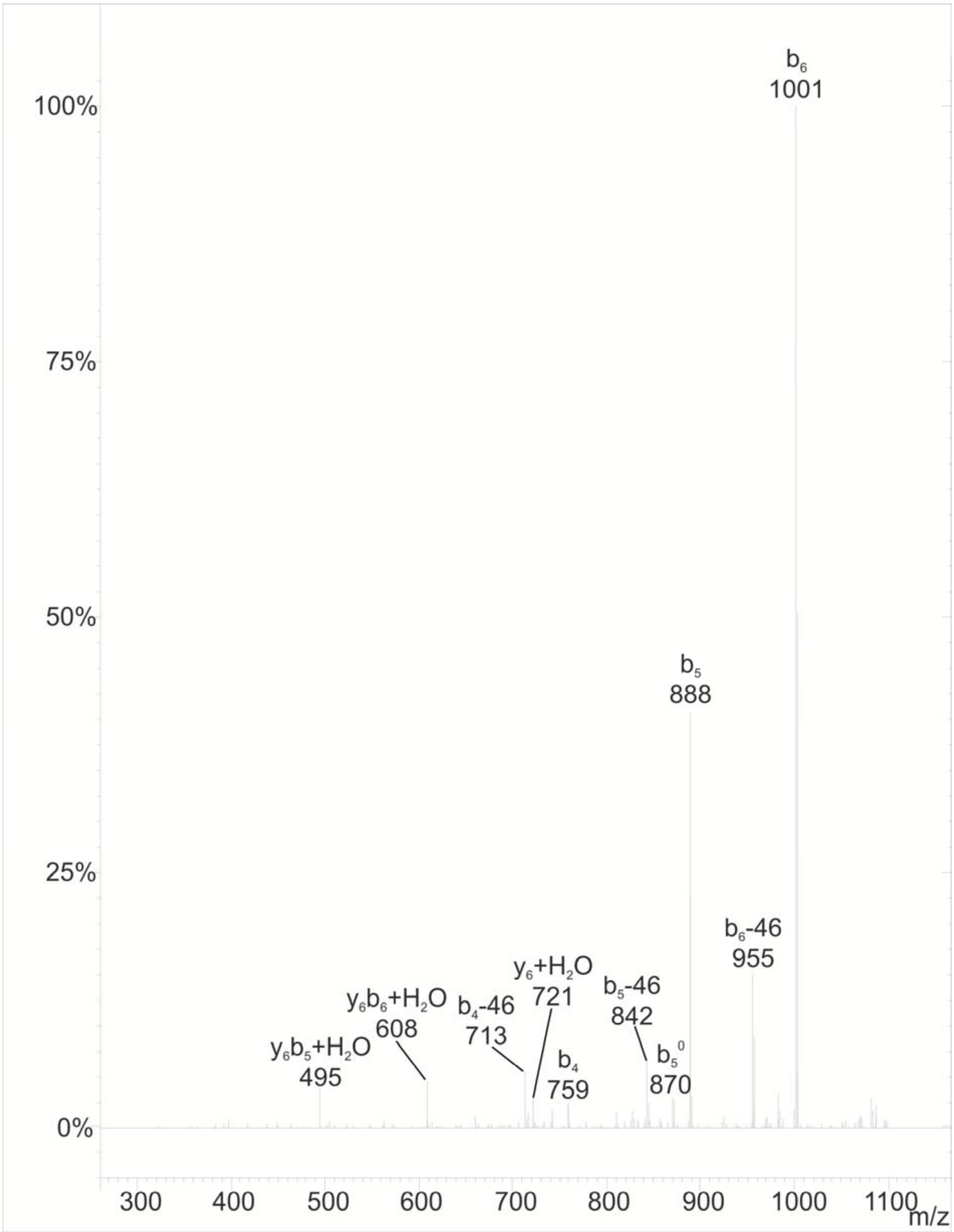


**Figure S48.** The $MS^2$ spectrum of **48** (C18-[AME5]) eluted at Rt = 95.24 min ($m/z$ = 1114).

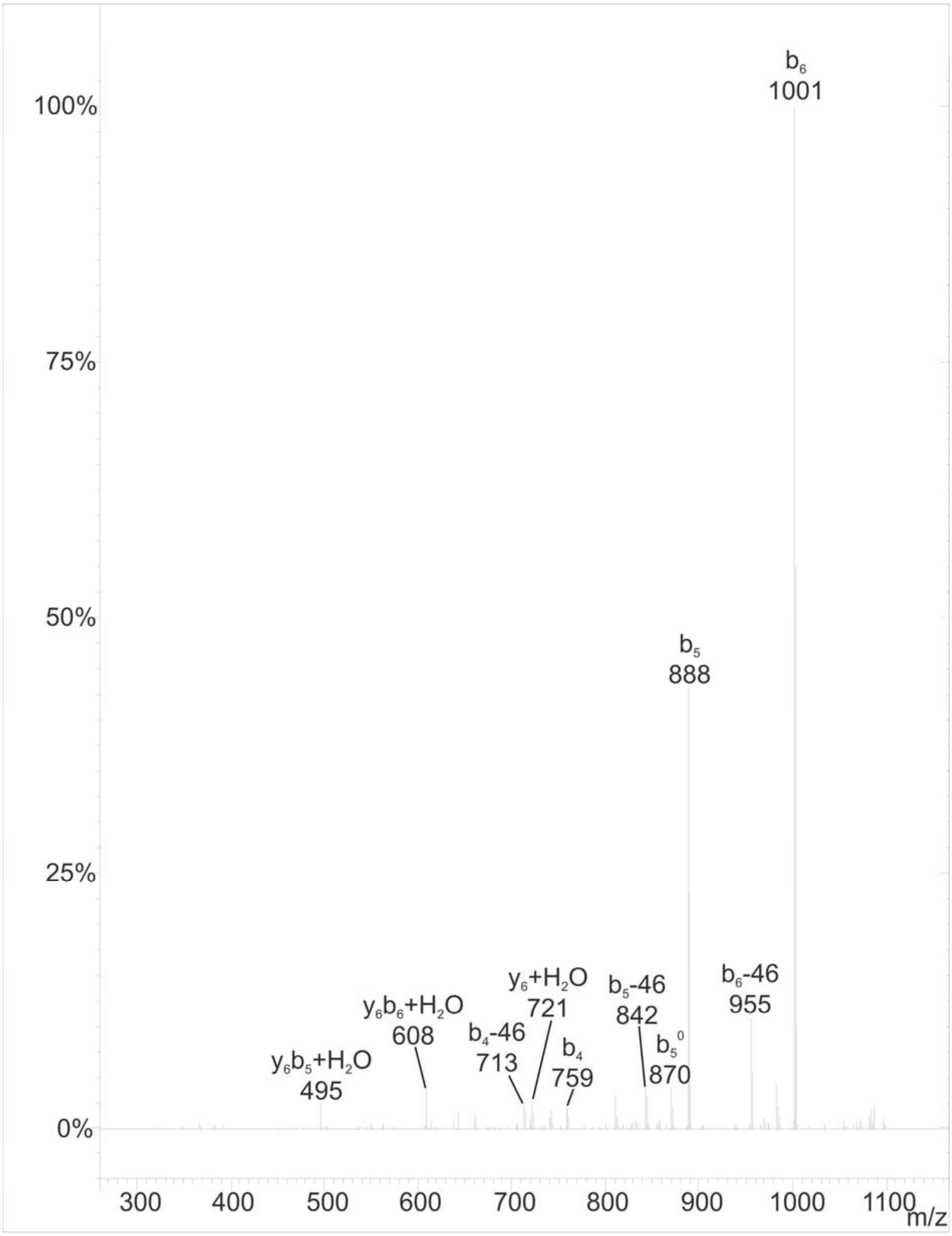


**Figure S49.** The MS[2] spectrum of **49** (C18-[AME5]) eluted at Rt = 96.01 min (*m*/*z* = 1114).
